# Synthetic translational coupling element for multiplexed signal processing and cellular control

**DOI:** 10.1093/nar/gkae980

**Published:** 2024-11-11

**Authors:** Hyunseop Goh, Seungdo Choi, Jongmin Kim

**Affiliations:** Department of Life Sciences, Pohang University of Science and Technology, 77 Cheongam-ro, Pohang 37673, Gyeongbuk, Korea; Department of Life Sciences, Pohang University of Science and Technology, 77 Cheongam-ro, Pohang 37673, Gyeongbuk, Korea; Department of Life Sciences, Pohang University of Science and Technology, 77 Cheongam-ro, Pohang 37673, Gyeongbuk, Korea

## Abstract

Repurposing natural systems to develop customized functions in biological systems is one of the main thrusts of synthetic biology. Translational coupling is a common phenomenon in diverse polycistronic operons for efficient allocation of limited genetic space and cellular resources. These beneficial features of translation coupling can provide exciting opportunities for creating novel synthetic biological devices. Here, we introduce a modular synthetic translational coupling element (synTCE) and integrate this design with *de novo* designed riboregulators, toehold switches. A systematic exploration of sequence domain variants for synTCEs led to the identification of critical design considerations for improving the system performance. Next, this design approach was seamlessly integrated into logic computations and applied to construct multi-output transcripts with well-defined stoichiometric control. This module was further applied to signaling cascades for combined signal transduction and multi-input/multi-output synthetic devices. Further, the synTCEs can precisely manipulate the N-terminal ends of output proteins, facilitating effective protein localization and cellular population control. Therefore, the synTCEs could enhance computational capability and applicability of riboregulators for reprogramming biological systems, leading to future applications in synthetic biology, metabolic engineering and biotechnology.

## Graphical Abstract

**Graphical Abstract:**
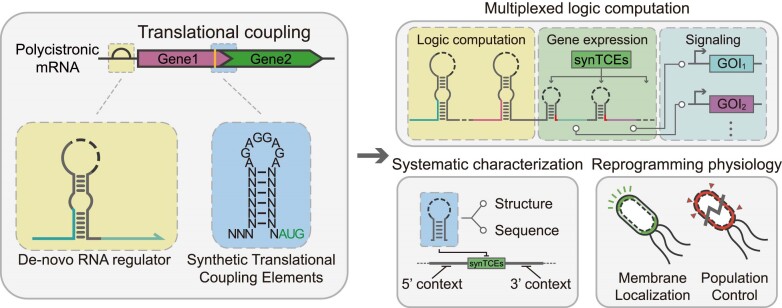

## Introduction

Synthetic biology aims to implement user-defined functions in biological systems by rational design and composition of natural and synthetic regulatory elements. These synthetic biological devices have shown promising demonstrations in several areas including biosensing, therapeutics and molecular computing (1–4). To implement sophisticated functions in the cell, genetic regulatory parts at multiple hierarchical levels including transcription, translation and degradation steps need to be developed and integrated into genetic circuits (1,2,4). In particular, synthetic RNA regulators provide a promising route to enrich the synthetic biological toolkit due to the well-characterized thermodynamic properties amenable to structure prediction and the straightforward engineering process by computational methods (5,6) for constructing synthetic gene networks (7–9). Synthetic RNA regulators have enabled the precise control over gene expression in response to a broad range of stimuli such as small molecules (10–13), proteins (14), temperature (15), pH (16) and nucleic acids (17). Further, the circuit architecture that colocalizes multiple sensory RNA domains in the same transcript allows for a compact genetic circuitry and the advanced computational capacity for complex functions (18–21). Leveraging such a remarkable versatility, these synthetic RNA devices have provided important toolkits in dynamic metabolic engineering (22–24) and point of care diagnostics (25–27).

The synthetic RNA regulators demonstrated so far typically require specific RNA sequence contexts to achieve desired functionality. As such, the overlap of sequence domains for the regulator and the downstream open reading frame (ORF) could result in a limited insulation of gene expression and the output proteins with extended N-terminal ends for certain riboregulator designs (10,25,28–31). Sequence modification at the N-terminus of the target protein could steer the localization pattern (32,33), alter the protein arrangement (34) and impact the protein stability (35–40), and therefore, an important aspect for engineering RNA devices is to enhance the modularity of synthetic circuit designs.

Another important aspect for a synthetic RNA regulator is the capacity to orchestrate the expression of multiple downstream outputs under the control of diverse input signals. Multiplexed production of a designated combination of proteins can provide an extra capacity for gene space (41), an efficient resource usage (42,43) and the systemic robustness with conserved stoichiometry (44–47) with applications in a homeostasis control of pathway-specific enzymes (48), and a compact system configuration (49,50) for differential expression. Despite the advantages, the controlled multi-output production with synthetic RNA-based regulators remains a challenge, partly due to the location of RNA regulator proximal to the downstream ORF to be regulated, where the remaining further downstream ORFs are not subject to the same regulatory strategy. Previous works to address the independent production of multiple output proteins including the introduction of self-cleaving peptides (51) or trans-cleaving endopeptidases (52) can cause alteration in the lifespan of protein (39), the efficiency of translation (51) and the rates of ribosome drop-off (53). Consequently, an effective strategy to produce multiple independent proteins with controlled stoichiometry in conjunction with synthetic RNA devices remains a challenge.

**Figure 1.**
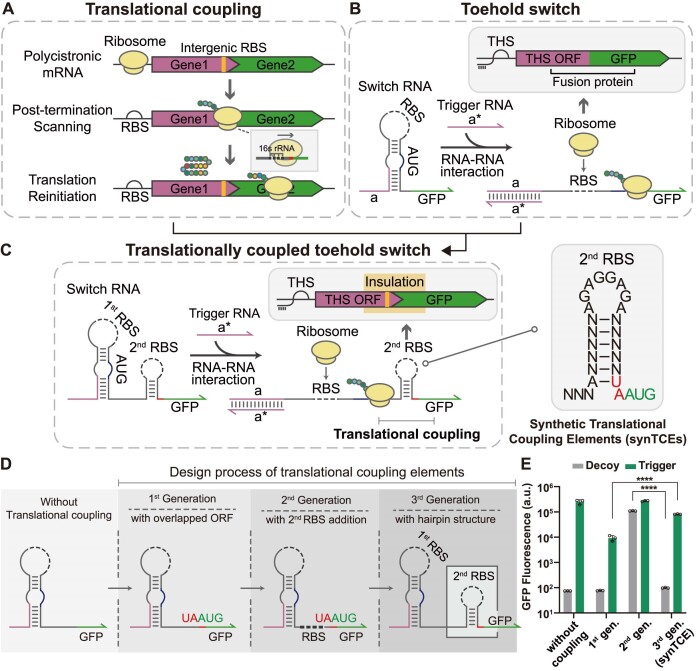
Mechanism and demonstration of a synthetic translational coupling element (synTCE). (**A**) Mechanism of translational coupling. Translating ribosomes from the first ORF proceed until reaching the stop codon of the first gene while unfolding the intergenic region in the process. Post-termination ribosomes from the upstream ORF scan for the adjacent start codon of the second gene and subsequently reinitiate the translation step. (**B**) Schematic of the toehold switch mechanism. The interaction between switch and trigger RNAs can unwind the structure of toehold switch via strand displacement, resulting in the translation of target protein. (**C**) Schematic of a translationally coupled toehold switch. Translation by the ribosome unwinds the secondary structure of synTCE to expose a secondary RBS, and thus, the distal gene can be produced through translational coupling. (**D**) Design process of synTCE. The final configuration of synTCE consists of a six-nucleotide hairpin structure, the RBS and overlapping stop–start codons. The RBS is displayed as a black dashed line. (**E**) Flow cytometry green fluorescence protein (GFP) fluorescence measurement of the toehold switch without modification or with translational coupling elements. Decoy represents an input RNA that does not interact with the switch RNA. Measurements were taken 3 h 30 min after induction with 0.1 mM isopropyl β–D-1-thiogalactopyranoside (IPTG). Dot points specify the individual data, bars represent mean ± standard deviation (s.d.) of *n* = 3 biological replicates. Two-tailed student’s *t*-test; ****: *P* < 0.0001.

To address the design challenges for synthetic RNA devices, we turn to the translation coupling mechanism in polycistronic mRNA. In a polycistronic operon where RNA secondary structures near the translation initiation sites for individual cistrons prevent the translation events, the ribosomes must translate the upstream ORF and permit the unfolding of these structured mRNA regions to facilitate the translation of the downstream genes (54–57). A termination and reinitiation step or an upstream-dependent *de novo* initiation can occur in these translation coupling events depending on the intergenic distance within a polycistronic mRNA. When the cistrons are close or overlapping, the post-termination ribosome from the upstream ORF could scan for the adjacent start codon of downstream ORF and subsequently reinitiate the translation step (Figure 1A). This adjacent configuration of the ORF and intergenic ribosome binding site (RBS) was shown to promote this mode of translation (55,58–60). Building on these results, the mechanism of coupled translation has been repurposed to design operons (61), achieve reliable gene expressions (62), monitor *in vivo* translation steps (63), screen RBS domains (64–66) and engineer riboswitches (67). Therefore, elucidating the design principles for integrating translational coupling in synthetic RNA devices could greatly enhance the functionality and applicability of these RNA regulators.

In this work, we introduce synTCE, designed to enhance the precision and versatility of synthetic gene networks by enabling multiplexed protein production and modular control of gene expression. By systematically investigating key internal and external sequence features, we established design principles that guide the effective implementation of synTCE. These elements were successfully integrated into diverse logic computing devices and synthetic operons, demonstrating their capacity to faithfully transmit regulatory signals and control multiple downstream genes with programmed stoichiometry. Additionally, synTCE enables precise regulation of protein N-terminal sequences, facilitating the targeted subcellular localization of proteins and allowing for signal-responsive control of cell population. By expanding the functional scope of synthetic RNA regulators, synTCE provides a versatile and reliable platform for building next-generation synthetic biological devices. The characteristic features of synTCE to coordinate complex genetic circuits with precisely controlled, multiplexed outputs can provide a powerful tool for advancing applications in metabolic engineering, biotechnology and synthetic biology, opening new possibilities for designing sophisticated gene networks and enhancing cellular functions.

## Materials and methods

### Materials, strains and growth conditions

DNA oligonucleotides were purchased from Bionics (Seoul, Korea). Reagents used for *Escherichia coli (E. coli)* cultivation were purchased from Gibco (Waltham, MA, USA). The following *E. coli* strains BL21 DE3 [F^−^*omp*T *hsd*S_B_ (r_B_^−^ m_B_^−^) *gal dcm*], BL21-AI^TM^ [F^−^*omp*T *hsd*S_B_ (r_B_^−^ m_B_^−^) *gal dcm* araB::T7RNAP-*tet*A], DH5α [*endA1 recA1 gyrA96 thi-1 glnV44 relA1 hsdR17* (r_K_^−^ m_K_^+^) λ^−^], MG1655 (F^−^ λ ^−^*rph*-1) and Nissle1917 were obtained from Bionics, Invitrogen (Carlsbad, CA, USA) and American Type Culture Collection (Manassas, VA, USA). All strains were grown in Luria–Bertani (LB) medium at 37°C with appropriate antibiotics: ampicillin (100 μg/ml), spectinomycin (50 μg/ml), kanamycin (50 μg/ml) and chloramphenicol (25 μg/ml). All antibiotics were purchased from Gold biotechnology (St. Louis, MO, USA).

### Plasmid construction

The backbones for the plasmids used in this research were taken from the commercial vectors pET15b (ampicillin resistance, ColE1 origin), pCDFDuet (spectinomycin resistance, CDF origin), pACYCDuet (chloramphenicol resistance, p15A origin) and pCOLADuet (kanamycin resistance, ColA1 origin) from EMD Millipore (Burlington, MA, USA). All the toehold switches with synTCEs were constructed in pCOLADuet using toehold switch variants (Supplementary Tables S1 and S2). The switch, trigger and receiver constructs used in the signaling cascade module were constructed in pCOLADuet, pCDFDuet and pET15b/pACYCDuet, respectively. Plasmids were constructed using polymerase chain reaction (PCR), Gibson assembly (68) and Golden gate assembly (69,70). DNA templates for synTCE were annealed via gradient cooling or were amplified via PCR, and then inserted into plasmid backbones using BsaI-HFv2 restriction enzyme (New England Biolabs, Ipswich, MA, USA). The DNA sequences of effector proteins Ecf11_987 (#49661) and LuxR (#48885) and a synthetic promoter Pecf11_3726 (#49707) were cloned using the plasmids from Addgene (Watertown, MA, USA) as templates. Lytic genes, including holin (S105), endolysin (R) and spanin (Rz, Rz1), were synthesized by Integrated DNA Technologies (IDT, Coralville, IA, USA). All plasmids were cloned in the *E. coli* DH5α strain, purified using the EZ-Pure^TM^ plasmid Prep Kit. Ver. 2 (Enzynomics, Daejeon, Korea). Plasmid sequences were confirmed via BTseq^TM^ (Celemics, Seoul, Korea) based on a low-depth next-generation sequencing technique or Sanger sequencing (Bionics) after every cloning step. Plasmid architecture and specific part sequences are listed in Supplementary Tables S1–S4. Representative complete plasmid sequences are described in Supplementary Table S5.

### Design of synTCE

All design processes were performed using Biopython and NUPACK 4 python packages with the following parameters: material = “rna06”, ensemble = “stacking", celsius = 37, sodium = 1.0, magnesium = 0.0 (6,71). In addition, RNA sequences containing AAAA, CCCC, GGGG, UUUU, KKKKKK, MMMMMM, RRRRRR, SSSSSS, WWWWWW and YYYYYY were set as pattern constraints for prevention of the same or similar nucleotides in a row, and thereby improving design quality (72). For the design of synTCE, overlapping stop–start codons (5′–URAUG–3′) and a purine-rich RBS sequence (5′–AGAGGAGA–3′) were employed, and stem domains with designated lengths (more than 6 bp) were randomized. The design results with no in-frame stop codons prior to overlapping stop–start codons were selected using translation function of Biopython. The detailed source code for the design of synTCE is described in the Supplementary Method.

### Cell culture and induction condition

For *in vivo* experiments, we used *E. coli* BL21 DE3 and AI strains with chromosomally integrated T7 RNA polymerase under the control of the IPTG-inducible lacUV5 promoter and the arabinose-inducible pBAD promoter, respectively. For most of the experiments that use *E. coli* BL21 DE3 strain, chemically transformed *E. coli* BL21 DE3 cells were cultured on 1.5% LB agar plates (BD Biosciences, Franklin Lakes, NJ, USA) with appropriate antibiotics. Single colonies were grown overnight (∼16 h) in 96-deep well plates with shaking at 800 rpm, 37°C. Overnight cultures were diluted 1/100-fold into fresh medium and returned to an orbital shaker (800 rpm, 37°C; Cat# SHLDMP03DG, OHAUS, Parsippany, NJ, USA). After 80 min, cell cultures were induced with 0.1 mM IPTG (Promega, Madison, WI, USA) and returned to the shaker (800 rpm, 37°C) until fluorescence measurement after 3 h 30 min. For multi-output transcripts and signaling cascade modules, chemically transformed *E. coli* BL21 AI cells were cultured on 1.5% LB agar plates with appropriate antibiotics. All the experimental procedures were identical when using the *E. coli* BL21 AI strain, except that arabinose (Gold Biotechnology) was used as an inducer. For the experiment for signaling cascade using TetR and Ecf11, after the 1/100-fold dilution step, the incubation time was extended from 80 min to 3 h to allow for sufficient accumulation of reporter outputs.

### Flow cytometry measurement

GFP fluorescence was measured by flow cytometry (CytoFLEX S, Beckman Coulter, Brea, CA, USA) after fixation at the Microbiome Core Research Support Center of the Korea Basic Science Institute (KBSI). The cell pellet was resuspended with the 2% (w/v) para-formaldehyde solution (Sigma Aldrich, St. Louis, MO, USA) and fixed for 15 min at room temperature. After fixation, samples were stored at 4°C until the flow cytometry analysis. The fixed cells were washed twice with 1× phosphate-buffered saline [PBS (pH = 7.2); Cat# EBP006-1000, Enzynomics]. Fixed cells were diluted by a factor of 6 into 1× PBS. Cells were detected using a forward scatter (FSC) trigger and at least 50000 events were recorded for each measurement. The cell population was gated according to the FSC and side scatter distributions as described previously (73). To evaluate the circuit outputs, the fluorescence of GFPmut3b-ASV was measured on a fluorescein isothiocyanate (FITC) channel, excited with a 488-nm laser and detected with a 525/40-nm bandpass filter. The fluorescence of mCherry was measured on the ECD/mCherry channel, excited with a 561-nm laser, and detected with a 610/20-nm bandpass filter. GFP and mCherry fluorescence histograms yielded unimodal population distributions, and the geometric mean was employed for the average fluorescence across the approximately log-normal fluorescence distribution from three biological replicates.

### Bacterial cell imaging

The constructs for the signal responsive localization control were expressed via T7 RNA polymerase in the *E. coli* BL21 DE3 strain. All the procedures for cell culture preparations were identical to other fluorescence measurements. After induction, cells were washed with 1× PBS, and 10 μl of cell cultures were mounted with clotted 1% agarose gel to observe the cell shape by fluorescence imaging with a Zeiss Axio Scope with EC PLAN NEOFLUAR (Zeiss, Oberkochen, Germany) using 100× objective lenses. Digital images were taken using the AxioCam HRM camera and processed with AxioVision 4.8 software. Image sampling was performed across multiple different locations for each cell collection of three biological replicates. Raw images were converted to 8-bit black/white images and an Unsharp mask filter [radius: 2-pixel, mask weight: 0.5; ImageJ (v1.54d)] was applied. The brightness and contrast of images were automatically adjusted. The cell collection was automatically acquired via the MicrobeJ plugin (v5.13o) (74). Classification of GFP localization was conducted using the raw data from intensity plots of acquired cell collections. Detailed classification criteria of GFP localization are described in Supplementary Method. Demography was automatically generated via the MicrobeJ from the acquired cell collection.

### Cell viability assay

To construct a lysis circuit, phage lysis associated genes (S, R, Rz and Rz1) were cloned into a plasmid containing toehold switch variants. *E. coli* MG1655 cells containing a lysis circuit, a control circuit and corresponding empty plasmids were then transferred to 1 ml of LB with 0.2% glucose (Cat# G8270-1KG, Sigma Aldrich) in a 96 deep-well plate (Cat# 503102, NEST, Palo Alto, CA, USA) and grown in an orbital shaker at 30°C and 800 rpm overnight. Overnight cultures were then diluted 1/20-fold into a 96-well cell culture plate (Cat# 31096, SPL, Gyeonggi-do, Korea) and grown in a BioTek Synergy H1 microplate reader (BioTek Gen5, Santa Clara, CA, USA). When the OD600 value of the culture reached 0.6∼0.8, 0.5 mM IPTG was treated to all samples. Samples were collected after 7 h induction and were washed twice with 1× PBS, and then serially diluted in 1× PBS over a 7-log range. Then, 5 μl of washed samples were spotted onto LB agar plates with appropriate antibiotics. The plates were incubated at 30°C overnight before imaging. Cell viability (CFU/ml) was calculated by the following formula:


\begin{equation*}{\rm CFU}/{\rm ml} = \left( {{\rm number} \ {\rm of} \ {\rm colonies}} \right)\ \times \left( {{\rm dilution} \ {\rm factor}} \right)\ /\ 0.005\ {\rm ml} \end{equation*}

## Results

### Design of synTCEs integrated with toehold switches

Natural translational coupling elements can form the basis to engineer novel translation coupling devices. However, a simple adoption of natural translational coupling elements to synthetic riboregulators may not result in ideal performance. First, to precisely regulate the translation initiation step with a detailed mechanistic understanding, we chose the toehold switch as a model synthetic RNA translation regulator. The toehold switch RNA contains an RBS within a strong hairpin stem and the start codon in the bulge such that ribosome access is limited. In the presence of trigger RNA, an RNA–RNA interaction between the switch and trigger RNA disrupts the secondary structure surrounding the RBS and start codon, thereby enabling the ribosome to access the RBS and initiate translation (Figure 1B). Due to the capacity for precise regulation of translation states of downstream elements, toehold switches can serve as suitable upstream devices for incorporation within new design schemes for translation coupling. The finely tunable translation states by adjusting trigger RNA levels could facilitate the exploration of factors that affect translational coupling activity in the new synthetic devices. In the toehold switch design with a translation coupling element, a rationally designed RNA device for translational coupling is placed downstream of the toehold switch where the second ORF is in a different reading frame (Supplementary Figure S1). In this synthetic translational coupling scheme, the ribosome loading at the first RBS within the toehold switch initiates the downstream translation process where the ribosome ultimately reaches the translation coupling element, subsequently unwinding any secondary structure to expose the intergenic RBS to help reinitiate the translation process (Figure 1C).

To construct a synthetic RNA device for translational coupling, we initially designed adjoining stop–start codon configurations between the toehold switch and the GFP coding sequence (first generation in Figure 1D and Supplementary Figure 2A). Multiple designs, including adjacent ORF configuration such as overlapped (‘UAAUG’ and ‘AUGA’) and proximal stop–start codons, were constructed to test whether these designs could provide translational coupling dependent on the upstream translation controlled by the toehold switch activity. As expected, these designs showed low signals when the upstream toehold switch was in the OFF state with a decoy RNA input, whereas the GFP outputs were strongly increased when the upstream toehold switch was in the ON state with a cognate trigger RNA (Figure 1E and Supplementary Figure S2B). However, this translation coupling design showed an ON-state output signal an order of magnitude less than that of the unmodified toehold switch, indicating inefficient translational coupling. To further explore these design features, we used the ‘URAUG (R indicates A or G)’ overlapped stop–start codon configuration, which showed consistent results for multiple toehold switch variants without requiring any modification in the downstream ORF (Supplementary Figure S2C).

To enhance ribosome loading at the downstream gene, a second RBS was introduced a few bases upstream of the overlapped stop–start codon (second generation in Figure 1D). The introduction of the second RBS would likely facilitate the post-termination ribosome to initiate translation of the downstream gene. The GFP fluorescence output indeed increased for this design; however, the leakage signal in the absence of the trigger RNA was almost as high as the ON-state signal (Figure 1E). This result suggested that *de novo* translation initiation at the second RBS was quite efficient, precluding effective translational coupling under the control of the upstream translation event.

To reduce putative *de novo* translation initiation at the overlapped stop–start codon, a stable secondary structure was introduced surrounding the second RBS (third generation in Figure 1D). A strong hairpin structure of synTCE would prevent *de novo* translation initiation of the downstream gene. However, successful ribosome loading at the first RBS initiates the translation of downstream regions such that the secondary structure of synTCE would be unwound by the ribosome, exposing the second RBS for the effective reinitiation of translation (Figure 1D and Supplementary Figure S1). Consequently, this design can suppress leakage expression from *de novo* initiation and improve the translational coupling efficiency controlled by the activation of the upstream translation element. The resulting synTCE design showed robust performance with a large dynamic range albeit with a slight reduction compared to the unmodified toehold switch (Figure 1E).

### Characterization and optimization of synTCEs

We next aimed to further characterize design features that affect the efficiency of translational coupling in synTCE. First, several synTCE variants with the same RBS, but with modified sequences in the hairpin stem and different locations of stop codons were constructed (Supplementary Figure S3 and Supplementary Table S4). Not surprisingly, the sequence elements within the hairpin stem and the location of stop codons with respect to the RBS in the loop led to variable performance of synTCE despite the identical predicted secondary structures for the design variants.

**Figure 2.**
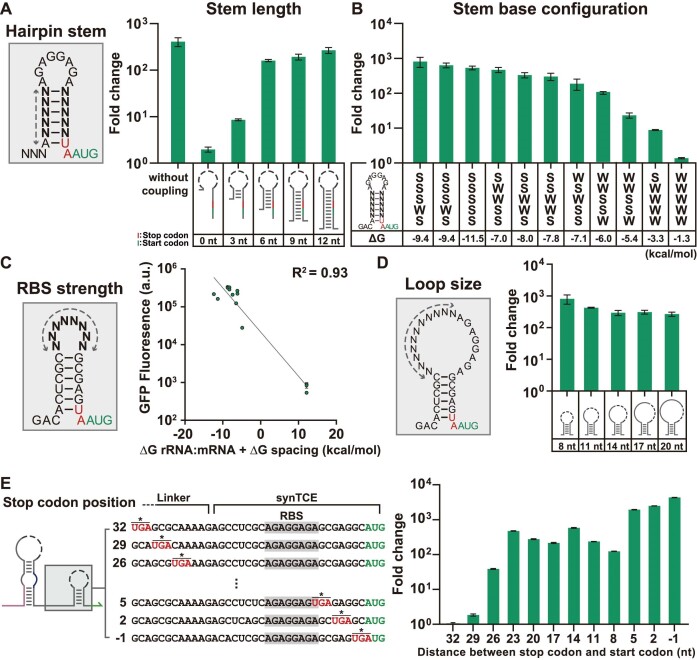
Characterization and optimization of synTCE. (**A**) Fold change in GFP fluorescence levels of stem length variants of synTCE. (**B**) Fold change in GFP fluorescence levels of synTCE with adjustments in the base compositions for a 6-bp stem. S and W indicates nucleotides with strong (G, C) and weak (A, U) interactions, respectively. The calculated ΔG value of stem is displayed underneath each stem variant. (**C**) Correlation between the RBS strength and GFP fluorescence output. RBS strength is evaluated by the sum of ΔG rRNA:mRNA and ΔG spacing. The line indicates the result of simple linear regression. (**D**) Fold change in GFP fluorescence levels of synTCE with loop size variants. (**E**) The positional effect of the stop codon on translation coupling efficiency. Schematics represent the location of stop codon in the sequence. Flow cytometry GFP fluorescence measurements were taken 3 h 30 min after induction with 0.1 mM IPTG. Fold change is the ratio of the geometric mean of the GFP fluorescence level for the ON and OFF states. The relative errors for the ON and OFF states are from the s.d. of biological triplicate. Relative errors for GFP fold change were obtained by adding the relative errors of the ON- and OFF-state fluorescence measurements in quadrature. Bars represent mean ± relative error of *n* = 3 biological replicates.

Thus, we sought to systematically examine both the internal and external sequence elements of synTCE. For the stem region of the hairpin, we first adjusted the length of the synTCE stem between 0 and 12 nucleotides. The leakage expression, presumably from *de novo* initiation at the RBS of synTCE, was strongly inhibited with 6 bp and longer stem variants, resulting in high fold changes of reporter expression (Figure 2A). Interestingly, longer stem variants with up to 12 bp showed little reduction in the GFP output when compared to the 6-bp stem, possibly due to the highly efficient ribosomal helicase activity (75). Still, the minimal 6-bp stem architecture was chosen for further exploration to enhance design flexibility. To investigate the required thermodynamic energies for a stable structure to achieve robust performance, we systematically adjusted the base composition of the 6-bp stem with the associated ΔG values calculated by NUPACK (Supplementary Table S4C). The leakage expression of synTCEs was effectively reduced in stems with multiple strong base pairs presumably by preventing *de novo* initiation by the ribosome (Figure 2B). In addition, the ΔG value of the stem was also correlated with the fold change of synTCEs (Supplementary Figure S4).

Next, we investigated whether sequence modification to the loop region of synTCE affects the efficiency of translation reinitiation. We first randomized the loop region of synTCE that contains the RBS sequence (Supplementary Figure S5A). As a result, sequence modification in the loop significantly influences GFP fluorescence output, implying that disruption of the RBS sequence in synTCE affects the efficiency of translation reinitiation at the coupled junction. To demonstrate the correlation between RBS strength and GFP output, we used parameters representative of RBS strength such as ΔG rRNA:mRNA and ΔG spacing from the RBS calculator (76) (Supplementary Table S4D), where each parameter specifies the interaction between the 3′ end of 16S rRNA and the RBS of mRNA, and the effect of the distance between the RBS and start codon, respectively. Predicted RBS strength (ΔG rRNA:mRNA + ΔG spacing) showed a strong correlation with the GFP fluorescence output of synTCE (Figure 2C; R^2^= 0.93). In addition, we extended the loop size from 8 to 20 nucleotides to identify the limits of engineering the hairpin structure (Figure 2D and Supplementary Figure S5B). SynTCEs showed tolerance to the loop extension, despite some reduction in dynamic ranges possibly due to an increased accessibility to the RBS or an altered sequence context near RBS (77).

The distance between the stop codon of the upstream ORF and the start codon of the downstream ORF is a crucial factor for translational coupling (60,61,78). Indeed, the GFP expression of synTCE variants appear to be affected by the distance between the stop and start codon (Supplementary Figure S3). To closely probe whether the intergenic distance influences the performance of synTCE, we tested several variants with different spacing between the stop codon and the start codon (Figure 2E). The translational coupling was highly effective for variants where the stop codon and the start codon were closely located (−1 to 5 bases of intergenic distance), and a gradual decrease in the dynamic range was observed as the distance between the stop and start codon is increased. Additionally, we evaluated the effect of local mRNA structure on post-termination scanning of ribosome (59,79). The local mRNA structure was strongly correlated with the GFP fluorescence level in our analysis (See Supplementary Method, Supplementary Figures S6 and S7; R^2^= 0.98).

Next, multiple sequence domains upstream and downstream of synTCE were investigated. Several toehold switches were introduced upstream of synTCE where all the toehold switches exhibited large dynamic ranges with at least 186.1-fold activations (Supplementary Figure S8A). Further, to test the impact of the output sequence context, we fused the first 36 nucleotides of various proteins upstream of the sfGFP output reported to minimize misfolding when fused to arbitrary polypeptides (80) (Supplementary Table S4). When combined with synTCE, the fluorescence outputs of sfGFP were generally unaffected, indicating the robust performance of synTCE for diverse sequence contexts (Supplementary Figure S8B). The adjustment of the linker sequence domain between the toehold switch and synTCE showed little impact on the dynamic range (Supplementary Figure S8C). For very long linkers (> 1 kb), however, the difference between the ON and OFF states gradually decayed, until the two states converged for linkers longer than 3 kb (Supplementary Figure S9). This may be due to the increased number of putative RBS within the linker sequences and the potential for ribosome drop-off events for long linkers (81). By limiting expression variability for multiple sequence contexts, the synTCE could function as an insulator analogous to the bicistronic design strategy (62).

### Integration of multiplexed logic computing devices with synTCE

**Figure 3.**
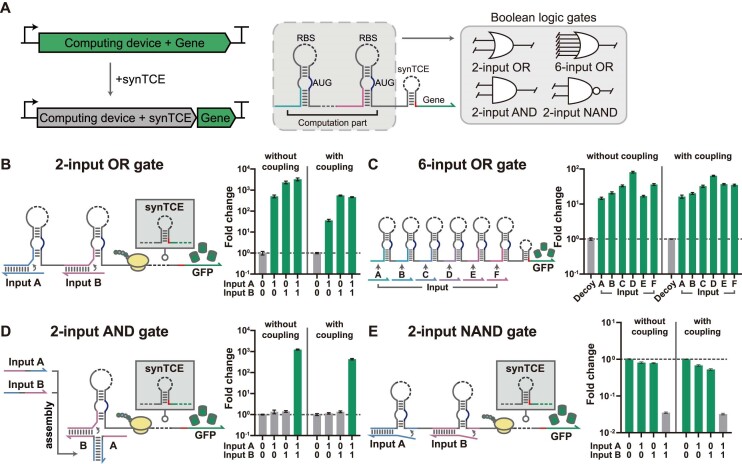
Integration of synTCE with multi-input logic computing devices. (**A**) Schematic of multi-input signal processing devices integrated with synTCE. The integrated devices can implement Boolean logic operations and transduce the upstream regulation to downstream elements. (**B–E**) Schematic and fold change plot of translationally coupled ribocomputing devices for a two-input OR gate (**B**), a six-input OR gate (**C**), a two-input AND gate (**D**) and a two-input NAND gate (**E**). The input RNA combinations are displayed underneath the fluorescence graph. The letters for RNA inputs correspond to toehold switch modules within the gate RNA, except for the two-input AND gate. Flow cytometry GFP fluorescence measurements were taken 3 h 30 min after induction with 0.1 mM IPTG. Fold change is calculated as the ratio of the geometric mean of GFP fluorescence level for the ON and OFF states. The relative errors for the ON and OFF states are from the s.d. of biological triplicates. Relative errors for GFP fold change were obtained by adding the relative errors of the ON- and OFF-state fluorescence measurements in quadrature. Bars represent mean ± relative error of *n* = 3 biological replicates.

In natural systems, translational coupling is often used to implement logic computation of target gene expression by introducing regulatory components that control the translation of the upstream ORF (58,82,83). When applied to synthetic circuits, translation coupling can provide a seamless method to integrate regulatory functions at the input layer and transmit them to the output layer with multiplexed expression controls. Ribocomputing devices that concatenate several toehold switches and utilize the self-assembly of input RNAs could provide a convenient platform for integrating a multiplexed signal processing unit at the input layer (18–20,26,30). To check whether synTCE can mediate faithful signal transduction from the input layer to the output layer, we examined several Boolean logic computation modules combined with synTCE designs (Figure 3A and Supplementary Table S2).

To implement a two-input OR gate, two switch RNAs were concatenated in the same reading frame upstream of the GFP output with or without synTCE (Figure 3B and Supplementary Figure S10). The input RNAs target their cognate switch RNA elements to disrupt the hairpin structure to expose the RBS, thereby allowing translation of the downstream elements. Notably, the ribosome loading on the upstream RBS allows unwinding of downstream switch hairpins such that the input A allows efficient translation of GFP output much like the input B for the two-input OR gate. Thus, any cognate RNA, A or B, can activate the GFP output to perform OR logic (Figure 3B). The introduction of synTCE in the two-input OR gate architecture showed a robust performance with up to 460.2-fold activation albeit with slightly reduced fold changes for the ON states. The OR gate architecture was scaled up to include six sensor domains and still exhibited a robust performance when the synTCE was incorporated with over 10-fold activation for each cognate input RNA (Figure 3C).

To demonstrate a two-input AND gate, the cognate input RNA was divided into two separate domains, the toehold binding (input B) and the branch migration (input A), with the additional domains to allow the self-assembly of the two separated domains. The expression of individual input RNA A or B cannot effectively disrupt the switch hairpin structure, whereas the expression of both A and B allows the formation of a complete trigger sequence (Figure 3D). As the presence of both input RNAs is required for switch activation, the system functions as an AND logic gate. The introduction of synTCE in the two-input AND gate showed the robust performance with 432.1-fold activation for the ON state when compared to the null inputs (Figure 3D).

For a NOT logic implementation, a three-way junction (3WJ) repressor switch was taken for use with the synTCE architecture. A 3WJ repressor possesses a weak hairpin structure surrounding the RBS and the start codon such that the switch RNA is translationally active. However, in the presence of a cognate trigger RNA, a stable 3WJ structure forms, preventing the access of the ribosome to the RBS and the translation of the downstream output. Therefore, the 3WJ repressors can carry out the NOT gate operation as desired (Supplementary Figure S11). The incorporation of synTCE in the 3WJ repressors exhibited over 20-fold repression in the presence of trigger RNAs. Further, multiple 3WJ repressors can be concatenated using the same strategy as the OR gate architecture. In this design, with only one input RNA present, translation of the output gene will continue from the unrepressed hairpin module due to the helicase activity of the ribosome unwinding downstream secondary structures. Thus, the gate RNA functions as a NAND gate that can be inactivated only when both input RNAs are present (Figure 3E). The NAND gate architecture with synTCE exhibited over 10-fold repression when both inputs were present. In addition, an alternative strategy of using antisense input RNA could implement a NOT gate. For instance, an antisense RNA (input B) with extended overhangs can effectively sequester the trigger RNA (input A), whether free or bound to the gate RNA (18,30). Introducing the synTCE into a two-input NIMPLY gate showed a robust performance with a 78.6-fold reduction in the OFF state compared to the ON state (Supplementary Figure S10).

Together, ribocomputing devices with synTCE demonstrated the capability to faithfully transmit the multiplexing functionality of ribocomputing designs with toehold switches to the downstream elements.

### Harnessing synTCEs to implement multi-output signal processing devices

Multiple-output signal processing devices through translational coupling can allow for a robust control of stoichiometry with efficient resource usage, and a sophisticated architecture with minimized coding space for the genetic circuit. Taking advantage of synTCE to transduce the upstream signal into the downstream elements, we aimed to construct a multi-output controller. By concatenating multiple ORFs using synTCE variants, multiple output signals can be controlled in response to the regulation of upstream switches.

**Figure 4.**
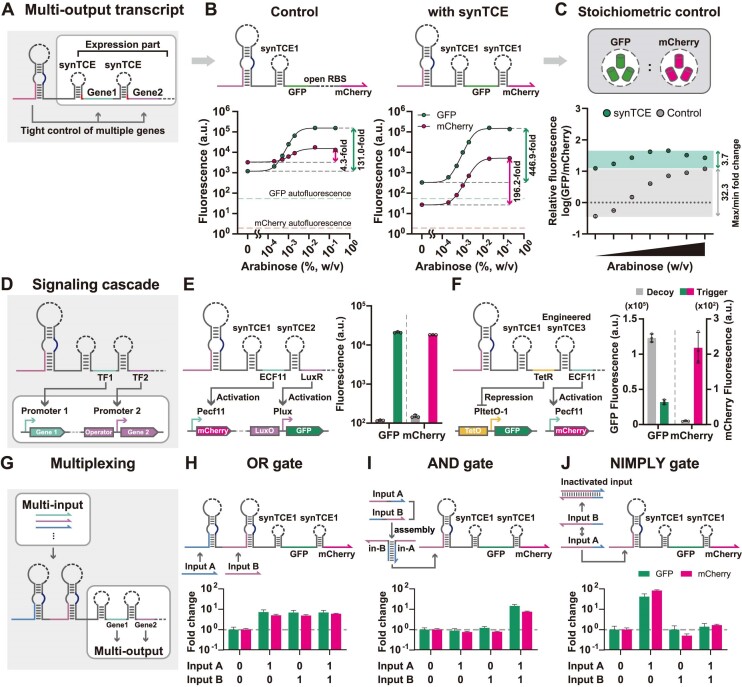
Implementation of multiplexed signal processing synthetic operons using synTCE. (**A**) Schematic of a multi-output transcript. Multiple downstream genes are concatenated together with synTCEs for multiplexed control of output proteins. (**B**) The GFP and mCherry reporter expressions for a control construct with an open RBS for the second reporter, mCherry and the multi-output transcript are compared as the input RNA levels are increased by arabinose induction. Measurements were taken 5 h after induction with 0.1 mM IPTG and 0%, 0.0002%, 0.0005%, 0.001%, 0.002%, 0.02% and 0.2% of arabinose. (**C**) Comparison of response curves in terms of the relative ratio of GFP to mCherry fluorescence. (**D–F**) Multiplexed signaling cascades for transcription factors. Ecf11_987 and LuxR (**E**) or TetR (**F**) were utilized as effector proteins. Dot points specify the individual data, bars represent mean ± s.d. of *n* = 3 biological replicates. (**G–J**) Multiplexed synthetic operon architectures for multi-input/multi-output logic computing devices. A two-input OR gate (**H**), a two-input AND gate (**I**) and a two-input NIMPLY gate (**J**) that control GFP and mCherry outputs were demonstrated. Fold change is the ratio of the geometric mean of the GFP and mCherry fluorescence level for the ON and OFF states. The relative errors for the ON and OFF states are from the s.d. of biological triplicate. Relative errors for GFP and mCherry fold changes were obtained by adding the relative errors of the ON- and OFF-state fluorescence measurements in quadrature. Bars represent mean ± relative error of *n* = 3 biological replicates. The experimental measurements used *E. coli* BL21 AI [panels (A–F)] or BL21 DE3 [panels (G–J)] strains.

To test whether the synTCE can provide the multi-output signal processing capacity, we first designed architectures for a multi-output transcript using GFP and mCherry reporter genes either with or without synTCE (Figure 4A). As the trigger RNA levels were increased via arabinose induction, the expression of GFP and mCherry were tracked for both designs (Figure 4B). In the control construct where the *de novo* translation events can occur due to an open RBS, the mCherry reporter expression showed a high basal expression and a limited increase upon activation of the upstream toehold switch and GFP reporter expression presumably due to the limited translation coupling. On the contrary, for the multi-output transcript with synTCE, the mCherry and GFP expressions showed a strong correlation with almost constant fold-changes across a wide range of trigger RNA levels.

Furthermore, this design led to the proportional protein synthesis for the coupled downstream genes such that the relative stoichiometric ratio of GFP to mCherry was largely maintained (Figure 4C). This stoichiometric balance of outputs in the synTCE-based architecture could be further tuned by RBS engineering (Supplementary Figure S12) and synTCE variants (Supplementary Figure S13). Therefore, the linkage of multiple downstream genes with synTCE architecture provides a faithful signal transmission from the upstream riboregulator to the downstream genes and the capability for controlling the stoichiometric ratio of multiple genes through coordinated translational coupling activity. We also confirmed that the capability to process multiple outputs can be extended by constructing a three-output transcript consisting of GFP, mCherry and tagBFP reporter proteins, exhibiting 155.3-fold, 28.1-fold and 31.0-fold activation, respectively (Supplementary Figure S14).

Utilizing the capability of synTCE to generate multi-output signals, we next sought to create multi-output mediated signaling cascades (Figure 4D). As a starting point, we constructed single-output cascades that can activate or repress the next layer using LuxR (84,85), Ecf11 (86) and TetR as effector proteins (Supplementary Figure S15A). Cascades with Ecf11 and LuxR demonstrated the activation of final outputs by 182-fold and 14-fold, respectively (Supplementary Figure S15B), while the cascade involving TetR showed an initial surge in the downstream signal and a subsequent repression over 10-fold (Supplementary Figure S15C). Further optimization through the fusion of an ssrA degradation tag to effector proteins improved the dynamic range of these cascades (Supplementary Figure S16). Based on the characterization, we next built a dual-activating cascade with synTCE-linked Ecf11 and LuxR, showing the activation of downstream targets by 196.8-fold for Ecf11 and 106.7-fold for LuxR (Figure 4E). A cascade with Ecf11 and TetR showed a 21.5-fold activation of mCherry fluorescence by Ecf11 and a 3.84-fold repression of GFP fluorescence by TetR (Figure 4F). Time course analysis indicated a rapid repression of GFP expression by TetR and a slower activation of mCherry fluorescence by Ecf11 (Supplementary Figure S17). Further, to explore the application of synTCE in signaling cascades, synTCEs with different RBS strengths were used for the second transcription factor while keeping the same synTCE for the first transcription factor. This modified signaling cascade exhibited almost constant mCherry fold changes under the control of the first transcription factor, whereas GFP fold changes increased from 8.82-fold to 504.76-fold as the RBS strengths of the second synTCEs increased (Supplementary Figure S18). Thus, the synTCE architecture can be used to tune the stoichiometry of transcription factors as output proteins, and thereby successfully repurposed for controlling signaling cascades.

The synTCE architecture provides a means to construct multi-input/multi-output logic circuits for scaling up the complexity of synthetic circuits. To test this possibility, we designed and tested two-input OR, AND and NIMPLY gates with GFP and mCherry outputs via synTCE. The robust performance for multi-input/multi-output logic circuits was demonstrated, indicating the potential for increased complexity in synthetic circuit designs (Figure 4G–J and Supplementary Figure S19). Taken together, we demonstrated that synTCEs can be configured to expand the capability to control multiple output signals and function as compact modules for constructing sophisticated synthetic networks.

### Precise regulation of protein N-terminal ends via synTCE

Reprogramming the cellular physiology is an attractive target for synthetic RNA devices. For instance, phenotypic changes, including protein localization patterns, control of cellular motility and colorimetric changes, were achieved by synthetic riboregulators (19,87). Still, certain riboregulator designs that use linker domains between the regulatory region and downstream protein-coding region produce output proteins with extended N-terminal ends (19,20,88,89). While minor changes in the N-terminus can be accommodated for many proteins, proteins with key signal sequences at the N-terminal residues require an alternative regulatory strategy (26).

To test the impact of the synTCE architecture on the precise control of the N-terminal ends of downstream proteins, we performed SDS-PAGE analysis of histidine-tag purified output proteins with and without synTCE (Supplementary Figure S20A). The output GFP under the control of synTCE showed the same migration pattern as the GFP control, unlike the GFP output under the control of an unmodified toehold switch. Similarly, the GFP output under the control of a six-input OR gate also displayed the same bands as the GFP control for different input RNA combinations when integrated with synTCE (Supplementary Figure S20B).

**Figure 5.**
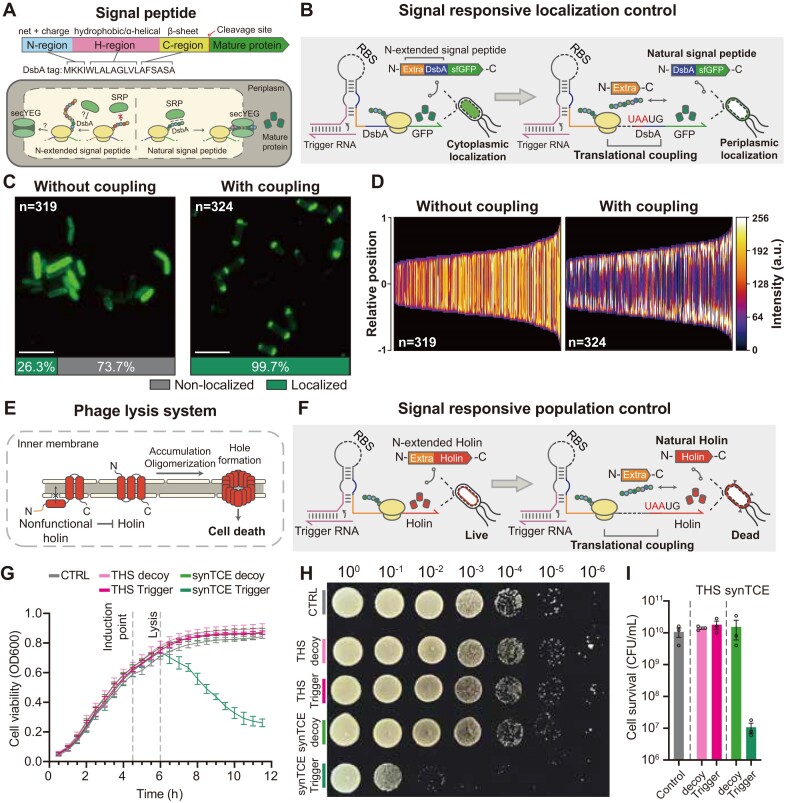
Improved cellular control via precisely controlled protein N-terminal ends by synTCE. (**A**) Schematic of the structure and mechanism of the DsbA signal peptide. SRP indicates the signal recognition particle. (**B**) Schematic of the DsbA signal peptide-tagged superfolder GFP (sfGFP) under the control of a toehold switch with and without synTCE. (**C**) Representative fluorescence microscopy image of GFP distribution in cells. Scale bar = 5 μm. The fluorescence image was taken 3 h 30 min after induction with 0.1 mM IPTG. (**D**) Demographic representation of GFP intensity measured along the medial axis of the cells. Cells are sorted by cell length. Y-axis indicates the relative position along the cell body, where 0 represents the center of the cell. (**E**) Mechanism of holin in bacteriophage λ lysis system. N-terminal peptide determines the insertion of the transmembrane domain into the inner membrane. (**F**) Schematic of holin cassette production under the control of a toehold switch with and without synTCE. (**G–I**) Experimental results for a signal responsive lysis circuit in *E. coli* MG1655: time-course profiles for cell density (**G**), representative image of cell survival assay (**H**) and a bar graph for colony-forming units (CFU) (**I**). Holin expression was induced with 0.5 mM IPTG after 4 h 30 min of cell growth. Samples for the CFU assay were collected 7 h after the induction with IPTG. For cell survival assay (**H**), the samples were serially diluted by 10-fold (from 10^−1^ to 10^−6^) and spotted on LB agar plate, where the dilution factors are indicated above the image. THS: toehold switch without synTCE. CTRL: control. Dot points specify the individual data, bars represent mean ± s.d. of *n* = 3 biological replicates.

This ability to precisely control N-terminal ends for downstream proteins can be utilized for the control of cellular protein localization. For instance, N-terminal signal peptides direct the cellular localization for both natural and synthetic systems with defined peptide properties (Figure 5A) (90,91). Thus, we chose a protein disulfide oxidoreductase DsbA N-terminal signal peptide-tagged GFP as the regulatory target of synthetic riboregulator with and without synTCE architecture, where the DsbA-tagged protein is expected to localize within the periplasm (92,93). The DsbA-tagged GFP would localize at the periplasmic area if the N-terminal signal peptide was recognized, whereas the fluorescence signal would be distributed throughout the cell if the signal peptide was not recognized by cellular machinery (Figure 5B). The DsbA-tagged GFP produced under the control of a toehold switch exhibited localization in 26.3% of cells without synTCE, while 99.7% of cells with synTCE design showed localization in the periplasmic area, indicating a proper recognition of the DsbA signal peptide (Figure 5C and D, and Supplementary Figure S21). Additionally, a precisely controlled localization of output proteins can be achieved by strategic positioning of signal peptides within the synthetic operon. For instance, the reporter proteins, normally distributed in the cytoplasm, can be selectively redirected to membrane by the integration of the DsbA signal peptide (Supplementary Figure S22), suggesting the potential use of synthetic operonic architecture for multiplexed control of membrane trafficking.

To further test the potential of synTCE for biocontainment, we exploited a phage lysis system. The holin-antiholin system was chosen, where holin (S105) plays a critical role in cell lysis by disrupting the inner membrane, but antiholin (S107) inhibits the hole formation (Figure 5E) (94). A notable feature of this system is that the addition of two amino acids to the N-terminus of holin leads to a topological change of the transmembrane domain, resulting in the functional change of holin into antiholin (95,96). Thus, toehold switch-based regulation of holin expression without synTCE would likely show a non-lytic phenotype due to the addition of N-terminal residues, but cell lysis can occur with synTCE designs (Figure 5F). To test this hypothesis, we constructed the expression cassettes to regulate the phage lysis gene cluster (S, R, Rz and Rz1) through a toehold switch with or without synTCE. Upon induction of the phage lysis gene cluster, cell growth measured by absorbance at 600 nm was reduced only when the cognate input RNA was expressed for the synTCE design (Figure 5G). Similarly, cell viability assay showed a reduction of CFUs (Figure 5H and I). The signal responsive lysis circuit also effectively operated in a probiotic strain *E. coli* Nissle1917 (Supplementary Figure S23), suggesting that the cell lysis by holin remained functional under the control of the synTCE architecture.

Collectively, precisely controlled protein N-terminal ends can enhance the applicability of RNA regulators including protein localization or cellular population control and further allow these devices to expand the available range of effectors.

## Discussion

Here, we present the design of synTCE integrated with synthetic riboregulators for cellular logic processing and regulatory functions in *E. coli*. Systematic evaluation of synTCE variants led to the identification of simple design rules for controlling the extent of translation coupling and output expression levels. Further, synTCE designs allowed multiplexing modules for logical and cascaded signal processing in cells. Lastly, the precise regulation of the protein N-terminus with synTCE provided sophisticated control over protein localization and cell population.

The synTCE design presented here can offer several advantageous properties. First, like natural polycistronic operons, synTCE provides the merits of an operonic platform, including robust stoichiometric maintenance and synchronized control of operon members with small encoding space (97). Second, synTCE shows excellent modularity with little dependence on a diverse set of sequence contexts, allowing for seamless integration into well-characterized synthetic riboregulators in a plug-and-play manner. Third, the well-defined N-terminus of the output protein under the control of synTCE allows precise control of downstream protein functionality by simple adjustments in the synTCE domains.

Despite advantageous features, there are certain limitations for current synTCE designs. First, the synTCE can be impaired by ribosome stalling (98) or putative translation initiation sites (Supplementary Figure S9). We observed weak performance for synTCE5 variant encoding two consecutive prolines (PP-motif), which can induce ribosomal stalling and subsequent ribosomal collision, thereby reducing translation efficiency (Supplementary Figure S3) (99). Therefore, improvements in the design pipeline, including the prediction of putative RBS (76) or ribosomal stalling motifs (98), can be beneficial. Second, the introduction of synTCE can lead to reduced dynamic ranges compared to unmodified synthetic riboregulators. Analysis of transcript levels in synTCE showed that ON-state transcript levels were decreased when compared to ON-state toehold switches without synTCE, presumably due to reduced ribosome occupancy (100), which could contribute to lower dynamic ranges for synTCE designs (Supplementary Figure S24). Given that translation termination and subsequent reinitiation events occur with imperfect efficiency (101,102), a mitigating strategy to minimize reduction in dynamic ranges by, for instance, RBS engineering (Supplementary Figure S25) would be desired.

Translational coupling is often utilized in natural and synthetic biological systems. Balanced production of multiple enzymes (50) or coproduction of antibiotics with proteins of interest (64) for metabolic engineering applications showed successful implementation. However, less attention has been given to detailed structural analysis of the translational coupling elements. Systematic exploration of synTCE designs presented here can provide a forward engineering platform of translational coupling elements for fine-tuning the efficiency of several coupling elements within the transcript.

By understanding the fundamental principles of synTCE, RNA-based synthetic biology toolset can be further extended and seamlessly integrated for rational control of multiple proteins in a predetermined way. Multiplexed input and output signal processing capacity of synTCE combined with ribocomputing devices can provide a framework for single cell network motifs (103) and neural networks (104). In addition, output expression levels of synthetic operon architecture could potentially be individually addressed by incorporating interaction sites of small RNAs (105) or RNA-binding proteins (106) near the RBS of synTCE. Further, well-defined stoichiometry among the output proteins could have applications in utilizing toxin–antitoxin strategies (107) and enhancing multimeric complex formation (108). Still, further improvements in the dynamic range of synTCE designs would be desirable for enhanced scalability of synTCE, while also taking into account position-dependent effect and reduction in overall gene expression levels by increased cellular burden for multigene expression (109) (Supplementary Figure S26).

Further, the synTCE design strategy may find use in the optimization of protein expression in combination with other technological developments. For instance, the efficiency of a complex genetic circuit relies on the viability and activity of its enzymes, but the regulatory context surrounding the genes for these enzymes could append unwanted peptides that interfere with their downstream functions. SynTCE enables the expression of proteins without extended N-terminal peptides, thereby mitigating the impact on circuit function (Supplementary Figure S27). In addition, a synthetic protein quality control system used toehold switches to increase the portion of full-length proteins with some additional N-terminal residues (26). Applying synTCE architecture to this protein quality control system would likely maintain high full-length output protein expression with precisely controlled N-terminal ends for cellular localization and functional changes. Another promising area of application is live biotherapeutics, which are currently entering early clinical development utilizing several synthetic regulatory components to optimize delivery of therapeutic outputs and to control population levels (110–112). Precise regulation of multiple protein outputs with defined stoichiometry under synTCE designs could be useful to deliver multiple toxin and therapeutic effector combinations for live biotherapeutics (113,114).

In summary, simple design rules for synTCE were presented for construction of synthetic riboregulators to yield multiplexed logical regulation and control of multiple output proteins. These same designs could be used to control the protein localization and cell viability and may find use in other application areas such as optimization of protein production and live biotherapeutics and integration with other synthetic biological devices such as RNA thermometers (15), pH responsive elements (115) and metabolite sensing riboswitches (67).

## Supplementary Material

gkae980_Supplemental_Files

## Data Availability

The experimental data sets are either included in this submission, the supplemental information or are available from the authors upon request.
