# Supplementary material for: Synthetic translational coupling element for multiplexed signal processing and cellular control
Source: Nucleic Acids Res. 2024 Nov 11;52(21):13469–83. doi: 10.1093/nar/gkae980 (PMC11602170; doi:10.1093/nar/gkae980)
Supplement: gkae980_Supplemental_Files [file gkae980_supplemental_files.zip › NAR_synTCE_source code.docx]

# Import NUPACK Python module

from nupack import *

from Bio.Seq import Seq

# Sequence translation

def sequence_translation(seq):

from Bio.Seq import Seq

bioseq_translation=Seq(seq).translate()

bioseq_translation=str(bioseq_translation)

seq_list=[]

for i in range(0, len(bioseq_translation)):

seq_list.append(f'-{bioseq_translation[i]}-')

seq_translation=''.join(seq_list)

return seq_translation

# Structure prediction

def structure(seq):

seq=str(seq)

m=mfe(strands=[seq], model=my_model)

return str(m[0].structure)

# Specify model

my_model = Model(material='rna06', ensemble='stacking', celsius=37, sodium=1.0, magnesium=0.0)

# Specify Domains

LK = Domain('aacctggcggcagcgcaaaag', name='LK')

N1 = Domain('N1', name = 'N1') # For adjusting protein coding frame.

stem = Domain('NNNNNN', name='N6')

RBS = Domain('AGAGGAGA', name='RBS')

overlapped_codon=Domain('URAUG', name = 'overlapped_codon')

# Define Constraints

my_soft_constraints=[Pattern(['A4', 'C4', 'G4', 'U4', 'M6', 'K6', 'W6', 'S6', 'R6', 'Y6'])]

# Specify Strands

STRAND = TargetStrand([LK,N1,stem,RBS,~stem,overlapped_codon], name='STRAND')

# Specify Complexes

COMPLEX = TargetComplex([STRAND], '......................((((((........)))))).....', name= 'COMPLEX')

# Set a stop condition of 1% and a seed for random number generation to get a reproducible result for this demo

my_options = DesignOptions(f_stop=0.01, seed=0)

# Define and run the complex design job

my_design=complex_design(complexes=[COMPLEX], soft_constraints=my_soft_constraints, options=my_options, model=my_model)

my_result = my_design.run(trials=1)[0]

Designed_sequence=str(my_result.to_analysis(STRAND))

print(Designed_sequence)

print(sequence_translation(Designed_sequence))

print(structure(Designed_sequence))
