# Supplementary material for: Synthetic translational coupling element for multiplexed signal processing and cellular control
Source: Nucleic Acids Res. 2024 Nov 11;52(21):13469–83. doi: 10.1093/nar/gkae980 (PMC11602170; doi:10.1093/nar/gkae980)
Supplement: gkae980_Supplemental_Files [file gkae980_supplemental_files.zip › NAR_synTCE_supplementary_information_R2.pdf]

## Supplementary information for:

# Synthetic translational coupling element for multiplexed signal processing and cellular control

Hyunseop Goh[1,†], Seungdo Choi[1,†], and Jongmin Kim[1,*]

[1]Department of Life Sciences, Pohang University of Science and Technology, Pohang, 37673, Gyeongbuk, Korea

[†]These authors contributed equally.

[*]Correspondence: jongmin.kim@postech.ac.kr; Tel.: +82-54-279-2322

## Table of Contents

### Supplementary Method

1. Design of synthetic translational coupling element
2. Microplate reader analysis
3. SDS-PAGE
4. Bacterial cell classification for GFP localization
5. RT-qPCR analysis
6. Detailed explanation of reinitiation inhibition score

### Supplementary Figure

1. Nucleotide-level schematics of translationally coupled toehold switch
2. Translational coupling through adjacent ORF configuration
3. SynTCE variants showing different performance
4. Correlation between ΔG of stem region of synTCE and GFP fluorescence
5. The result of loop modification of synTCE
6. The result of reinitiation inhibition score in the stop codon tiling assay
7. Detailed schematics for the calculation of reinitiation inhibition score
8. The investigation of surrounding context of synTCE
9. Prediction of putative RBS within the extended linker
10. Translational coupling of ribocomputing devices using synTCE
11. Translational coupling of 3WJ repressor

12. Dual-output transcript with RBS strength variants

13. Dual-output transcript with synTCE variants

14. The implementation of triple-output generator

15. Synthetic single output mediated cascades

16. Optimization of synthetic cascade architectures using ssrA degradation tag

17. Time-course profiles for GFP and mCherry fluorescence of signaling cascade using TetR and Ecf11_987

18. Stoichiometric control of dual-output transcript

19. Implementation of ribocomputing devices with multiple outputs

20. SDS-PAGE results depending on the presence of translational coupling

21. Schematic flow of classification and criteria of GFP localization

22. Precisely controlled localization via strategic positioning of signal peptides

23. Time-course profiles for cell density of probiotic E. coli Nissle1917 with signal responsive lysis circuit

24. Evaluation of transcription levels of synTCE

25. Enhanced coupling efficiency of synTCE via engineered RBS

26. Characterization of synTCE architecture

27. Hypothesis for the reduced GFP fluorescence of NAND AC gate

**Supplementary Table**

1. General construct architecture

2. Sequence information of riboregulators

3. Sequence information of promoters, terminators, and proteins

4. Sequence information of characterization of synthetic translational coupling elements (synTCEs)

5. Representative examples of plasmid full sequences

**References**

# Supplementary Method

### Design of synthetic translational coupling element

To computationally design synTCE variants using custom python script, we exploited NUPACK 4.0 and Biopython module. To design RNA sequence, we used the NUPACK design script. In addition, to check in-frame stop codon, we utilized sequence translation function of Biopython. The LK sequence in the following code is a linker sequence, which was used to show translation of designed sequence.

**Source code**

Design of synthetic translational coupling element

```
# Import NUPACK Python module
from nupack import *
from Bio.Seq import Seq

# Sequence translation
def sequence_translation(seq):

    from Bio.Seq import Seq

    bioseq_translation=Seq(seq).translate()
    bioseq_translation=str(bioseq_translation)
    seq_list=[]

    for i in range(0, len(bioseq_translation)):
        seq_list.append(f'-(1)-')

    seq_translation=''.join(seq_list)

    return seq_translation

# Structure prediction
def structure(seq):
    seq=str(seq)
    m=mfe(strands=[seq], model=my_model)
    return str(m[0].structure)
```

```
  # Specify model
  my_model = Model(material='rna06', ensemble='stacking', celsius=37, sodium=1.0, magnesium=0.0)

  # Specify Domains
  LK = Domain('aacctggcggcagcgcaaaag', name='LK')
  N1 = Domain('N1', name = 'N1') # For adjusting protein coding frame.
  stem = Domain('NNNNNN', name='N6')
  RBS = Domain('AGAGGAGA', name='RBS')
  overlapped_codon=Domain('URAUG', name = 'overlapped_codon')

  # Define Constraints
  my_soft_constraints=[Pattern(['A4', 'C4', 'G4', 'U4', 'M6', 'K6', 'W6', 'S6', 'R6', 'Y6'])]

  # Specify Strands
  STRAND = TargetStrand([LK,N1,stem,RBS,~stem,overlapped_codon], name='STRAND')

  # Specify Complexes
  COMPLEX = TargetComplex([STRAND], '......................((((((........)))))).....', name= 'COMPLEX')

  # Set a stop condition of 1% and a seed for random number generation to get a reproducible result for this demo
  my_options = DesignOptions(f_stop=0.01, seed=0)

  # Define and run the complex design job
  my_design=complex_design(complexes=[COMPLEX], soft_constraints=my_soft_constraints, options=my_options, model=my_model)
  my_result = my_design.run(trials=1)[0]

  Designed_sequence=str(my_result.to_analysis(STRAND))

  print(Designed_sequence)
  print(sequence_translation(Designed_sequence))
  print(structure(Designed_sequence))
```

**Microplate reader analysis**

Microplate reader is used to confirm the general expression pattern of fluorescence reporters before conducting FACS analysis. Cell cultures of 200 µL were added per well on a 96-well Black Plate (Cat #. 33396, SPL, Gyeonggi-do, Korea) after 0.1 mM IPTG induction or 0.2% (w/w) arabinose induction. GFP fluorescence (excitation: 479 nm, emission: 520 nm), mCherry fluorescence (excitation: 587 nm, emission: 610 nm) and OD600 were measured in a Synergy H1 microplate reader (BioTek Gen5, Santa Clara, CA, USA) running Gen5 3.08 software. GFP and mCherry fluorescence levels were normalized as follows: fluorescence of LB blank was subtracted for background normalization, and the measured fluorescence value was divided by the OD600 value in the corresponding well. The number of biological replicates was three.

For the signaling cascade using TetR in Supplementary Figure S17, overnight cultures were diluted 1/100-fold into 200 µL of fresh medium of 96-well Black Plate and moved to Synergy H1 microplate reader (807 cycles per minute (cpm), 37˚C), then time course fluorescence measurement was conducted. After 3 h, cell cultures were induced with 0.01% (w/w) arabinose and returned to the microplate reader (807 cpm, 37˚C). Time course fluorescence measurement was conducted for an additional 6 h.

**SDS-PAGE**

Hexa-histidine (His6) tag was attached to the C-terminal of GFP positive control, toehold switch, translationally coupled toehold switch and decoupled toehold switch used in Supplementary Figure S20. The constructs were expressed via T7 RNA polymerase in *E. coli* BL21 DE3 strain. All the experimental procedures were identical, except that 1 mM IPTG was used as an inducer. Cell pellets were collected by centrifugation for 1.5 min at 13,000 rpm and resuspended in the volume of lysis buffer (50 mM Tris-HCl (pH 7.8), 200 mM NaCl, 5 mM DTT, 5% (v/v) Glycerol; Sigma Aldrich, Promega). For the high concentration of His6 tagged products, cells were concentrated by a factor of 5. To prevent protease activity in cell lysate, final concentration of 1 mM PMSF (Gold biotechnology) was treated. After 10 min cooling in ice condition, the lysates were briefly sonicated by 2 cycles of 15 % amplitude short burst for 30 sec followed by intervals of 30 sec for cooling using Branson Digital Sonifier SFX 550 (Emerson, Danbury, CT, USA). After sonication, cell debris is removed by centrifugation at 4℃

for 30 min at 13,000 rpm. His6-tagged GFPs were then purified with Ni-NTA spin kit (Qiagen, Hilden, Germany). After purification, collected His6-tagged GFPs were separated in TGX Stain-Free™ FastCast™ Acrylamide Kit, 15% (BIORAD, Hercules, CA, USA) for 1 h with 200 V and then visualized with Azure 260 imager (Azure biosystems, Dublin, CA, USA).

**Bacterial cell classification for GFP localization**

To establish a baseline for the GFP localization status of a particular bacterium, 30 bacteria with and without synTCE construct were collected as reference samples. Consequently, a comparison between a level of 0.65 at the maximum fluorescence intensity across the cell body and mean intensity of cell position (0.4 to 0.6) was able to effectively distinguish between localized and non-localized bacteria (Supplementary Figure S21). The raw data of intensity plots of bacteria with and without translational coupling was collected from the cell collection. In the DsbA signal peptide without translational coupling, bacteria with non-localized and localized GFP are mixed in proximity. Specifically, clustered cells were sometimes misclassified as a single cell. These were excluded from the analysis using an in-house python script and further verified by visual inspection.

**RT-qPCR analysis**

For the preparation of RNA samples, 1 mL of cell culture was collected for RNA extraction after the IPTG induction. Total RNA was extracted using RiboEX (GeneAll, Seoul, Korea) in DNase/ RNase-free condition. cDNA was synthesized with 1 µg of total RNA as a template using GoScriptTM Reverse Transcriptase with random primers (Promega, Madison, WI, USA) with the following conditions: Anneal primer - 25 °C for 5 min, Extension - 42 °C for 60 min, Inactivation – 70 °C for 15 min. Concentration of total RNA and cDNA was measured with a Synergy H1 microplate reader. The 1 µg of cDNA was diluted 1/20-fold, then used for the quantitative PCR using ORATM qPCR Green ROX L MIX (highQu, Kraichtal, Germany) in Stratagene Mx3000P (Agilent Technologies, Santa Clara, CA, USA) with the following conditions: Initial denaturation - 95 °C for 2 min, Denaturation – 95 °C for 5 sec, Annealing/Extension – 60 °C for 20 sec. Amplicon length for target and normalizer gene is 107 bp and 340 bp, respectively. The mixture without template cDNA was used as a non-template control, and no unspecific Cq value was detected on non-template control. The number of replicates was three for each condition. All measurement s were followed by melting curve analysis. Ct values were analyzed using MxPRO software (Agilent Technologies, Santa Clara, CA, USA).

ΔΔCt method is used to investigate the difference in the transcription level between the toehold switch with or without synTCE1. For ΔΔCt analysis, the GFP gene is selected as a target gene, and the 16s ribosomal RNA gene is selected as a normalizer gene. The normalized expression (ΔCt) were calculated as follows: ΔCt = Ct(GFP)-Ct(16s RNA). Primer sequences used for the RT-qPCR analysis is described as followed: GFP forward primer (5′-TGGAAGCGTTCAACTAGCAG-3′), GFP reverse primer (5′- TCGAAAGGGCAGATTGTGTG-3′), 16s rRNA forward primer (5′-GTTAATACCTTTGCTCATTGA-3′), and 16s rRNA reverse primer (5′-ACCAGGGTATCTAATCCTGTT-3′). qPCR primers for GFP gene were designed with primer3 tool (1), and qPCR primers for 16s rRNA were imported from previous research (2). The specificity of primer pairs was assessed with Primer-BLAST, and no off-target matches were identified in NCBI Transcript Reference Sequences of Escherichia coli. DNA oligonucleotides of qPCR primers were purchased from Bionics (Seoul, Korea). The measurement of PCR efficiency is also described in Supplementary Figure S24. The sequence information of target gene is described in Supplementary Table S3.

**Detailed explanation of reinitiation inhibition score**

After termination, the ribosome transiently roams on the mRNA and subsequently either finds the nearest start codon to reinitiate translation or disassembles for recycling (3,4). Previous research has shown that the local mRNA secondary structure after the post-termination ribosome influences this scanning mode (5). To evaluate how mRNA structure inhibits ribosomal reinitiation, we developed a scoring system. In our analysis, we assumed that the post-termination ribosomes translocate on the mRNA in a nucleotide-by-nucleotide manner until the ribosomal P-site is positioned at a start codon, allowing translation to reinitiate. We calculated the extent of base pairing for each nucleotide to assess how much each nucleotide inhibits ribosome translocation.

To determine the range of ribosomal translocation, we set the ribosomal footprint from the P-site to the mRNA entry site as 13 nucleotides (Supplementary Figure S7A). Additionally, the mRNA sequence is divided into 40-nucleotide-long windows for RNA structure prediction (5). To evaluate how much each nucleotide interferes with ribosome translocation, we score the

base pairing status of the nucleotide located just after the ribosome's mRNA entry site. For scoring, the 5′ end nucleotide in a window is used, with each base assigned a specific penalty:

$$Penalty\ score\ of\ G, C\ base = -3$$
$$Penalty\ score\ of\ A, U\ base = -2$$

We utilize an equilibrium pair probability matrix to calculate the base pairing contribution of the 5′ end nucleotide in a window (Supplementary Figure S7B). In the matrix, the (n, n) index represents the probability that each nucleotide does not participate in base pairing, so (1 - matrix (n, n)) indicates the pair probability for the $n^{th}$ nucleotide with other nucleotides. Therefore, the translocation penalty of the 5′ end nucleotide in a window is calculated using the following formula:

$$Translocation\ penalty = (Specified\ penalty\ of\ nucleotide) \times (1 - matrix\ (0,0))$$

This calculation of translocation penalty is repeated, and the reinitiation inhibition score is computed by summing each translocation penalty within the translocation range (Supplementary Figure S7C).

## Supplementary Figure

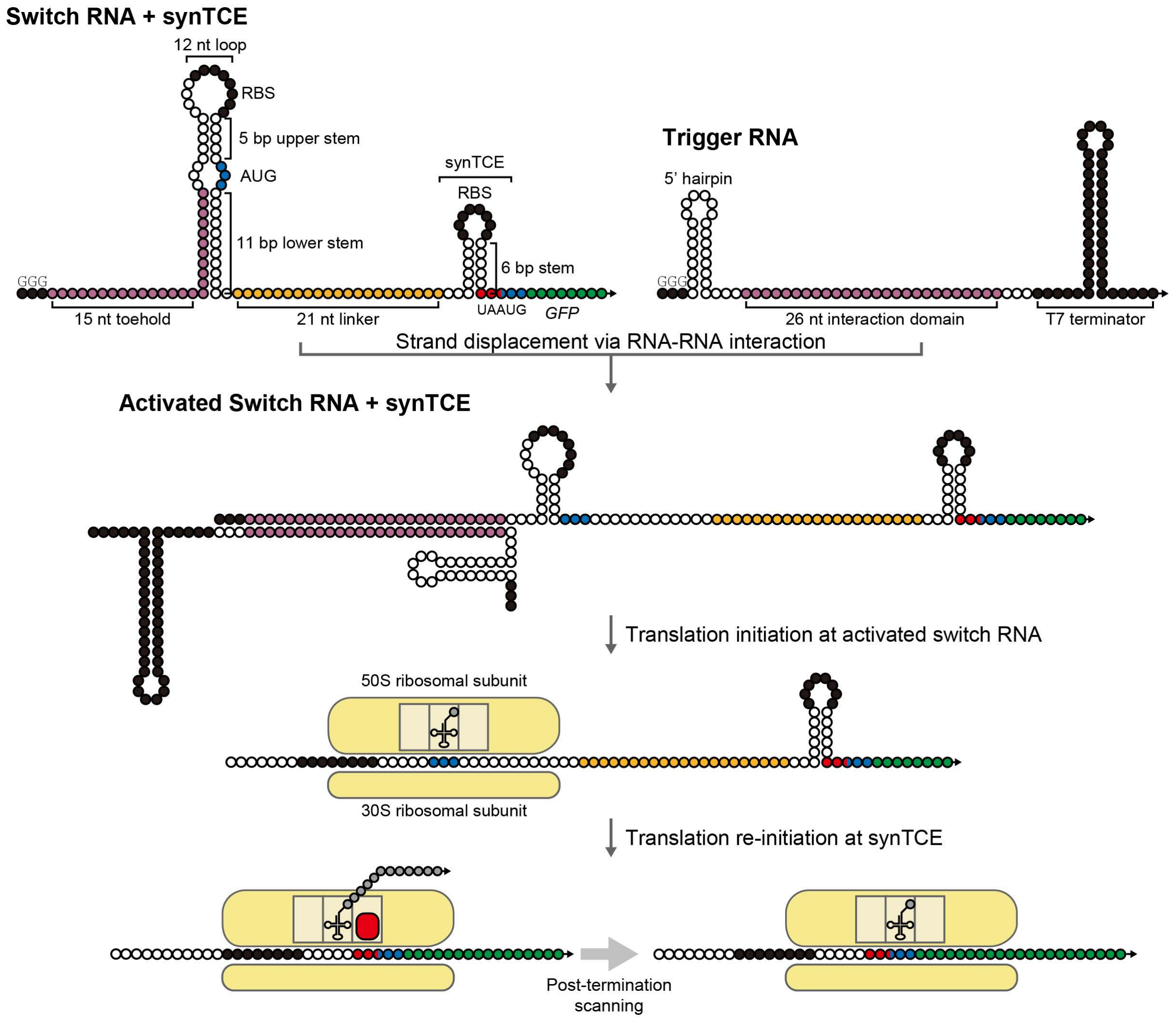


Figure S1. Nucleotide-level schematics of translationally coupled toehold switch. Green and orange bases specify the GFP sequence and the 21-nt linker sequence used, respectively. Black bases mark conserved sequences such as the RBS, and transcriptional terminator. Blue and red bases indicate start codon and stop codon. White bases represent any sequence subject to secondary structure conditions in NUPACK. Programmed hybridization domains between different strands are specified by purple color. The strand displacement via interaction between switch and trigger RNA leads the exposure of RBS sequence. Translation initiation at the first RBS of activated switch RNA then proceeds until faces the stop codon at synTCE domain. Translation reinitiation is conducted via post-termination scanning of ribosome at synTCE domain.

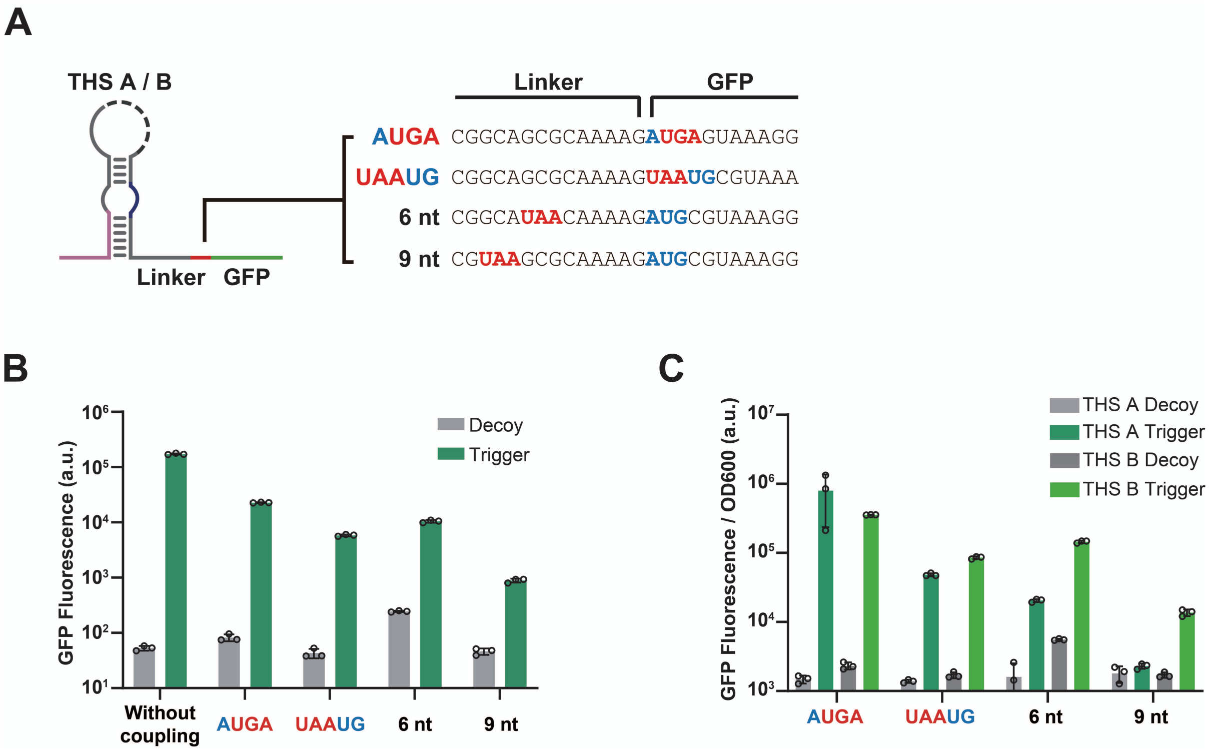


Figure S2. Translational coupling through adjacent ORF configuration. (A) Schematic represents the location of stop codon in the sequence. Red and blue letters mark stop and start codon, respectively. 6 and 9 nt indicate the intergenic distance between the start and stop codon. (B) Flow cytometry GFP fluorescence was measured on translational coupling elements with adjacent ORF configurations using toehold switch D variant. (C) Comparison of coupling efficiency between toehold switch variants. Measurements were taken 3 h 30 min after induction with 0.1 mM IPTG. Dot points specify the individual data, bars represent mean ± s.d. of n = 3 biological replicates.

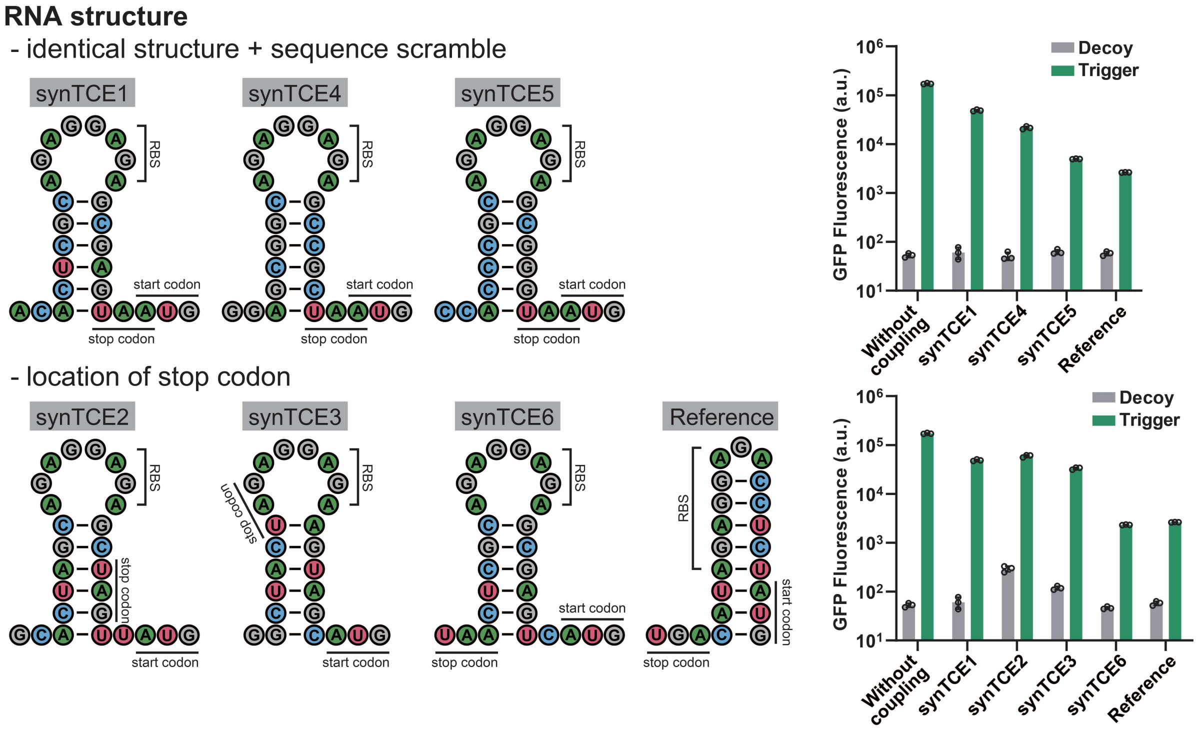


Figure S3. SynTCE variants showing different performance. Schematic displays the MFE structure of synTCEs. SynTCE variants have different stem sequence or rearranged stop codon positions. Each circle indicates the nucleotide sequence identity. Translational coupling element from a previous study was used as a reference (4). Flow cytometry GFP fluorescence was measured 3 h 30 min after induction with 0.1 mM IPTG. Dot points specify the individual data, bars represent mean ± s.d. of n = 3 biological replicates.

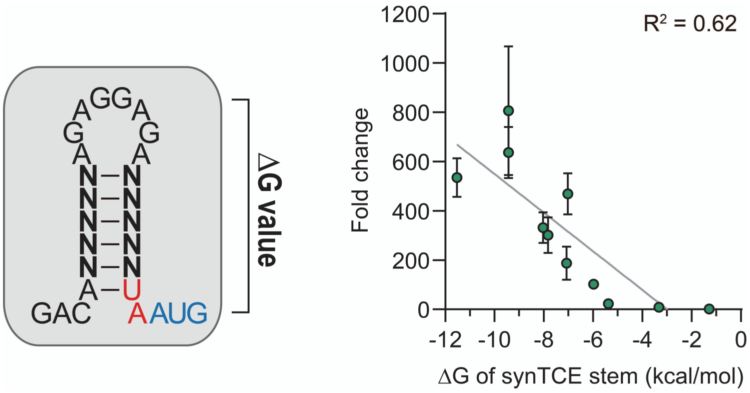


Figure S4. Correlation between ΔG of stem region of synTCE and GFP fluorescence. ON/OFF fold change and ΔG of stem base variants used in figure 2B were used to analysis. Fold change is the ratio of GFP fluorescence level for the ON and OFF states. The relative errors for the ON and OFF states are from the s.d. of n=3 biologically independent samples. Relative errors for GFP fold reduction were obtained by adding the relative errors of ON and OFF state fluorescence measurements in quadrature. Bars represent mean ± relative errors.

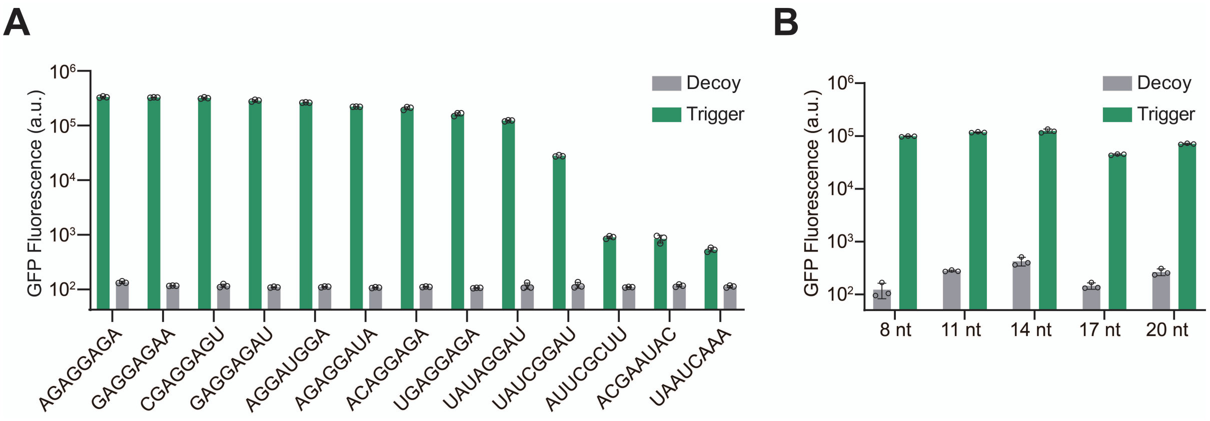


Figure S5. The result of loop modification of synTCE. Flow cytometry GFP fluorescence of randomized 8 nucleotides of loop (A) and the varying length of loop (B). Measurements were taken 3 h 30 min after induction with 0.1 mM IPTG. Dot points specify the individual data, bars represent mean ± s.d. of n=3 biological replicates.

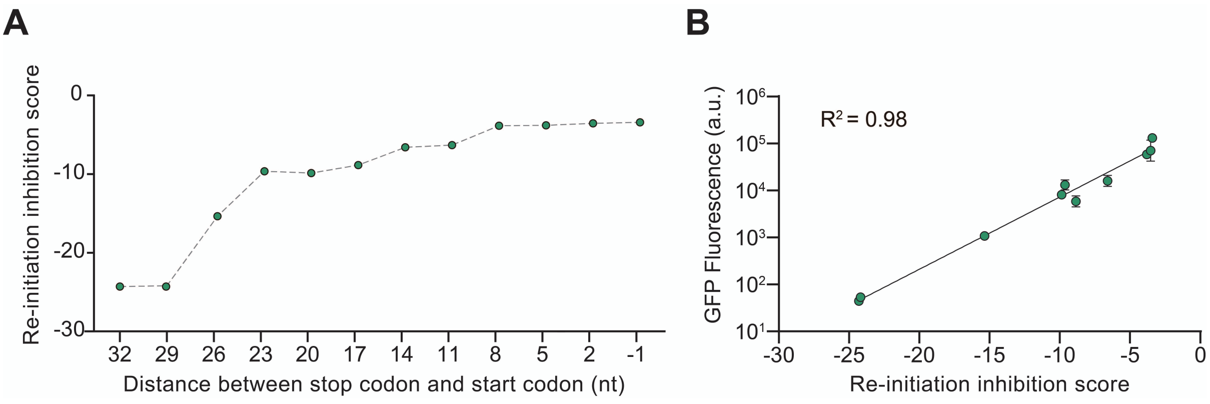


Figure S6. The result of reinitiation inhibition score in the stop codon tiling assay. (A) Reinitiation inhibition score depending on the spacing length between stop codon and start codon. The reinitiation inhibition score represents how much post-termination ribosome overcome energy penalty endowed by local RNA structure until the ribosome finds start codon. (B) The linear regression between reinitiation inhibition score and GFP fluorescence. In this analysis, the constructs containing the spacing 8 and 11 are excluded due to the modification of RBS sequence. Bars represent mean ± s.d. of n=3 biological replicates. See also Supplementary Method and Supplementary Figure S7.

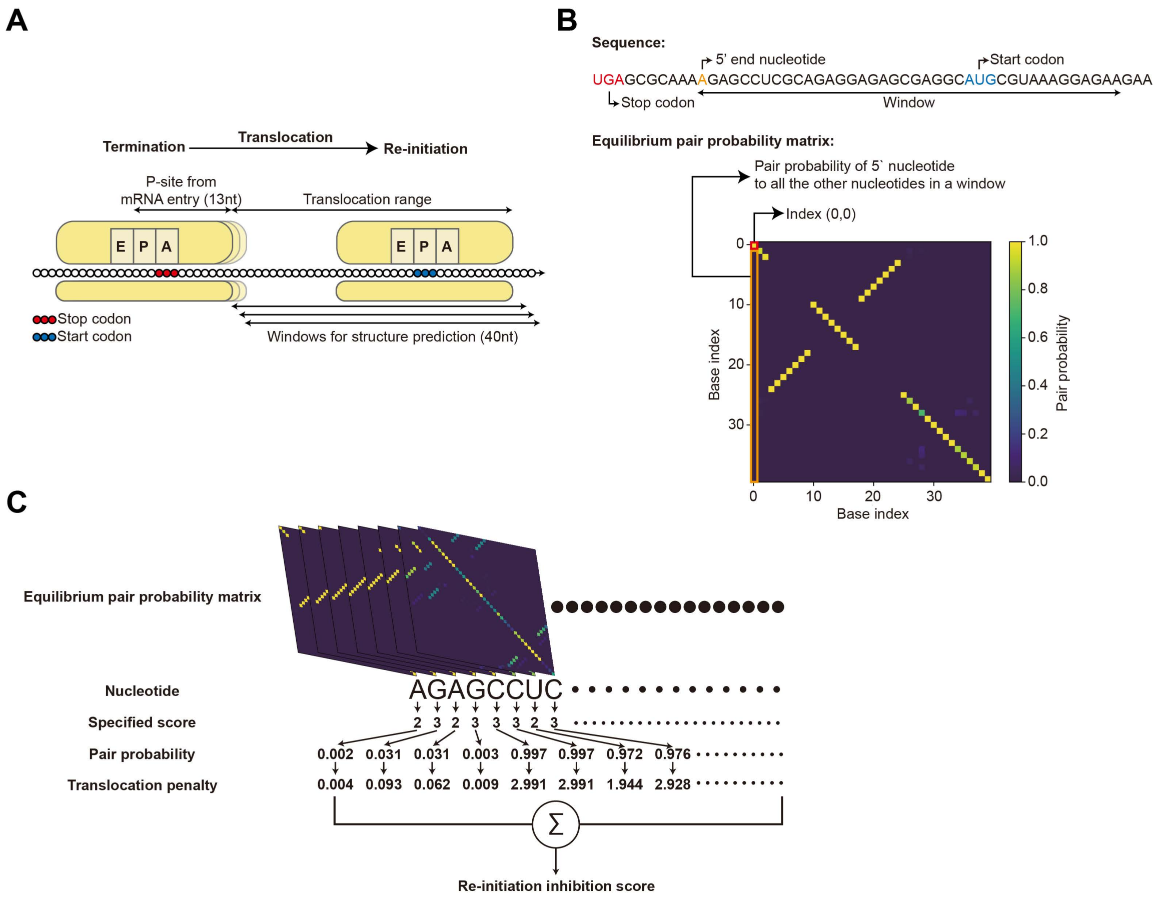


Figure S7. Detailed schematics for the calculation of reinitiation inhibition score. (A) Nucleotide-level schematic to calculate the reinitiation inhibition score. The distance between the mRNA entry site and the P-site of the ribosome is used to determine the translocation range. The starting position of the windows is determined by ribosomal translocation. (B) Example calculation of pair probability for the 5′ end nucleotide. To calculate the pair probability, RNA structure prediction is performed using the sequence within a window. In an equilibrium pair probability matrix, the orange box represents the pair probability of the 5′ end nucleotide, which is computed by using the value from the red box. (C) Overview of the calculation of reinitiation inhibition score. The pair probability of each nucleotide is multiplied by specified score, resulting in a translocation penalty. This process is repeated across the translocation range, and the penalty values are summed to compute the reinitiation inhibition score.

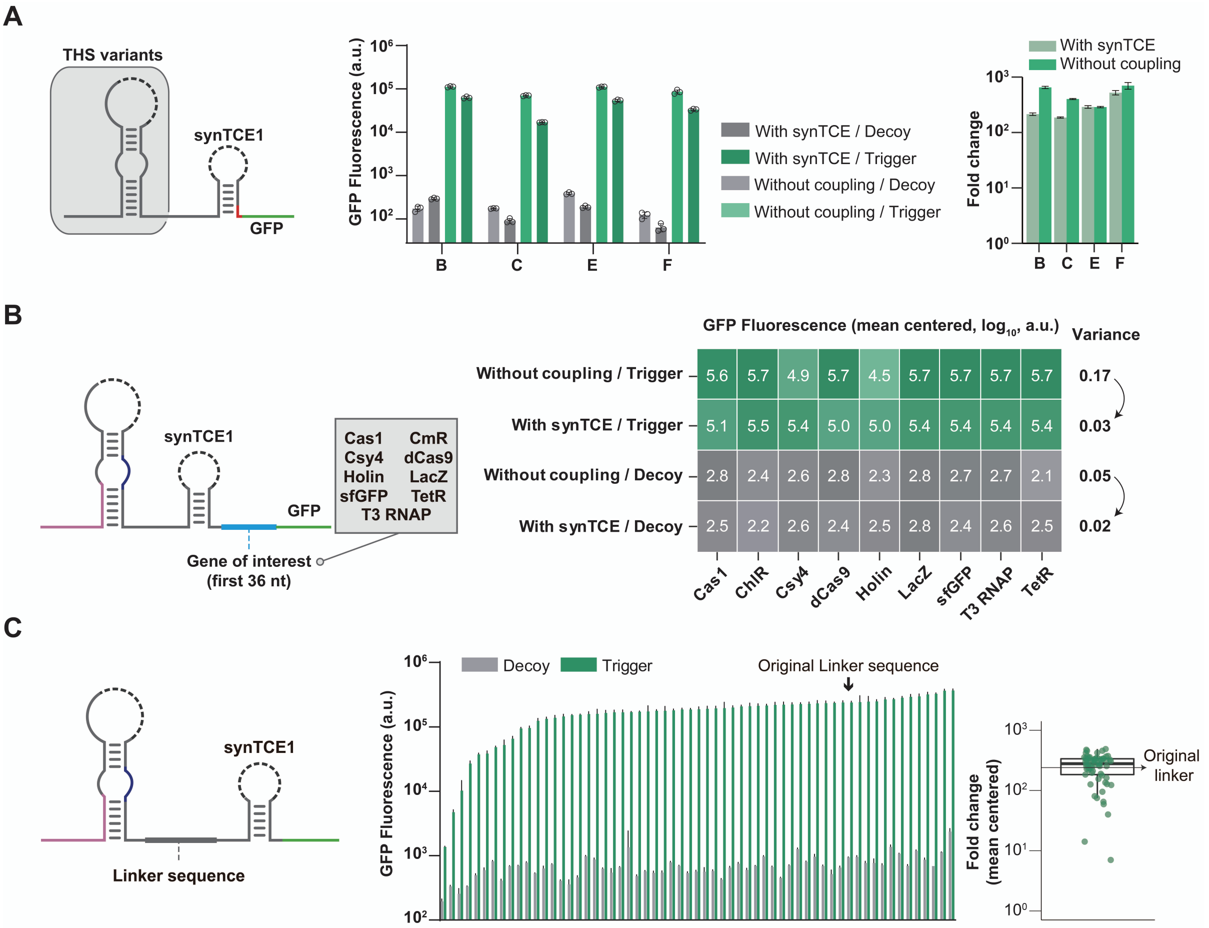


Figure S8. The investigation of surrounding context of synTCE. (A) Context 1: Toehold switch variants. (B) Context 2: Different gene fragments downstream of synTCE. Translationally coupled toehold switch with first 36 nucleotides of target GOIs, including *Cas1*, *dCas9*, *T3 RNAP*, Chloramphenicol resistance (*CmR*), *Csy4*, *Holin*, *LacZ*, superfolder GFP (*sfGFP*), tetracycline repressor (*TetR*) gene. Compared to non-coupling constructs, synTCE fused constructs showed relatively reduced expression variance in both ON and OFF condition. (C) Context 3: Linker sequences. Linker variants were generated by replacing native linker sequence to 21 random nucleotides. Linker variants with stop codons were excluded from the analysis. The measurements of flow cytometry GFP fluorescence were taken 3 h 30 min after induction with 0.1 mM IPTG. Dot points specify the individual data, bars represent mean ± s.d. of n=3 biological replicates. Toehold switch variants used in this figure was described in Supplementary Table S2.

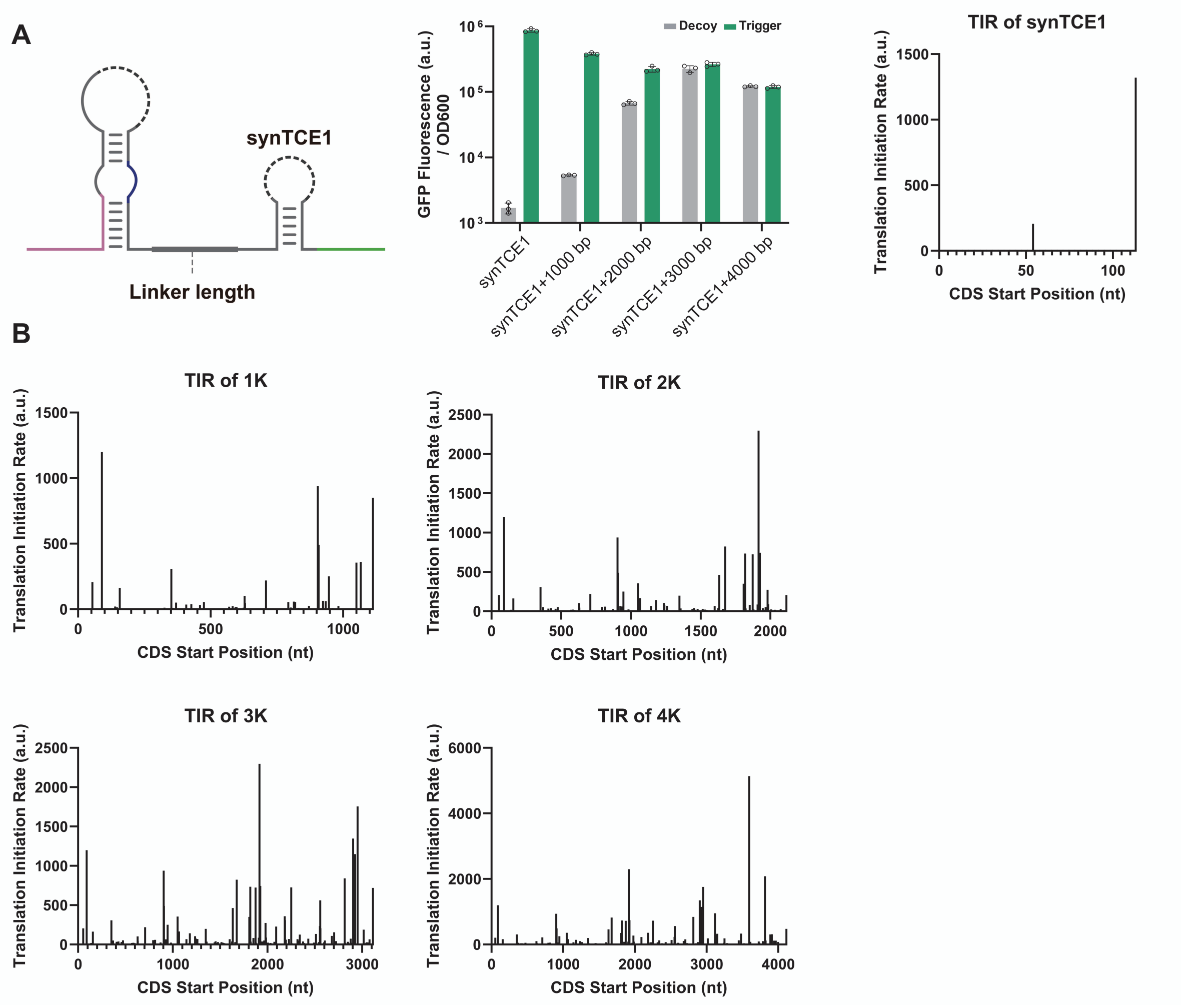


Figure S9. Prediction of putative RBS within the extended linker. (A) Schematic and GFP fluorescence measurement of synTCE coupled toehold switch with extended linkers. The measurements were taken 3 h 30 min after induction with 0.1 mM IPTG. Dot points specify the individual data, bars represent mean ± s.d. of n=3 biological replicates. (B) Predicted translation initiation rate of extended linkers. Each peak indicates the translation initiation rate of given CDS position. The sequence used in the prediction of translation initiation rate was up to the start codon of GFP. The prediction of translation initiation rate was evaluated with RBS calculator using whole sequence of each construct. The linker sequence used in this study was described in Supplementary Table S4.

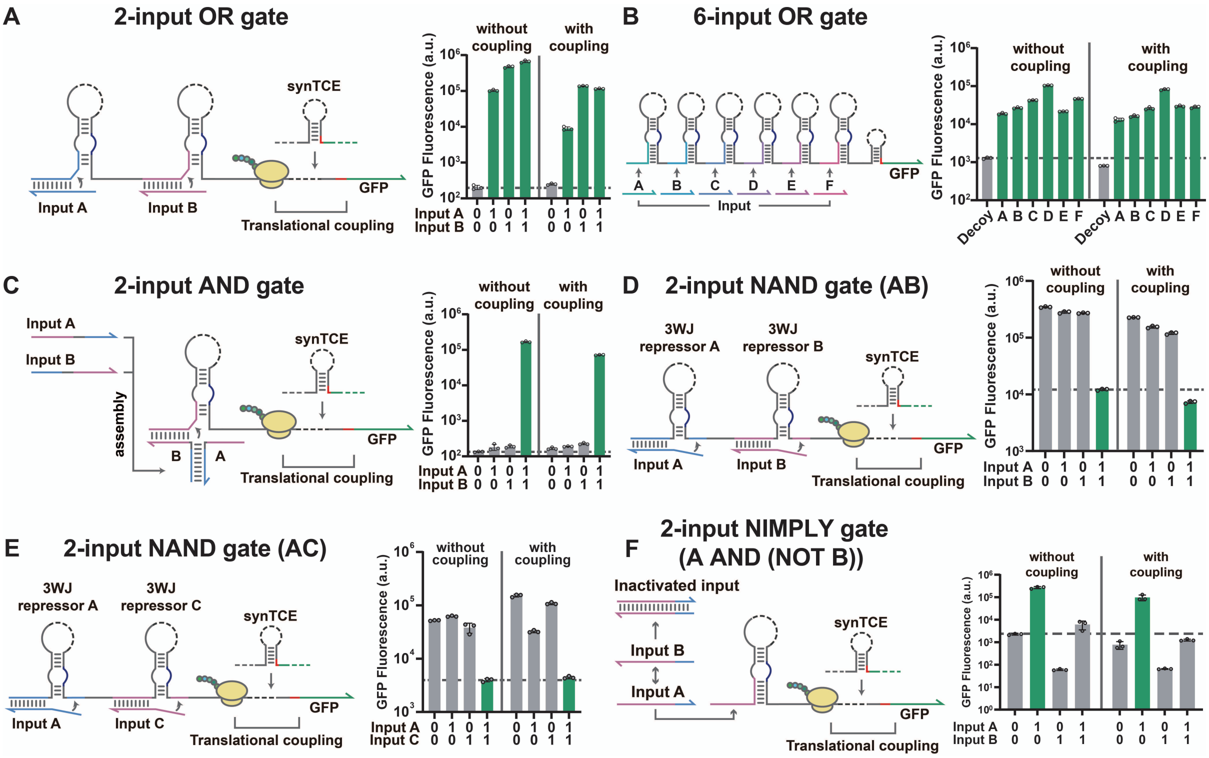


Figure S10. Translational coupling of ribocomputing devices using synTCE. (A-F) Schematic and flow cytometry GFP fluorescence of translationally coupled ribocomputing devices for a two-input OR gate (A), a six-input OR gate (B), a two-input AND gate (C), a two-input NAND (AB) gate (D), a two-input NAND (AC) gate (E), and a two-input NIMPLY gate (F). The combination of inputs is displayed underneath the fluorescence chart. Each input is an individual RNA corresponding to each gate. Measurements were taken 3 h 30 min after induction with 0.1 mM IPTG. Dot points specify the individual data, bars represent mean ± s.d. of n=3 biological replicates.

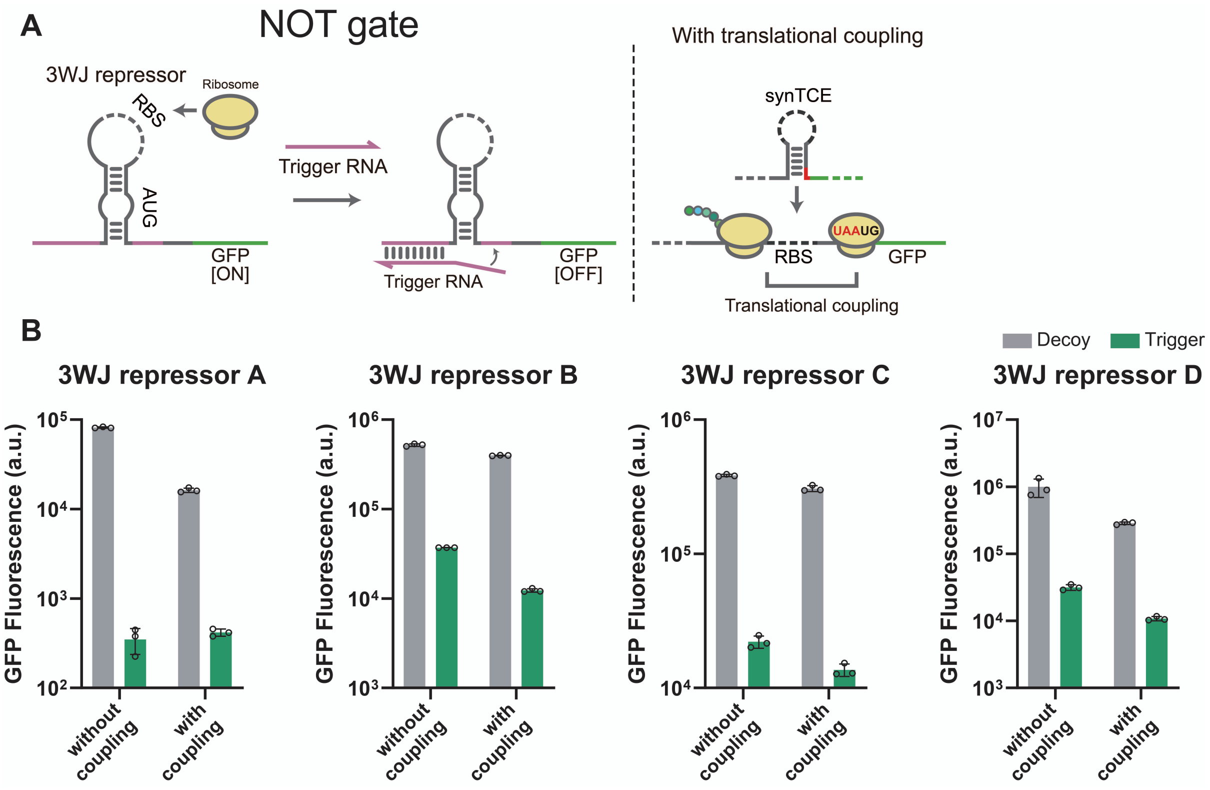


Figure S11. Translational coupling of 3WJ repressor. (A) Schematic indicates the mechanism of NOT gate of 3WJ repressor. 3WJ repressor can produce GFP in its native state, but the formation of three-way junction structure by trigger RNA leads to the transition from ON to OFF state. Translational coupling is implemented with synTCE1. (B) Flow cytometry GFP fluorescence was measured on 3WJ repressors with or without translational coupling elements. The measurements were taken 3 h 30 min after induction with 0.1 mM IPTG. Dot points specify the individual data, bars represent mean ± s.d. of n=3 biological replicates.

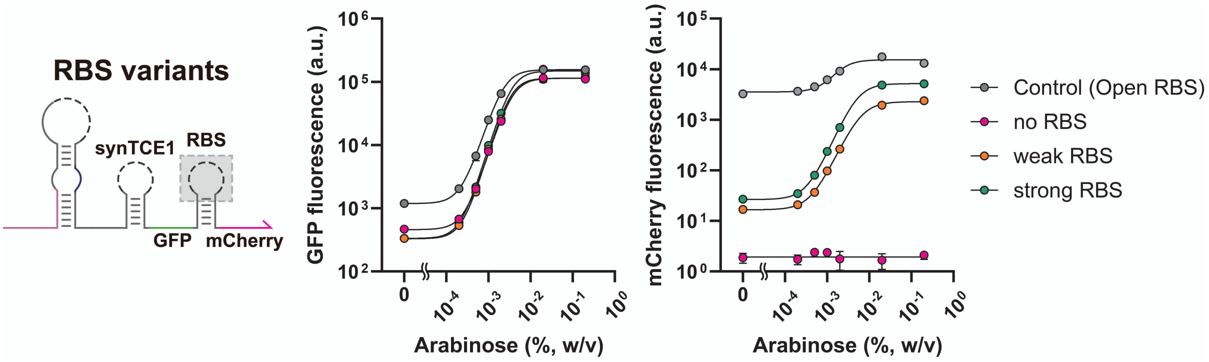


Figure S12. Dual-output transcript with RBS strength variants. The RBS sequence of synTCE between the GFP and mCherry genes is modified. Measurements were taken 5h after induction with 0.1 mM IPTG and 0%, 0.0002%, 0.0005%, 0.001%, 0.002%, 0.02% and 0.2% of arabinose**.** Bars represent mean ± s.d. of n=3 biological replicates. RBS sequence used in this study is described in Supplementary Table S4.

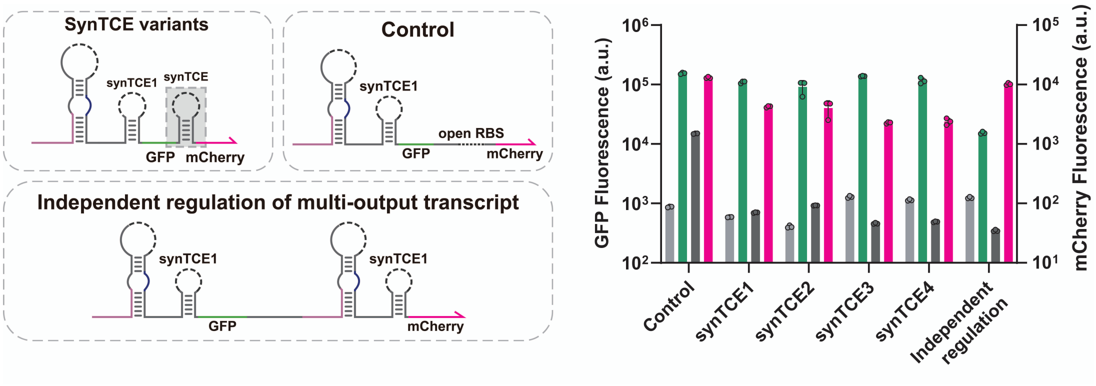


Figure S13. Dual-output transcript with synTCE variants. The synTCE between the GFP and mCherry genes is substituted with different synTCE variants. SynTCE variants were selected from Supplementary Figure S3. As another control, dual-output transcript with multiple switches that control each gene individually was used. For the multi-switch transcript, mCherry expression may increase as it is independent of upstream GFP translation, whereas the GFP expression could be decreased due to the distribution of trigger RNA across the multiple switches. The RBS sequence between the GFP and mCherry genes is modified. Measurements were taken 5 h after induction with 0.1 mM IPTG. Dot points specify the individual data, bars represent mean ± s.d. of n=3 biological replicates.

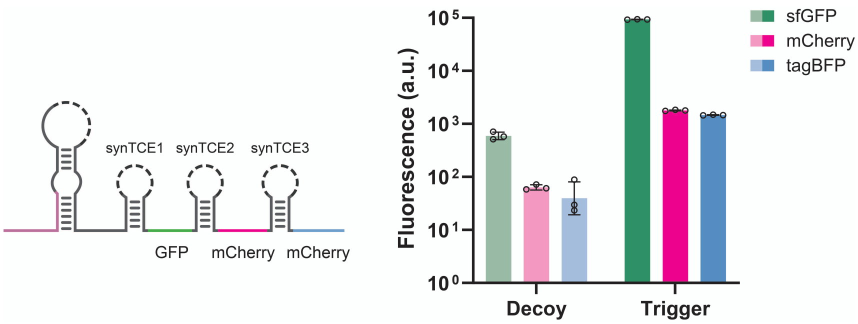


Figure S14. The implementation of triple-output generator. Toehold switch was connected to sfGFP, mCherry and tagBFP reporter via synTCE variants. Flow cytometry GFP, mCherry and tagBFP fluorescence measurements were taken 3 h 30 min after induction with 0.1 mM IPTG. Dot points specify the individual data, bars represent mean ± s.d. of n=3 biological replicates.

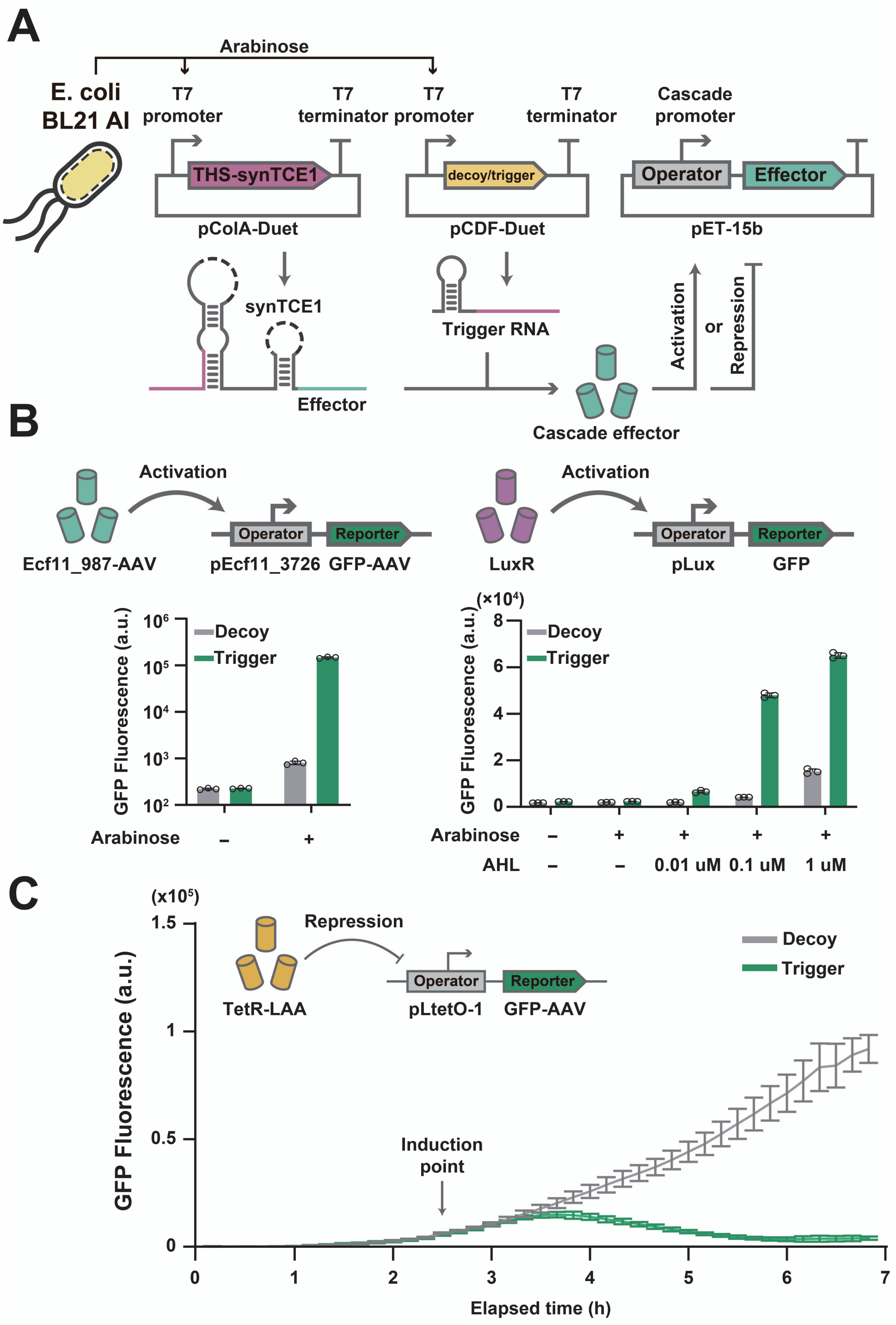


Figure S15. Synthetic single output mediated cascades. (A) Schematic of the implementation of synthetic cascade architectures. Transcription regulators such as Ecf11_987, LuxR and TetR, were used as effector proteins. (B) Flow cytometry GFP fluorescence measurement of synthetic cascade using Ecf11_987 and LuxR protein. Measurements were taken 3 h 30 min after induction of 0.2% arabinose. For LuxR based cascade, the varying concentration of homoserine lactone (AHL) was treated. (C) Time-course profiles for GFP fluorescence of TetR cascade. Induction with 0.2% arabinose was taken after 2 h 30 min of cell incubation. Dot points specify the individual data, bars represent mean ± s.d. of n=3 biological replicates.

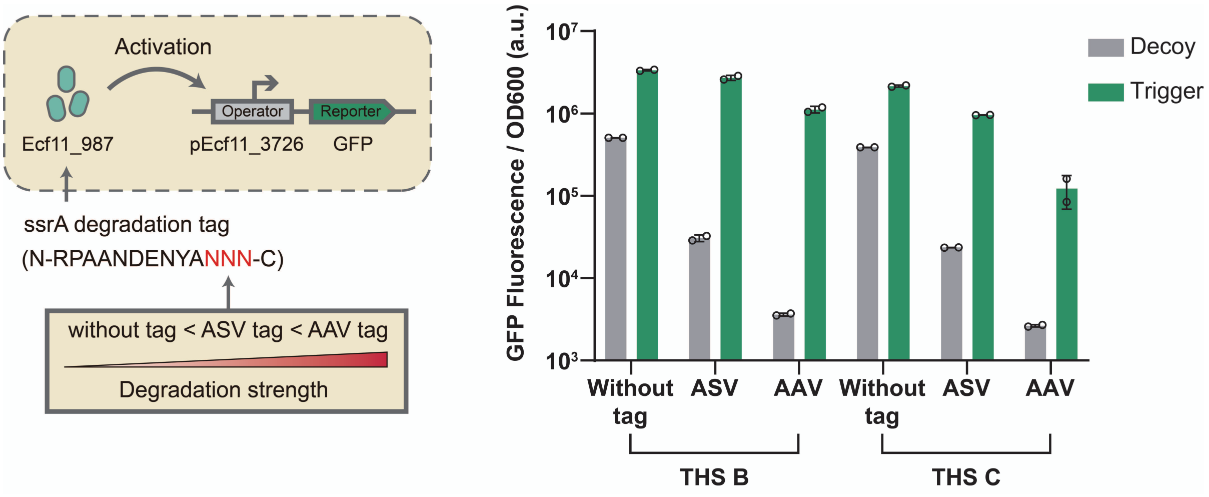


Figure S16. Optimization of synthetic cascade architectures using ssrA degradation tag. Effector proteins were fused with ssrA degradation tag. For representative example, optimization of Ecf11_987 with ssrA tag was displayed. GFP fluorescence measurements were taken 3 h 30 min after induction with 0.2% arabinose. Dot points specify the individual data, bars represent mean ± s.d. of n=2 biological replicates.

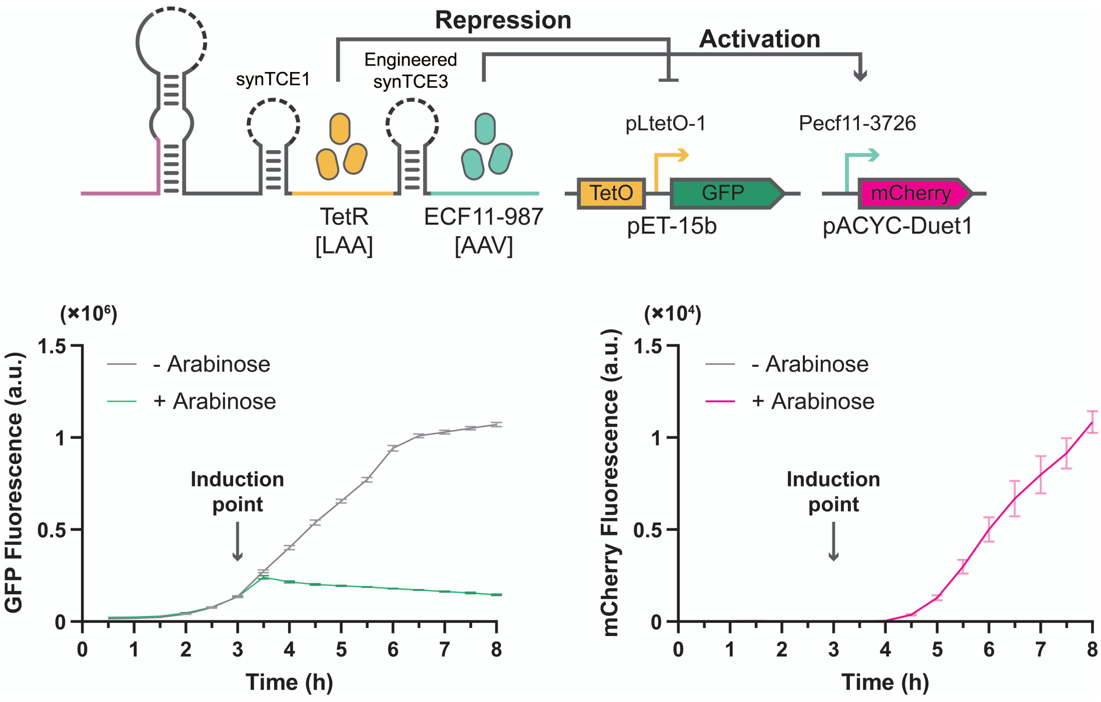


Figure S17. Time-course profiles for GFP and mCherry fluorescence of signaling cascade using TetR and Ecf11_987. With induction, production of GFP was immediately ceased in 30 min. Induction with 0.2% arabinose was taken after 3 h of cell incubation, bars represent mean ± s.d. of n=3 biological replicates.

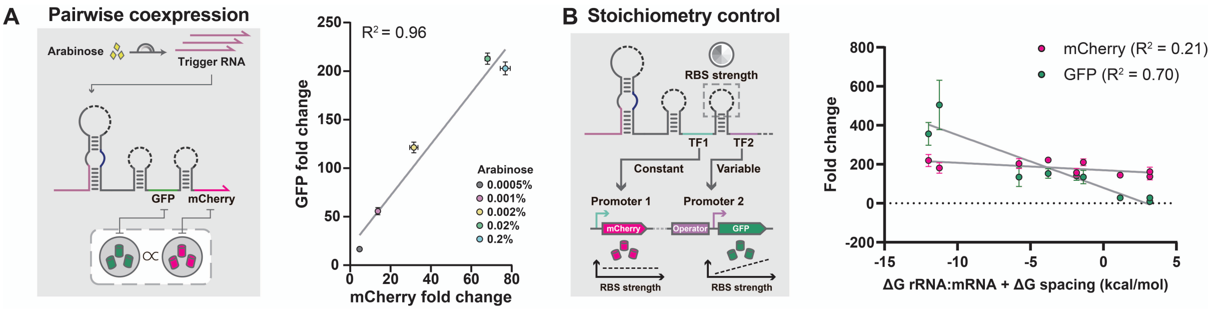


Figure S18. Stoichiometric control of dual-output transcript. (A) Schematic and the linear regression between GFP and mCherry fold change of dual-output transcript. Measurements were taken 5 h after induction with 0.1 mM IPTG and 0%, 0.0002%, 0.0005%, 0.001%, 0.002%, 0.02% and 0.2% of arabinose. (B) Schematic and the linear regression between fold change and RBS strengths. The RBS between Ecf11-987 (TF1) and LuxR (TF2) was modulated with varying strengths evaluated by ΔG rRNA:mRNA + ΔG spacing. Measurements were taken 5 h after induction with 0.1 mM IPTG. Fold change is the ratio of GFP fluorescence level for the ON and OFF states. The relative errors for the ON and OFF states are from the s.d. of n=3 biologically independent samples. Relative errors for GFP fold reduction were obtained by adding the relative errors of ON and OFF state fluorescence measurements in quadrature. Bars represent mean ± s.d. of n=3 biological replicates. See also Supplementary Table 4.

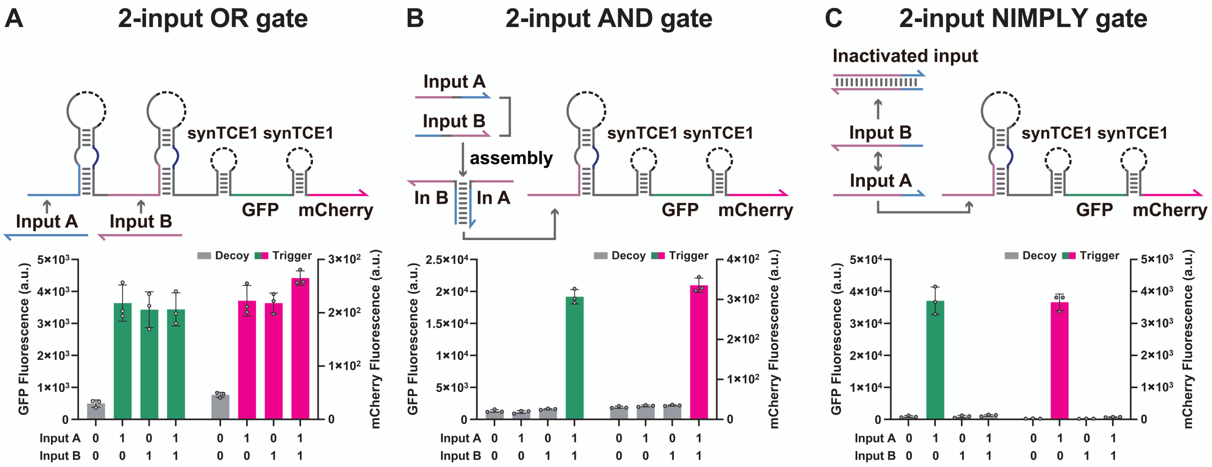


Figure S19. Implementation of ribocomputing devices with multiple outputs. (A-C) Schematic and Flow cytometry fluorescence measurement of 2-input OR gate (A), 2-input AND gate (B), and 2-input NIMPLY gate (C). Each input is an individual RNA corresponding to each gate. Measurements were taken 3 h 30 min after induction with 0.1 mM IPTG. Dot points specify the individual data, bars represent mean ± s.d. of n=3 biological replicates.

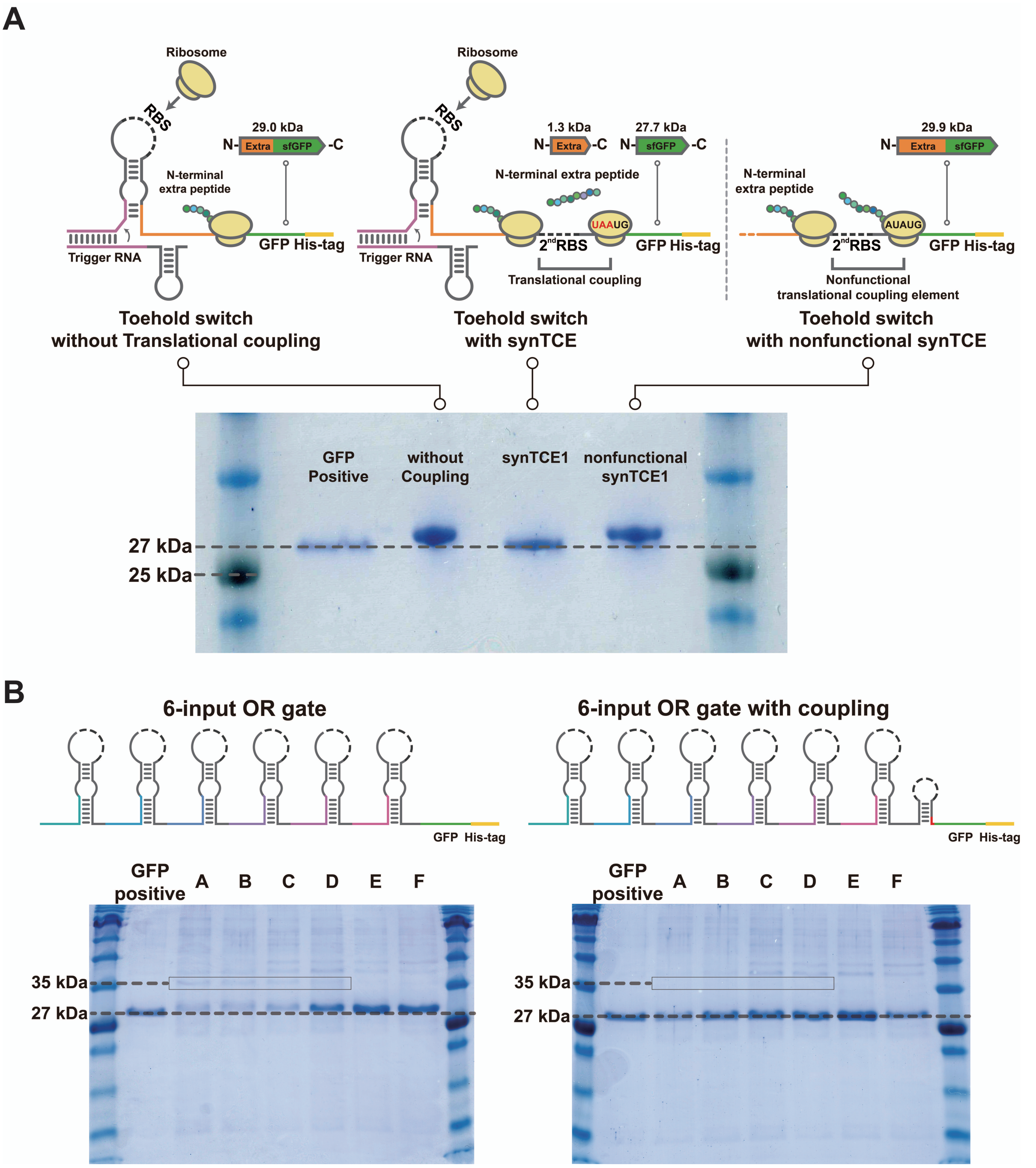


Figure S20. SDS-PAGE results depending on the presence of translational coupling. (A) The SDS-PAGE result of 6X his-tagged GFP expressed from toehold switch with or without translational coupling. As a control, GFP with a molecular weight of 27.7 kDa was used. Due to the presence of additional peptide, GFP produced from toehold switch has 29.0 kDa molecular weight, whereas GFP produced via translational coupling has equivalent weight to the control. Non-functional translational coupling element (deleted stop codon and in-frame configuration with GFP gene) was tested to clarify the action of translational coupling. (B) SDS-PAGE of six-input OR gate with or without translational coupling. All samples were induced with 1 mM IPTG for 3 h 30 min. Samples were lysed via sonication method, then purified with histidine tag purification. See also Supplementary Method.

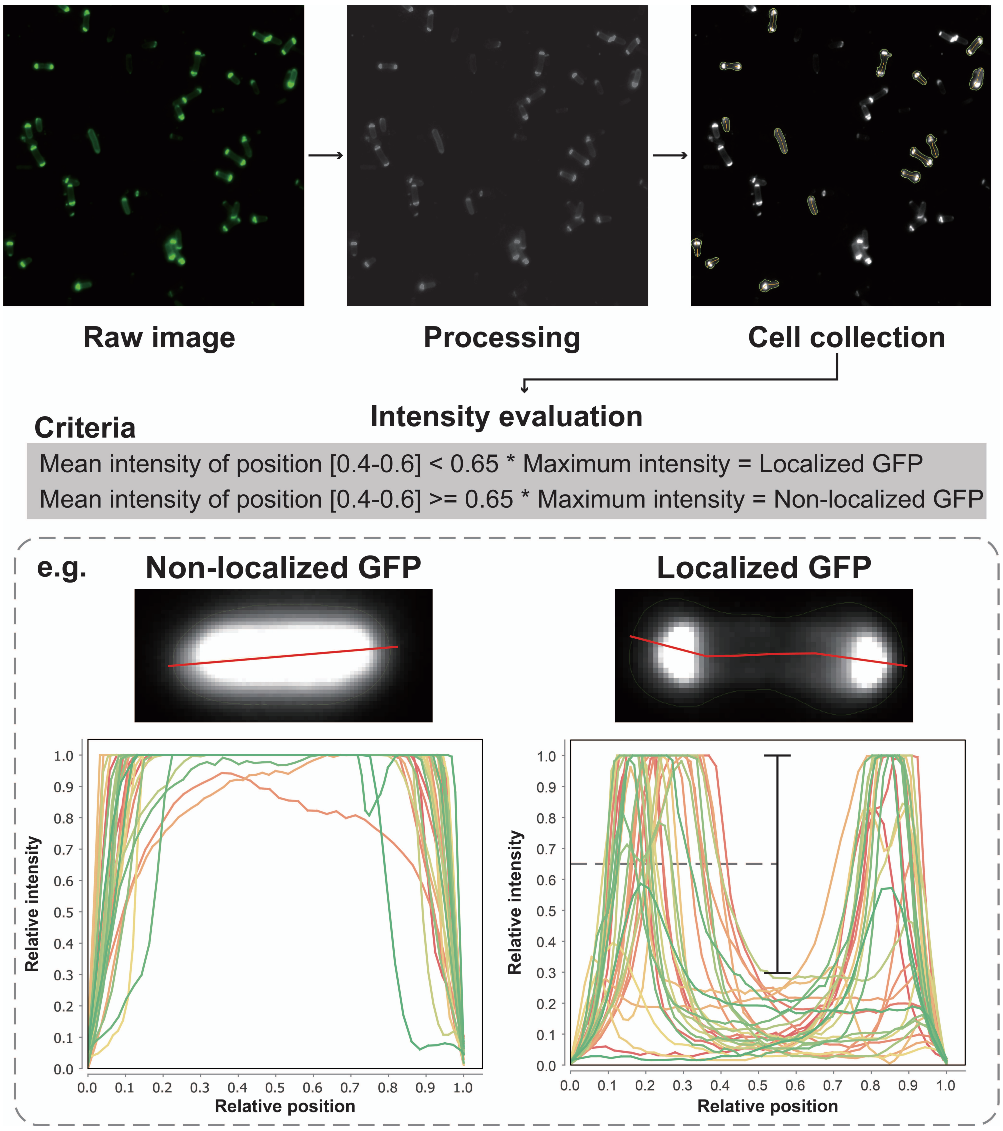


Figure S21. Schematic flow of classification and criteria of GFP localization. Raw sample images were taken 3 h 30 min after induction with 0.1 mM IPTG. Image sampling was performed across multiple different locations in each triplicate cell collection. Raw images were then processed with ImageJ (see Supplementary Method). Cell collection is automatically conducted with MicrobeJ plugin. Classification of GFP localization was performed by processing the collected data using Excel.

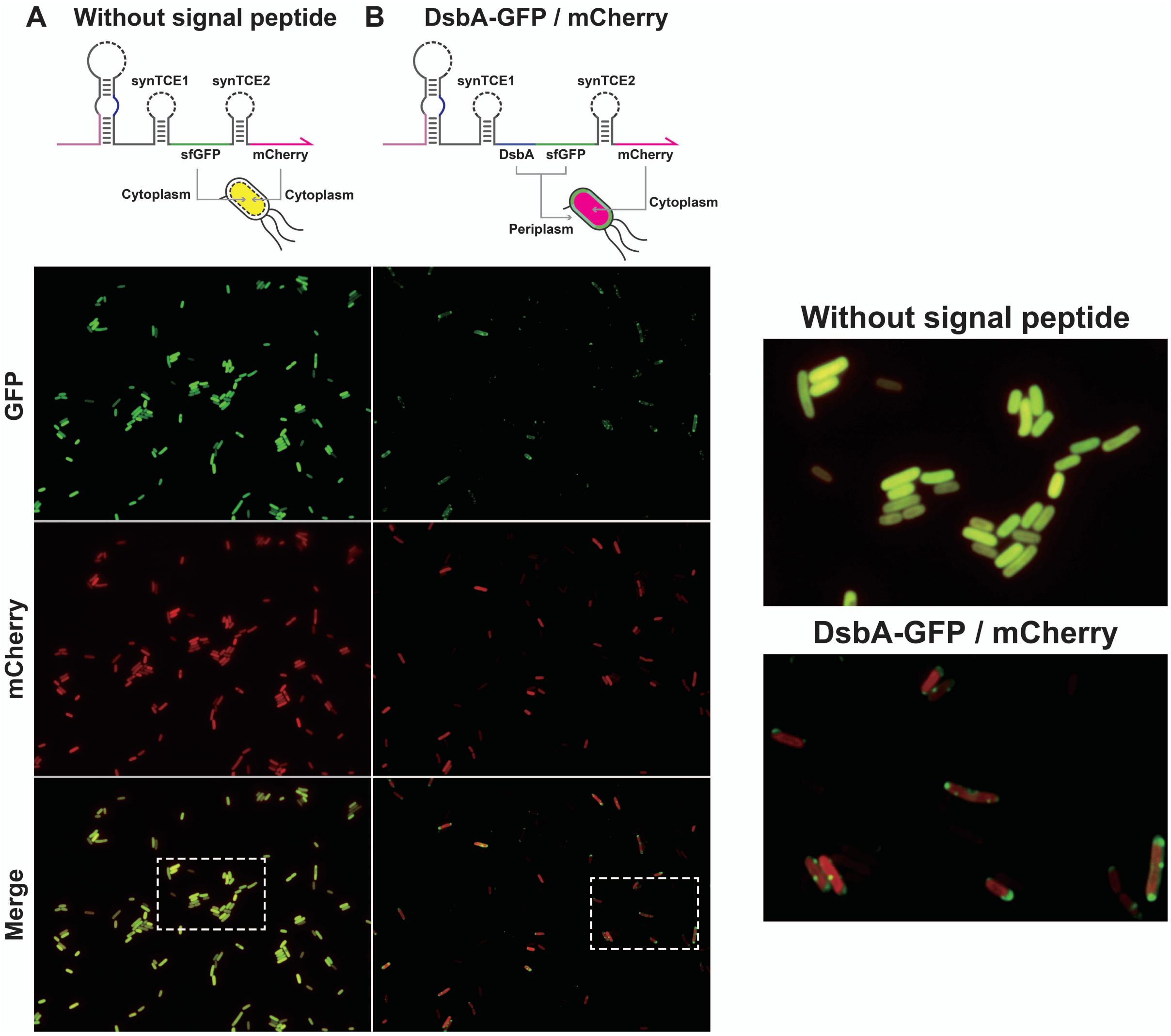


Figure S22. Precisely controlled localization via strategic positioning of signal peptides. (A,B) Schematic and Fluorescence microscopic image of two-output transcript without (A) or with (B) DsbA signal peptide. The DsbA signal peptide is inserted ahead of sfGFP, resulting in the periplasmic localization of sfGFP. The image on the left side shows a magnified view of the area outlined by the white dotted box. Sample images were taken 3 h 30 min after induction with 0.1 mM IPTG. Image sampling was performed across multiple different locations in each triplicate.

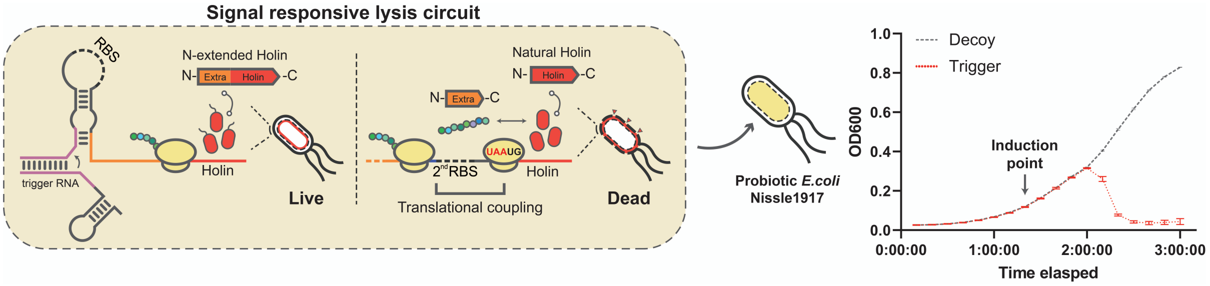


Figure S23. Time-course profiles for cell density of probiotic E. coli Nissle1917 with signal responsive lysis circuit. Signal responsive lysis circuit was transformed into Nissle1917 strain with electroporation. Induction with 0.1 mM IPTG was taken after 1 h 20 min of cell incubation, bars represent mean ± s.d. of n=3 biological replicates.

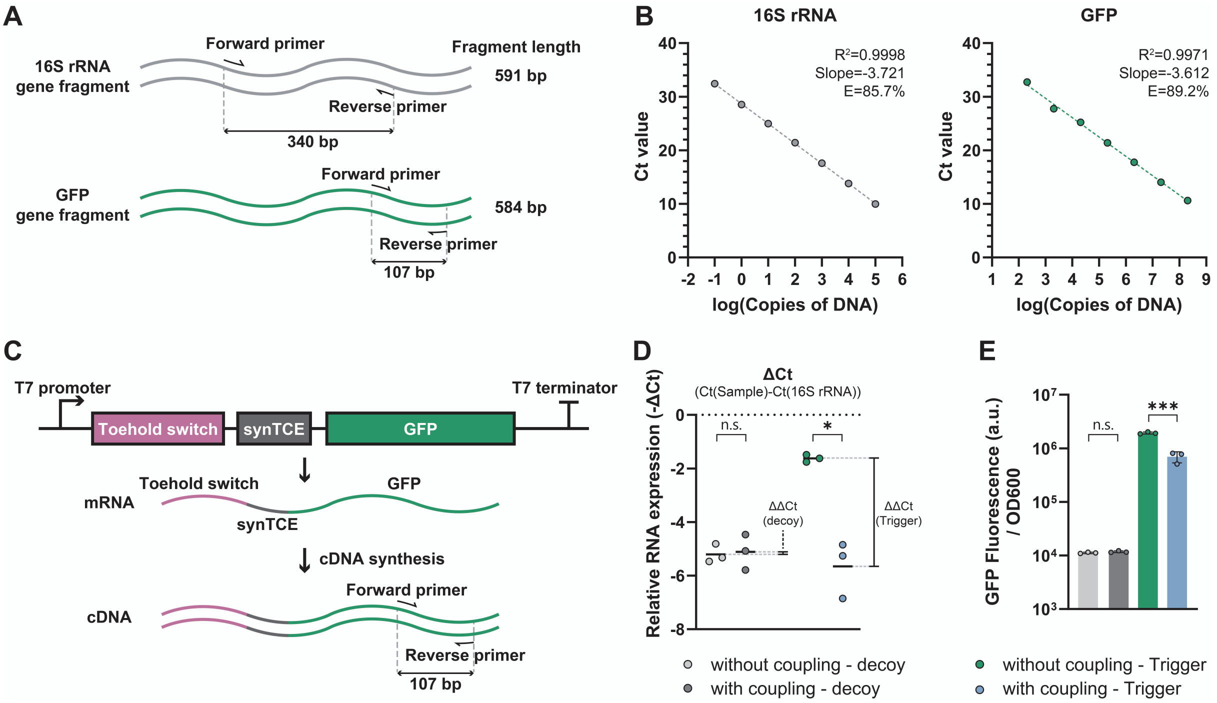


Figure S24. Evaluation of transcription levels of synTCE. (A) Schematic of qPCR assay to measure PCR efficiency. qPCR was performed using serially diluted template samples (16S rRNA and GFP gene fragments) with corresponding primer pairs. (B) Standard curve of 16S rRNA and GFP gene. In the standard curves, the PCR efficiencies were 85.7% and 89.2%, respectively. The similar PCR efficiencies of each gene demonstrate that normalizing the Ct value of GFP by the Ct value of 16S rRNA accurately represents relative gene expression. (C) Schematic of RT-qPCR process. Schematic indicates the preparation of cDNA using reverse transcriptase, the position of primers, and the size of qPCR products. (D) Transcript levels were evaluated according to the presence or absence of synTCE under conditions treated with decoy or trigger RNA. Relative RNA expression is evaluated using 16s rRNA as a control. (E) GFP fluorescence measurement of toehold switch with or without translational coupling. Fluorescence measurements were taken 3 h 30 min after induction with 0.1 mM IPTG. Dot points specify the individual data, bars represent mean of n=3 biological replicates. Two-tailed Student's t-test; ns: not significant, *: $p < 0.05$. ***: $p < 0.001$.

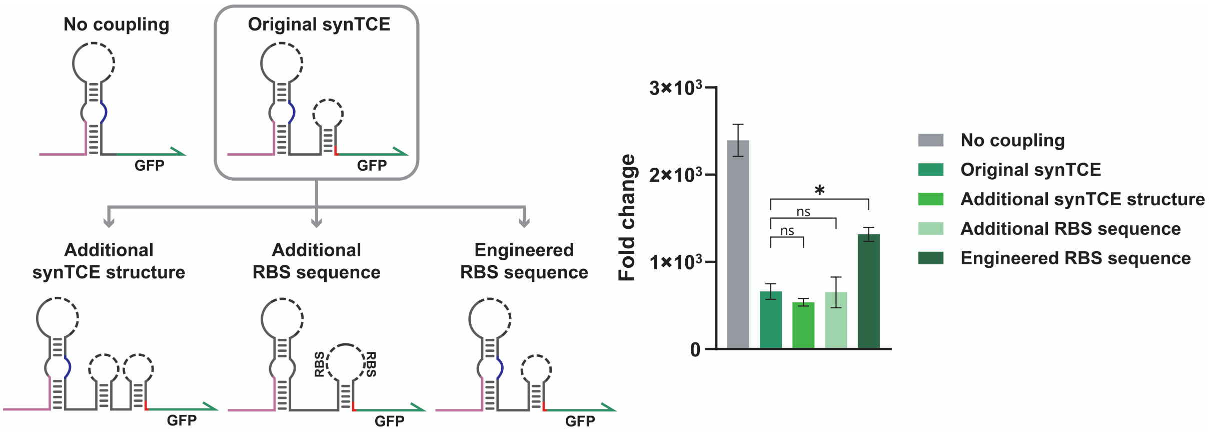


Figure S25. Enhanced coupling efficiency of synTCE via engineered RBS. SynTCE with additional synTCE structure, RBS sequence, and engineered RBS was examined to improve the translational coupling efficiency. The detailed sequences are in Supplementary Table S4. Measurements via flow cytometry were taken 3 h 30 min after induction with 0.1 mM IPTG. Fold change is the ratio of the GFP fluorescence level for the ON and OFF states. Relative errors for GFP fold change were obtained by adding the relative errors of the ON and OFF state fluorescence measurements in quadrature. Bars represent mean ± relative errors of n=3 biological replicates. Two-tailed Student's t-test; ns: not significant, *: $p < 0.05$.

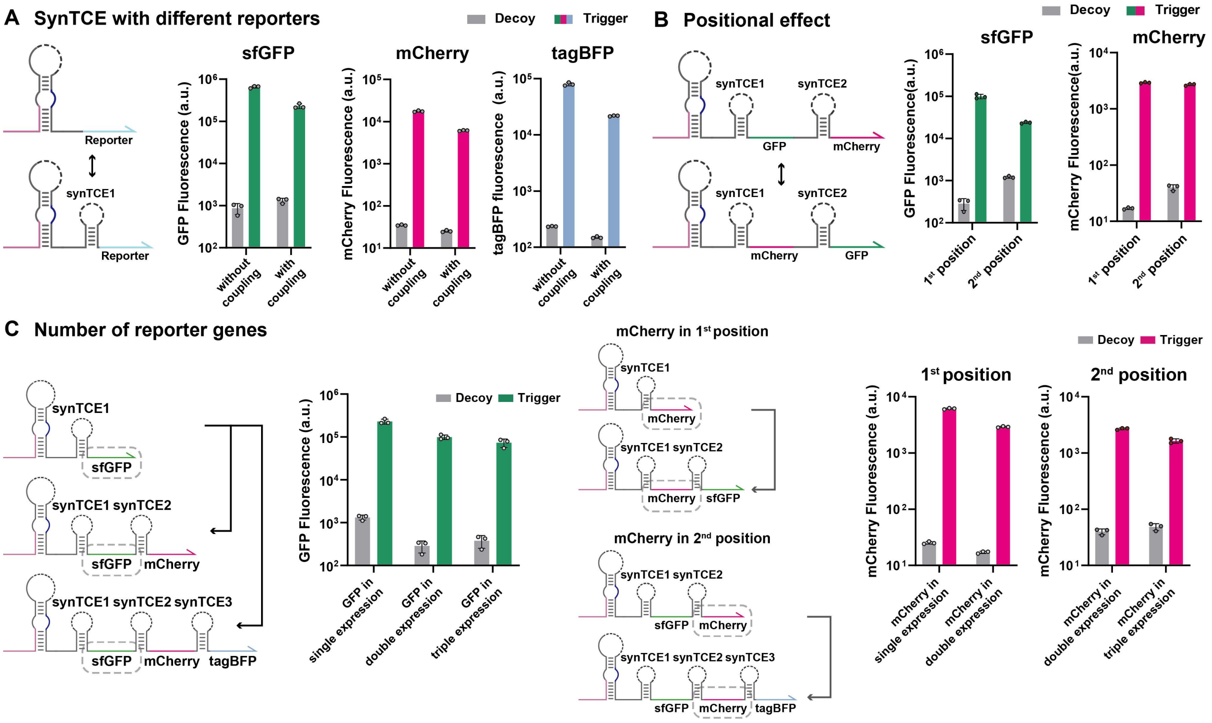


Figure S26. Characterization of synTCE based architecture. (A) SynTCE based architecture with different reporters. Schematic and flow cytometry fluorescence measurement of synTCE with sfGFP, mCherry and tagBFP fluorescence reporters. (B) Positional effect of synTCE and reporter genes. Schematic and flow cytometry measurement of dual-output transcript with inversed position of sfGFP and mCherry. (C) Effect of number of reporter genes on translational coupling. In the case of mCherry, the mCherry in 1st or 2nd position was analyzed. Measurements were taken 3 h 30 min after induction with 0.1 mM IPTG. Dot points specify the individual data, bars represent mean ± s.d. of n=3 biological replicates.

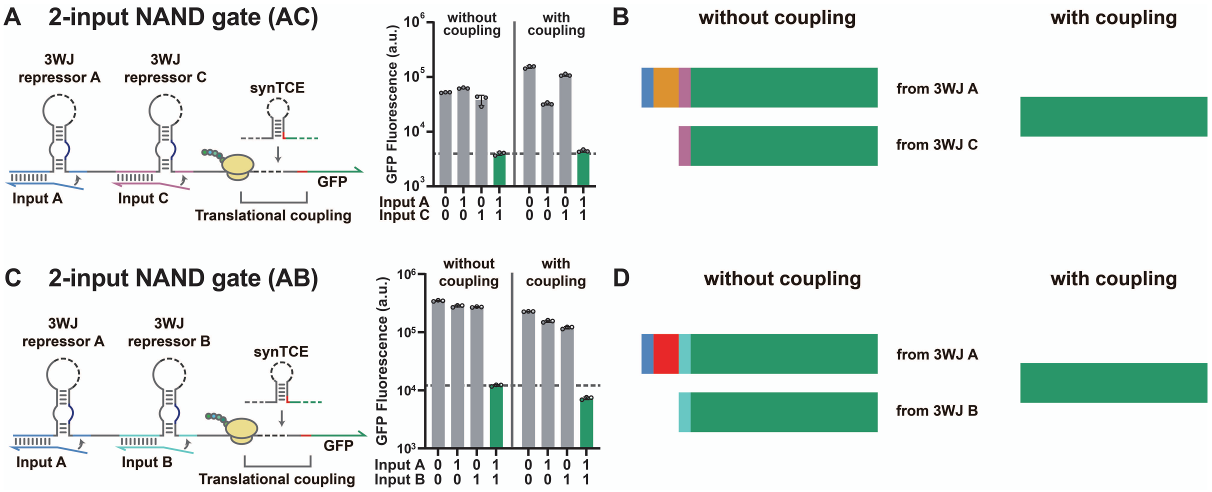


Figure S27. Hypothesis for the reduced GFP fluorescence of NAND AC gate. (A, C) The schematic of the NAND gate architectures either with or without coupling, and the corresponding flow cytometry GFP fluorescence measurements. The measurements were taken 3 h 30 min after induction with 0.1 mM IPTG. Dot points specify the individual data, bars represent mean ± s.d. of n=3 biological replicates. (B, D) Hypothetical GFP proteins translated from each 3WJ repressor either with or without coupling. Green colored bar indicates the native GFP polypeptide. Blue, purple and aqua colored bars represent the polypeptide translated from the corresponding colored 3WJ repressor. Orange and red bars specify the polypeptide encoded by the inter-repressor region. Given that the specific amino acid composition in the N-terminus of protein generated by proteolysis can affect the protein degradation rates (5), we speculate that the GFP protein with the additional peptides on N-terminus when translated from the 3WJ repressor A of the NAND gate (AC) may partly explain the reduced expression level in the absence of synTCE.

# Supplementary Table

Supplementary Table S1. General construct architecture. Abbreviations are as follows: pT7 = T7 promoter, THS = Toehold switch, T7term = T7 terminator, KanR = kanamycin resistance gene, AmpR = ampicillin resistance gene, SpecR = spectinomycin resistance gene, ChlR = chloramphenicol resistance gene.

| Name | Construct order | Plasmid Origin/Resistance |
|---|---|---|
| **Characterization** | | |
| Switch construct | pT7 - THS - Linker - synTCE - GFPmut3b[ASV] - T7term - KanR - ColA origin - LacI | ColA/Kanamycin |
| Trigger construct | pT7 - Trigger - T7term - AmpR - ColE1 origin - LacI | ColE1/ampicillin |
| **Ribocomputing devices** | | |
| 2-input OR gate | pT7 - 2-input OR gate - Linker – synTCE1 - GFPmut3b[ASV] - T7term - KanR - ColA origin - LacI | ColA/Kanamycin |
| Trigger (pCDF) | pT7 - Trigger - T7term - SpecR - CloDF origin - LacI | CloDF/Spectinomycin |
| **Fan-out module** | | |
| Control 1 (open RBS) | pT7 - THS - Linker - synTCE1- sfGFP - Linker2 – open RBS - mCherry - T7term - KanR - ColA origin - LacI | ColA/Kanamycin |
| Multi-output transcript | pT7 - THS - Linker - synTCE1- sfGFP - synTCE- mCherry - T7term - KanR - ColA origin - LacI | ColA/Kanamycin |
| Trigger (pCDF) | pT7 - Trigger - T7term - SpecR - CloDF origin - LacI | CloDF/Spectinomycin |
| Signaling cascade (Sendor) (Ecf11_987/LuxR) | pT7 - THS - Linker - synTCE1- Ecf11_987[AAV] - synTCE- LuxR[ASV] - T7term - KanR - ColA origin - LacI | ColA/Kanamycin |
| Receiver (Ecf11_3726/Plux) | pEcf11_3726 - mCherry - T7term - pLux - GFP[AAV] - B0015terminator - Lambda t0 terminator - AmpR - ColE1 origin - LacI | ColE1/ampicillin |
| Signaling cascade (Sendor) (TetR/Ecf11_987) | pT7 - THS - Linker - synTCE1- TetR[LAA] – synTCE - Ecf11_987[AAV] - T7term - KanR - ColA origin - LacI | ColA/Kanamycin |
| Receiver 1 (PltetO-1) | pLtetO-1 – GFPmut3b [ASV] - T7term - AmpR - ColE1 origin - LacI | ColE1/ampicillin |
| Receiver 2 (Ecf11_3726) | pEcf11_3726 - mCherry - T7term - ChlR - p15A origin - LacI | p15A/Chloramphenicol |
| Multi-input/multi-output circuit | pT7 - 2-input OR gate - Linker – synTCE1 - GFPmut3b[ASV] – synTCE1 – mCherry - T7term - KanR - ColA origin - LacI | ColA/Kanamycin |
| **Signal peptide** | | |
| Original THS + DsbA | pLlacO-1 - THS - Linker - DsbA - GFPmut3b[ASV] - T7term - KanR - ColA origin - LacI | ColA/Kanamycin |
| Coupled THS + DsbA | pLlacO-1 - THS - Linker - synTCE - DsbA - GFPmut3b[ASV] - T7term - KanR - ColA origin - LacI | ColA/Kanamycin |
| **Lysis circuit** | | |
| Original THS + Holin cassette | pLlacO-1 - THS - Linker - S105 gene - R gene - Rz gene (Rz1 gene) - T7term - KanR - ColA origin - LacI | ColA/Kanamycin |
| Coupled THS + Holin cassette | pLlacO-1 - THS - Linker - synTCE - S105 gene - R gene - Rz gene (Rz1 gene) - T7term - KanR - ColA origin - LacI | ColA/Kanamycin |

Supplementary Table S2. Sequence information of riboregulators.

| Name | Sequence | Plasmid origin/resistance | Figure panel |
|---|---|---|---|
| **Single toehold switch (THS)** | | | |
| THS A | ATTGAATATGATAGAAGTTTAGTAGTAGACAATAGAACAGAGGAGATATTGATGACTACTAAACTA | ColA/kanamycin | FIgure 1, 2, Supplementary Figure S2-6, 8, 9, 13, 19, 20, 24-26 |
| THS Trigger A | ATACACATAGAATCATGTGTATAACACTACTAAACTTCTATCATATTCAATCAC | ColE1/ampicillin | |
| THS B | ACTACTATTGATTACACGCTTTACTTCGAAATTCATAATGAACAGAGGAGATATGAAATGCGAAGTAAAGCG | ColA/kanamycin | Figure 4, Supplementary Figure S2, 8, 12, 13, 14, 15, 18 |
| THS Trigger B | CGTGAGATAAGCACATCTCACGAGACGAAGTAAAGCGTGTAATCAATAGTAGTAAG | ColE1/ampicillin | |
| THS C | CTTATGAGTGTAATACGTTCTATGTCAGATTCAAGAACAGAGGAGATTGAAATGGACATAGAACGA | ColA/kanamycin | Figure 4, Supplementary Figure S8, 14-17 |
| THS Trigger C | AGTCTTCAAGATAATGAAGACTCTGGACATAGAACGTATTACACTCATAAGATA | ColE1/ampicillin | |
| THS D | CTTGAATTTGAATTACGTCGTCTTATAGAATTCAGAACAGAGGAGATGAATATGATAAGACGACGA | ColA/kanamycin | Figure 5, Supplementary Figure S22, 23 |
| THS Trigger D | ACGGTCCGGAGTGGCGGACCGTAAGATAAGACGACGTAATTCAAATTCAAGGTA | ColE1/ampicillin | |
| THS E | AGTAAGATAATGAAGGTAGGTATGTTAAACTTTAGAACAGAGGAGATAAAGATGAACATACCTACG | ColA/kanamycin | Supplementary Figure S8 |
| THS Trigger E | CTCGATCACTAATCTGATCGAGACGAACATACCTACCTTCATTATCTTACTTGT | ColE1/ampicillin | |
| THS F | ACTGATTTGAATACACTGCTTCGTTCAAGATTCAGAACAGAGGAGATGAATATGGAACGAAGCAGA | ColA/kanamycin | Supplementary Figure S8 |
| THS Trigger F | AGTTGCGGCACGGACCGCAACTATAGAACGAAGCAGTGTATTCAAATCAGTTAG | ColE1/ampicillin | |
| **Three-way junction (3WJ) repressor** | | | |
| 3WJ repressor A | CTCCTATCACTTTACTTGTTATAGTTATGAACAGAGGAGACATAACATGAACAAGCACACTAACTACAAATTC | ColA/kanamycin | Supplementary Figure S11 |
| 3WJ Trigger A | ACACTAACCATATAGTTAGTGTGCGTAAAGTGATAGGAGTAA | ColE1/ampicillin | |
| 3WJ repressor B | ACTAATCAGATCTACTTGTTATAGTTATGAACAGAGGAGACATAACATGAACAAGCACCTAACAAGACTAATC | ColA/kanamycin | Supplementary Figure S11 |
| 3WJ Trigger B | ACCTAACATAAACTTGTTAGGTGCGTAGATCTGATTAGTGTG | ColE1/ampicillin | |
| 3WJ repressor C | ACAATCAAATACAAATTGTTATAGTTATGAACAGAGGAGACATAACATGAACAATCACATACAAAGCAAACGA | ColA/kanamycin | Supplementary Figure S11 |
| 3WJ Trigger C | ACATACAATGAACTTTGTATGTGATTTGTATTTGATTGTAGC | ColE1/ampicillin | |
| 3WJ repressor D | ATGAATGATATACACTTGTTATAGTTATGAACAGAGGAGACATAACATGAACAAGCACGAATTGACTACACTA | ColA/kanamycin | Supplementary Figure S11 |
| 3WJ Trigger D | GACACTAACCATATAGTTAGTGTGCGTAAAGTGATAGGAGTAA | ColE1/ampicillin | |

| Boolean logic computation | | | |
|---|---|---|---|
| 2-input OR gate | ATGAACTTAAGATCGCGCTTTATGGTTATGAAACAGAGGAGACATAACATGAAAGCGCGAAACAGAACAAGAATAAGTGCAAATGCTTGTTCTATCGTCTTGAAACAGAGGAGACAAGACATGAGAACAAGC | ColA/kanamycin | Figure 3, Supplementary Figure S10, 18 |
| 2OR - Input A | ACCTATTGGACCCGTTTCCAATAGGTGAACAAGACGATAGAACAAGCATTTGCACTTATAGA | ColE1/ampicillin | |
| 2OR - Input B | AGTCGGCGTGGACTCGCACGCCGACTAAACATAACCATAAAGCGCGATCTTAAGTTCATACC | CloDF/Spectinomycin | |
| 6-input OR gate | GTAATGAATTGTAGGCTTGTTATAGTTATGAAACAGAGGAGACATAACATGAACAAGCCTAAACTAAATGAAATAGATTTGATGGCTATTTGTACGTGTTCGAAACAGAGGAGACGAACAATGACAAATAGCACTAACACGAAACGAATAAGACTGAGGCTGGGTATGGTTAGAAAACAGAGGAGATCTAACATGACCCAGCCTAAACATAACGAAATCACTTATTGTCGTCTTTGTATGTCTGTAAAACAGAGGAGATACAGAATGACAAAGACGAAACATGGATATACAAAGATTGGTCGTTTCATTACCGTTAGAAAACAGAGGAGATCTAACATGAATGAAACGACGGGAATTAGAAATTGGATGAAGGCGGTAAGTATGATTGTAAGACAGAGGAGATACAATATGACTTACCGC | ColA/kanamycin | Figure 3, Supplementary Figure S10, 20 |
| 6OR – Input A | ACCGTGGACCGCATGAGGTCCACGGTAAACATAACTATAACAAGCCTACAATTCATTACAAC | ColE1/ampicillin | |
| 6OR – Input B | ACCATTCGCCCTACTTGGCGAATGGTAAGCGAACACGTACAAATAGCCATCAAATCTATACT | ColE1/ampicillin | |
| 6OR – Input C | CCTCGACGTTCGTGATAACGTCGAGGCAATCTAACCATACCCAGCCTCAGTCTTATTCGCAA | ColE1/ampicillin | |
| 6OR – Input D | ACGTCGATTCACCGTCGAATCGACGTAGATACAGACATACAAAGACGACAATAAGTGATAGA | ColE1/ampicillin | |
| 6OR – Input E | ACTCCAGGCGGAATAACGCCTGGAGTAAATCTAACGGTAATGAAACGACCAATCTTTGTATG | ColE1/ampicillin | |
| 6OR – Input F | TCCAGTCTAGACTGACCTAGACTGGACAATACAATCATACTTACCGCCTTCATCCAATTACT | ColE1/ampicillin | |
| 2-input AND gate | ACTACTATTGATTACACGCTTTACTTCGAAATTCATAATGAACAGAGGAGATATGAAATGCGAAGTAAAGCG | ColA/kanamycin | Figure 3, Supplementary Figure S10, 18 |
| 2AND input A | CCACTGGCGAAACAGTGGAAGCGAAGTAAAGCGTGAGATAAGATAAGGATGGTGAAAGTAGA | ColE1/ampicillin | |
| 2AND input B | ACCAGGTAAAGTCCTGGTATAACTTTCACCATCCTTATCTTAACATAATCAATAGTAGTCAA | CloDF/Spectinomycin | |
| 2-input NAND gate (AB) | CTCCTATCACTTTACTTGTTATAGTTATGAACAGAGGAGACATAACATGAACAAGC | ColA/kanamycin | Figure 3, Supplementary Figure S10, 27 |

| | | | |
|---|---|---|---|
| | ACACTAACTACAAATTCGCAAATACATCACAATCAAATACAAATTGTTATAGTTATGAACAGAGGAGACATAACATGAACAATCACATACAAAGCAAACGA | | |
| 2NAND input A | ACACTAACCATATAGTTAGTGTGCGTAAAGTGATAGGAGTAA | ColE1/ampicillin | |
| 2NAND input B | ACATACAATGAACTTTGTATGTGATTTGTATTTGATTGTAGC | CloDF/Spectinomycin | |
| 2-input NAND gate (AC) | CTCCTATCACTTTACTTGTTATAGTTATGAACAGAGGAGACATAACATGAACAAGCACACTAACTACAAATTCCAAAGCCACATACTAATCAGATCTACTTGTTATAGTTATGAACAGAGGAGACATAACATGAACAAGCACCTAACAAGACTAATC | ColA/kanamycin | Supplementary Figure S10, 27 |
| 2NAND input C | ACCTAACATAAACTTGTTAGGTGCGTAGATCTGATTAGTGTG | CloDF/Spectinomycin | |
| 2-input NOT gate | ATTGAATATGATAGAAGTTTAGTAGTAGACAATAGAACAGAGGAGATATTGATGACTACTAAACTA | ColA/kanamycin | Supplementary Figure S10, 19 |
| 2NOT input A | ACTACTAAACTTCTATCATATTCAATATTTTATTTTATTTTG | ColE1/ampicillin | |
| 2NOT input B | CAAAATAAAATAAAACATTGAATATGATAGAAGTTTAGTAGT | CloDF/Spectinomycin | |

Supplementary Table S3. Sequence information of promoters, terminators, and proteins

| Name | Sequence |
|---|---|
| T7 promoter | TAATACGACTCACTATAGGG |
| LlacO-1 promoter | ATAAATGTGAGCGGATAACATTGACATTGTGAGCGGATAACAAGATACTGAGCAC |
| LtetO-1 promoter | TCCCTATCAGTGATAGAGATTGACATCCCTATCAGTGATAGATATACTGAGCAC |
| Ecf11_3726 promoter | GCCTCCACACCGCTCGTCACATCCTGTGATCCACTCTTCATCCCGCTACGTAACACCTCTGCATCGCGAACC<br>AAAACCAG |
| Lux promoter | AGACCTGTAGGATCGTACAGGTTTACGCAAGAAAATGGTTTGTTACTTTCGAATAAA |
| T7 terminator | TAGCATAACCCCTTGGGGCCTCTAAACGGGTCTTGAGGGGTTTTTTG |
| B0015 terminator | CCAGGCATCAAATAAAACGAAAGGCTCAGTCGAAAGACTGGGCCTTTCGTTTTATCTGTTGTTTGTCGGTGA<br>ACGCTCTCTACTAGAGTCACACTGGCTCACCTTCGGGTGGGCCTTTCTGCGTTTATA |
| Lambda t0 terminator | GACTCCTGTTGATAGATCCAGTAATGACCTCAGAACTCCATCTGGATTTGTTCAGAACGCTCGGTTGCCGCC<br>GGGCGTTTTTTATTGGTGAGAAT |
| GFPmut3b [ASV] | ATGCGTAAAGGAGAAGAACTTTTCACTGGAGTTGTCCCAATTCTTGTTGAATTAGATGGTGATGTTAATGGG<br>CACAAATTTTCTGTCAGTGGAGAGGGTGAAGGTGATGCAACATACGGAAAACTTACCCTTAAATTTATTTGC<br>ACTACTGGAAAACTACCTGTTCCGTGGCCAACACTTGTCACTACTTTCGGTTATGGTGTTCAATGCTTTGCG<br>AGATACCCAGATCACATGAAACAGCATGACTTTTTCAAGAGTGCCATGCCCGAAGGTTACGTACAGGAAAGA<br>ACTATATTTTTCAAAGATGACGGGAACTACAAGACACGTGCTGAAGTCAAGTTTGAAGGTGATACCCTTGTT<br>AATAGAATCGAGTTAAAAGGTATTGATTTTAAAGAAGATGGAAACATTCTTGGACACAAATTGGAATACAAC<br>TATAACTCACACAATGTATACATCATGGCAGACAAACAAAAGAATGGAATCAAAGTTAACTTCAAAATTAGA<br>CACAACATTGAAGATGGAAGCGTTCAACTAGCAGACCATTATCAACAAAATACTCCGATTGGCGATGGCCCT<br>GTCCTTTTACCAGACAACCATTACCTGTCCACACAATCTGCCCTTTCGAAAGATCCCAACGAAAAGAGGGAT<br>CACATGGTCCTTCTTGAGTTTGTAACCGCTGCTGGGATTACACATGGCATGGATGAACTATACAAAGGCCT<br>GCAGCAAACGACGAAAACTACGCTGCATCAGTTTAATAA |
| mCherry | ATGCGTAAAGTGAGCAAGGGCGAAGAAGATAACATGGCCATCATCAAGGAGTTCATGCGCTTCAAGGTTCAC<br>ATGGAGGGCTCCGTGAACGGCCACGAGTTCGAGATCGAGGGCGAGGGCGAGGGCCGCCCCTACGAGGGCACC<br>CAGACCGCCAAGCTGAAGGTGACCAAGGGTGGCCCCCTGCCCTTCGCCTGGGACATCCTGTCCCCTCAGTTC<br>ATGTACGGCTCCAAGGCCTACGTGAAGCACCCCGCCGACATCCCCGACTACTTGAAGCTGTCCTTCCCCGAG<br>GGCTTCAAGTGGGAGCGCGTGATGAACTTCGAGGACGGCGGCGTGGTGACCGTGACCCAGGACTCCTCCCTG<br>CAAGACGGCGAGTTCATCTACAAGGTGAAGCTGCGCGGCACCAACTTCCCCTCCGACGGCCCCGTAATGCAG<br>AAGAAGACTATGGGCTGGGAGGCCTCCTCCGAGCGGATGTACCCCGAGGACGGCGCGCTGAAGGGCGAGATC<br>AAGCAGAGGCTGAAGCTGAAGGACGGCGGCCACTACGACGCTGAGGTCAAGACCACCTACAAGGCCAAGAAG<br>CCCGTGCAACTGCCCGGCGCGTACAACGTCAACATCAAGTTGGACATCACCTCCCACAACGAGGACTACACC<br>ATCGTGGAACAGTACGAACGCGCCGAGGGCCGCCACTCCACCGGCGGCATGGACGAGCTGTACAAGTAA |
| sfGFP | ATGAGCAAAGGAGAAGAACTTTTCACTGGAGTTGTCCCAATTCTTGTTGAATTAGATGGTGATGTTAATGGG<br>CACAAATTTTCTGTCCGTGGAGAGGGTGAAGGTGATGCTACAAACGGAAAACTCACCCTTAAATTTATTTGC<br>ACTACTGGAAAACTACCTGTTCCGTGGCCAACACTTGTCACTACTCTGACCTATGGTGTTCAATGCTTTTCC<br>CGTTATCCGGATCACATGAAACGGCATGACTTTTTCAAGAGTGCCATGCCCGAAGGTTATGTACAGGAACGC<br>ACTATATCTTTCAAAGATGACGGGACCTACAAGACGCGTGCTGAAGTCAAGTTTGAAGGTGATACCCTTGTT<br>AATCGTATCGAGTTAAAGGGTATTGATTTTAAAGAAGATGGAAACATTCTTGGACACAAACTCGAGTACAAC<br>TTTAACTCACACAATGTATACATCACGGCAGACAAACAAAAGAATGGAATCAAAGCTAACTTCAAAATTCGC<br>CACAACGTTGAAGATGGTTCCGTTCAACTAGCAGACCATTATCAACAAAATACTCCAATTGGCGATGGCCCT<br>GTCCTTTTACCAGACAACCATTACCTGTCGACACAATCTGTCCTTTCGAAAGATCCCAACGAAAAGCGTGAC<br>CACATGGTCCTTCTTGAGTTTGTAACTGCTGCTGGGATTACACATGGCATGGATGAGCTCTACAAATAA |
| tagBFP | ATGAGCGAGCTGATTAAGGAGAACATGCACATGAAGCTGTACATGGAGGGCACCGTGGACAACCATCACTTC<br>AAGTGCACATCCGAGGGCGAAGGCAAGCCCTACGAGGGCACCCAGACCATGAGAATCAAGGTGGTCGAGGGC<br>GGCCCTCTCCCCTTCGCCTTCGACATCCTGGCTACTAGCTTCCTCTACGGCAGCAAGACCTTCATCAACCAC<br>ACCCAGGGCATCCCCGACTTCTTCAAGCAGTCCTTCCCTGAGGGCTTCACATGGGAGAGAGTCACCACATAC<br>GAAGACGGGGGCGTGCTGACCGCTACCCAGGACACCAGCCTCCAGGACGGCTGCCTCATCTACAACGTCAAG<br>ATCAGAGGGGTGAACTTCACATCCAACGGCCCTGTGATGCAGAAGAAAACACTCGGCTGGGAGGCCTTCACC<br>GAGACGCTGTACCCCGCTGACGGCGGCCTGGAAGGCAGAAACGACATGGCCCTGAAGCTCGTGGGCGGGAGC<br>CATCTGATCGCAAACGCCAAGACCACATATAGATCCAAGAAACCCGCTAAGAACCTCAAGATGCCTGGCGTC<br>TACTATGTGGACTACAGACTGGAAAGAATCAAGGAGGCCAACAACGAGACCTACGTCGAGCAGCACGAGGTG<br>GCAGTGGCCAGATACTGCGACCTCCCTAGCAAACTGGGGCACAAGCTTAATTAA |

| | |
|---|---|
| Ecf11_987 [AAV] | ATGGGCAGCAGCCATCATCATCATCATCACAGCAGCGGCCTGGAAGTTCTGTTCCAGGGGCCCCATATGATG<br>AGCGATAGTCCGCAGAAACTGGGTCGTAATGAATGGAATGCCTATATGGATAAAGTGAAAGCCAAAGATCGT<br>GAAGCCTTTGCATTTGTGTTTCGTTTTTATGCACCGAAACTGAAACAGTTCGCCTATAAACATGTGGGTAAT<br>GAACAGGTTGCCATGGAAATGGTTCAAGAAACCATGGCCACCGTTTGGCAGAAAGCACATCTGTATGATGGT<br>AAAAAAAGCGCACTGAGCACCTGGATTTATACCATTATTCGCAACCTGTGCTTTGATCTGCTGCGTAAACAG<br>AAAGGTAAAGAACTGCATATCCACTCCGATGATATTTGGCCGAGCGAATATTATCCGCCTGATATGGTTGAT<br>CACTATAGTCCGGAACAGGATATGCTGAAAGAACAGGTGGTGAAATTTCTGGATATCCTGCCGAAAAATCAG<br>CGTGATGTTCTGCAGGCAGTTTATCTGGAAGAACTGCCGCATCAGCAGGTTGCAGAACTGTTTGATATTCCG<br>CTGGGCACCGTTAAAAGCCGTCTGCGTCTGGCAGTTGAAAAACTGCGTCATAGCATGCATACCGAACAGCTG<br>AGGCCTGCAGCAAACGACGAAAACTACGCTGCTGCTGTT |
| LuxR [ASV] | ATGAAAAACATAAATGCCGACGACACATACAGAATAATTAATAAAATTAAAGCTTGTAGAAGCAATAATGAT<br>ATTAATCAATGCTTATCTGATATGACTAAAATGGTACATTGTGAATATTATTTACTCGCGATCATTTATCCT<br>CATTCTATGGTTAAATCTGATATTTCAATCCTAGATAATTACCCTAAAAAATGGAGGCAATATTATGATGAC<br>GCTAATTTAATAAAATATGATCCTATAGTAGATTATTCTAACTCCAATCATTCACCAATTAATTGGAATATA<br>TTTGAAAACAATGCTGTAAATAAAAAATCTCCAAATGTAATTAAAGAAGCGAAAACATCAGGTCTTATCACT<br>GGGTTTAGTTTCCCTATTCATACGGCTAACAATGGCTTCGGAATGCTTAGTTTTGCACATTCAGAAAAAGAC<br>AACTATATAGATAGTTTATTTTTACATGCGTGTATGAACATACCATTAATTGTTCCTTCTCTAGTTGATAAT<br>TATCGAAAAATAAATATAGCAAATAATAAATCAAACAACGATTTAACCAAAAGAGAAAAAGAATGTTTAGCG<br>TGGGCATGCGAAGGAAAAAGCTCTTGGGATATTTCAAAAATATTAGGTTGCAGTGAGCGTACTGTCACTTTC<br>CATTTAACCAATGCGCAAATGAAACTCAATACAACAAACCGCTGCCAAAGTATTTCTAAAGCAATTTTAACA<br>GGAGCAATTGATTGCCCATACTTTAAAAATAGGCCTGCAGCAAACGACGAAAACTACGCTGCATCAGTTTAA |
| TetR [LAA] | ATGTCTCGTTTAGATAAAAGTAAAGTGATTAACAGCGCATTAGAGCTGCTTAATGAGGTCGGAATCGAAGGT<br>TTAACAACCCGTAAACTCGCCCAGAAGCTAGGTGTAGAGCAGCCTACATTGTATTGGCATGTAAAAAATAAG<br>CGGGCTTTGCTCGACGCCTTAGCCATTGAGATGTTAGATAGGCACCATACTCACTTTTGCCCTTTAGAAGGG<br>GAAAGCTGGCAAGATTTTTTACGTAATAACGCTAAAAGTTTTAGATGTGCTTTACTAAGTCATCGCGATGGA<br>GCAAAAGTACATTTAGGTACACGGCCTACAGAAAAACAGTATGAAACTCTCGAAAATCAATTAGCCTTTTTA<br>TGCCAACAAGGTTTTTCACTAGAGAATGCATTATATGCACTCAGCGCTGTGGGGCATTTTACTTTAGGTTGC<br>GTATTGGAAGATCAAGAGCATCAAGTCGCTAAAGAAGAAAGGGAAACACCTACTACTGATAGTATGCCGCCA<br>TTATTACGACAAGCTATCGAATTATTTGATCACCAAGGTGCAGAGCCAGCCTTCTTATTCGGCCTTGAATTG<br>ATCATCTGCGGATTAGAAAAACAACTTAAATGTGAAAGTGGGTCTAGGCCTGCTGCTAACGACGAAAACTAC<br>GCTCTGGCCGCC |
| DsbA | ATGAAAAAGATTTGGCTGGCGCTGGCTGGTTTAGTTTTAGCGTTTAGCGCATCGGCGGCGCAGTATGAAGAT<br>CTGGAAGGTCCGGCGGGCTTA |
| Holin cassette (S105-R-Rz(Rz1)) | ATGCCAGAAAAACATGACCTGTTGGCCGCCATTCTCGCGGCAAAGGAACAAGGCATCGGGGCAATCCTTGCG<br>TTTGCAATGGCGTACCTTCGCGGCAGATATAATGGCGGTGCGTTTACAAAAACAGTAATCGACGCAACGATG<br>TGCGCCATTATCGCCTGGTTCATTCGTGACCTTCTCGACTTCGCCGGACTAAGTAGCAATCTCGCTTATATA<br>ACGAGCGTGTTTATCGGCTACATCGGTACTGACTCGATTGGTTCGCTTATCAAACGCTTCGCTGCTAAAAAA<br>GCCGGAGTAGAAGATGGTAGAAATCAATAATCAACGTAAGGCGTTCCTCGATATGCTGGCGTGGTCGGAGGG<br>AACTGATAACGGACGTCAGAAAACCAGAAATCATGGTTATGACGTCATTGTAGGCGGAGAGCTATTTACTGA<br>TTACTCCGATCACCCTCGCAAACTTGTCACGCTAAACCCAAAACTCAAATCAACAGGCGCCGGACGCTACCA<br>GCTTCTTTCCCGTTGGTGGGATGCCTACCGCAAGCAGCTTGGCCTGAAAGACTTCTCTCCGAAAAGTCAGGA<br>CGCTGTGGCATTGCAGCAGATTAAGGAGCGTGGCGCTTTACCTATGATTGATCGTGGTGATATCCGTCAGGC<br>AATCGACCGTTGCAGCAATATCTGGGCTTCACTGCCGGGCGCTGGTTATGGTCAGTTCGAGCATAAGGCTGA<br>CAGCCTGATTGCAAAATTCAAAGAAGCGGGCGGAACGGTCAGAGAGATTGATGTATGAGCAGAGTCACCGCG<br>ATTATCTCCGCTCTGGTTATCTGCATCATCGTCTGCCTGTCATGGGCTGTTAATCATTACCGTGATAACGCC<br>ATTACCTACAAAGCCCAGCGCGACAAAAATGCCAGAGAACTGAAGCTGGCGAACGCGGCAATTACTGACATG<br>CAGATGCGTCAGCGTGATGTTGCTGCGCTCGATGCAAAATACACGAAGGAGTTAGCTGATGCTAAAGCTGAA<br>AATGATGCTCTGCGTGATGATGTTGCCGCTGGTCGTCGTCGGTTGCACATCAAAGCAGTCTGTCAGTCAGTG<br>CGTGAAGCCACCACCGCCTCCGGCGTGGATAATGCAGCCTCCCCCCGACTGGCAGACACCGCTGAACGGGAT<br>TATTTCACCCTCAGAGAGAGGCTGATCACTATGCAAAAACAACTGGAAGGAACCCAGAAGTATATTAATGAG<br>CAGTGCAGATAG |

Supplementary Table S4. Sequence information of characterization of synthetic translational coupling elements (synTCEs). Plasmid sequences used in characterization of translational coupling element can be constructed by replacing the synTCE region in the plasmid's architectures in Table S1 with the appropriate switch sequences described underneath. Red colored indicates Ribosome binding site, bold indicates the stop codon, and underline indicates the start codon.

A. Sequence information of synthetic translational coupling elements (synTCEs)

| Name | Sequence | Structure |
|---|---|---|
| 1 | ACACUCGCAGAGGAGAGCGAG**UAA**UG | ..((((((........)))))).... |
| 2 | CCACCCGCAGAGGAGAGCGGG**UAA**UG | ..((((((........)))))).... |
| 3 | GCACUAGCAGAGGAGAGC**UAG**UUAUG | ..((((((........)))))).... |
| 4 | GGCUAC**UAG**AGGAGAAGUAGCAUG | .((((((........))))))... |
| 5 | GGAGCGGCAGAGGAGAGCCGC**UAA**UG | ..((((((........)))))).... |
| 6 | **UAA**CUCGCAGAGGAGAGCGAGUCAUG | ..((((((........)))))).... |
| Reference | **UGA**CAUAGAGGAGACCUCUAUG | ...((((((((...)))))))) |

B. Sequence information of stem length variants.

| Stem length | Sequence |
|---|---|
| 0 | ACAGAGGAGAGCGAG**UAA**UG |
| 3 | ACCGCAGAGGAGAGCGAG**UAA**UG |
| 6 (synTCE1) | ACACUCGCAGAGGAGAGCGAG**UAA**UG |
| 9 | AGAUUACUCGCAGAGGAGAGCGAG**UAA**UG |
| 12 | ACCGCAUUACUCGCAGAGGAGAGCGAG**UAA**UG |

C. Sequence information of stem base variants. ΔG is calculated with NUPACK software (parameter: material='rna06', ensemble='stacking', celsius=37, sodium=1.0, magnesium=0.0)

| Name | Stem base configuration (5' to 3') | ΔG (kcal/mol) | Sequence |
|---|---|---|---|
| synTCE1 (original stem) | SWSSS | -9.43 | ACACUCGCAGAGGAGAGCGAG**UAA**UG |
| sbm2 | SWSSS | -9.43 | ACAGACGCAGAGGAGAGCGUC**UAA**UG |
| sbm3 | SSSSS | -11.53 | ACACGAACAGAGGAGAGUUCG**UAA**UG |
| sbm4 | SSWWS | -7.03 | ACACCGGCAGAGGAGAGCCGG**UAA**UG |
| sbm5 | SWWSS | -8.03 | ACACAUCCAGAGGAGAGGAUG**UAA**UG |
| sbm6 | SWWSS | -7.83 | ACACAUGCAGAGGAGAGCAUG**UAA**UG |
| sbm7 | SSWSW | -7.08 | ACACGUGUAGAGGAGAACACG**UAA**UG |
| sbm8 | WSSWW | -5.98 | ACAUGGAUAGAGGAGAAUCCA**UAA**UG |
| sbm9 | WSSWW | -5.38 | ACAUCGAUAGAGGAGAAUCGA**UAA**UG |
| sbm10 | WWWWS | -3.33 | ACAAAUACAGAGGAGAGUAUU**UAA**UG |
| sbm11 | WWWWW | -1.28 | ACAAUUAUAGAGGAGAAUAAU**UAA**UG |

D. Sequence information of stem base variants. Translation initiation rate calculation was evaluated with RBS calculator. Open form toehold switch (Upstream 12nt - 1st RBS (5'-

AGAGGAGA-3′) - Linker - synTCE - GFPmut3b) was analyzed on host organism (Escherichia coli BL21 (DE3))

| Name | RBS Sequence | ΔG spacing (kcal/mol) | ΔG rRNA:mRNA (kcal/mol) | ΔG rRNA:mRNA + ΔG spacing (kcal/mol) |
|---|---|---|---|---|
| synTCE1 | AGAGGAGA | 0.3399 | -9.081 | -8.7411 |
| RBS1 | AUUCGCUU | 10.8027 | 1.319 | 12.1217 |
| RBS2 | ACGAAUAC | 10.8027 | 1.319 | 12.1217 |
| RBS3 | UAAUCAAA | 10.8027 | 1.319 | 12.1217 |
| RBS4 | UAUCGGAU | 0 | -4.881 | -4.881 |
| RBS5 | AGGAUGGA | 0.0039 | -6.181 | -6.1771 |
| RBS6 | UAUAGGAU | 0 | -6.481 | -6.481 |
| RBS7 | AGAGGAUA | 0.3399 | -6.481 | -6.1411 |
| RBS8 | ACAGGAGA | 0.3399 | -7.781 | -7.4411 |
| RBS9 | CGAGGAGU | 0.3399 | -9.081 | -8.7411 |
| RBS10 | UGAGGAGA | 0.3399 | -11.681 | -11.3411 |
| RBS11 | GAGGAGAA | 0.8082 | -9.081 | -8.2728 |
| RBS12 | GAGGAGAU | 0.8082 | -9.081 | -8.2728 |

## E. Sequence information of loop size variatns.

| Loop size | Sequence |
|---|---|
| synTCE1 (original loop, 8nt) | ACACUCGCAGAGGAGAGCGAG**UAA**UG |
| 11nt | ACACUCGCGGAAGAGGAGAGCGAG**UAA**UG |
| 14nt | ACACUCGCGAAGAAAGAGGAGAGCGAG**UAA**UG |
| 17nt | ACACUCGCGGAGGGAGUAGAGGAGAGCGAG**UAA**UG |
| 20nt | ACACUCGCGGAGGGGAAAGGAGAGGAGAGCGAG**UAA**UG |

## F. Sequence information of stop codon position variants.

| Name | Intergenic distance from start codon (nt) | Sequence (Linker - synTCE - start codon) | Re-initiation inhibition score |
|---|---|---|---|
| sct1 | -35 | **UGA**GCGCAAAAGAGCCUCGCAGAGGAGAGCGAGGCAUG | -24.29 |
| sct2 | -32 | GCA**UGA**CAAAAGAGCCUCGCAGAGGAGAGCGAGGCAUG | -24.19 |
| sct3 | -29 | GCAGCG**UGA**AAGAGCCUCGCAGAGGAGAGCGAGGCAUG | -15.35 |
| sct4 | -26 | GCAGCGCAA**UGA**AGCCUCGCAGAGGAGAGCGAGGCAUG | -9.635 |
| sct5 | -23 | GCAGCGCAAAAG**UGA**CUCGCAGAGGAGAGCGAGUCAUG | -9.86 |
| sct6 | -20 | GCAGCGCAAAAGAGC**UGA**GCAGAGGAGAGCUCAGCAUG | -8.85 |
| sct7 | -17 | GCAGCGCAAAAGAGCCUC**UGA**GAGGAGACAGAGGCAUG | -6.60 |
| sct8 | -14 | GCAGCGCAAAAGAGCCUCGCA**UGA**GAGAGCGAGGCAUG | -6.31 |
| sct9 | -11 | GCAGCGCAAAAGAGCCUCGCAGAG**UGA**AGCGAGGCAUG | -3.84 |
| sct10 | -8 | GCAGCGCAAAAGAGCCUCUCAGAGGAG**UGA**GAGGCAUG | -3.81 |
| sct11 | -5 | GCAGCGCAAAAGAGCUCAGCAGAGGAGAGC**UGA**GCAUG | -3.54 |
| sct12 | -2 | GCAGCGCAAAAGACACUCGCAGAGGAGAGCGAG**UGA**UG | -3.42 |

## G. Sequence information of 3′ context variants.

| Name | Sequence (36 nt of GOI + Protein linker) |
|---|---|
| sfGFP | ATGAGCAAAGGAGAAGAACTTTTCACTGGAGTTGTCCCAATTCTTGTTGAATTAGAT |
| Cas1 | ATGACCTGGCTTCCCCTTAATCCCATTCCACTCAAACTGGAAGGTCCGGCAGGCTTA |
| Chloramphenicol Resistance gene | ATGGAGAAAAAAATCACTGGATATACCACCGTTGATCTGGAAGGTCCGGCAGGCTTA |

| | |
|---|---|
| Csy4 | ATGGATCACTACATTGATATTCGCGTCCAGCCCGATCTGGAAGGTCCGGCAGGCTTA |
| dCas9 | ATGGATAAGAAATACTCAATAGGCTTAGCTATCGGCCTGGAAGGTCCGGCAGGCTTA |
| Holin | ATGCCAGAAAAACATGACCTGTTGGCCGCCATTCTCCTGGAAGGTCCGGCAGGCTTA |
| LacZ | ATGACCATGATTACGGATTCACTGGCCGTCGTTTTACTGGAAGGTCCGGCAGGCTTA |
| T3 RNA polymerase | ATGAACATCATCGAAAACATCGAAAAGAATGACTTCCTGGAAGGTCCGGCAGGCTTA |
| TetR | ATGTCTAGATTAGATAAAAGTAAAGTGATTAACAGCCTGGAAGGTCCGGCAGGCTTA |

## H. Sequence information of engineered synTCE structure used in Supplementary Figure S24. Engineered region is indicated as bold. RBS is indicated as red color.

| Name | Sequence |
|---|---|
| Original synTCE | ACACTCGCAGAGGAGAGCGAGTA |
| Additional synTCE structure | **GACGGGAUAGAGGAGAAUCCCGUCACACU**CGCAGAGGAGAGCGAGUAAUG |
| Additional RBS sequence | AUUACUCGC**AGAGGAGA**AGAGGAGAGCGAGUAAUG |
| Engineered RBS sequence | GCAUUACUCG**AGGAGGU**CGAGUAAUG |

## I. Sequence information of multi-output transcript.

| Name | Sequence (last 20 nt of sfGFP + Linker/synTCE + first 20 nt of mCherry) |
|---|---|
| Control 1 (open RBS) | GCATGGATGAGCTCTACAAATAAAAGAGACGAACCTGGCGGCAGCGCAAAAGTTCAGCACGCCCGTCTGGA<br>TAAGCCAGGCGCAAGGCATCCGTGCTGGCCCATAAGAATGTAGACAATAGAACAGAGGAGATATTGATGAC<br>TACTAAACTAAACCTGGCGGCAGCGCAAAAGATGCGTAAAGTGAGCAAGGG |
| Control 2 (Multi-switch) | GCATGGATGAGCTCTACAAATAAGATAAACCAGAGCGGCACGGCAAGCAGAGGATTGAATATGATAGAAGT<br>TTAGTAGTAGACAATAGAACAGAGGAGATATTGATGACTACTAAACTAAACCTGGCGGCAGCGCAAAAGAC<br>ACTCGCAGAGGAGAGCGAGTAATGCGTAAAGTGAGCAAGGG |
| No RBS | GCATGGATGAGCTCTACAAAACACTTTACGCATTAGACAAAAGCAACTAATGCGTAAAGTGAGCAAGGG |
| Weak RBS | GCATGGATGAGCTCTACAAAACACTTTACGCATTAGGGACAAGCAACTAATGCGTAAAGTGAGCAAGGG |
| Strong RBS (synTCE1) | GCATGGATGAGCTCTACAAAACACTCGCAGAGGAGAGCGAGTAATGCGTAAAGTGAGCAAGGG |

## J. Sequence information of stoichiometric control.

| Name | ΔG (kcal/mol) | Sequence |
|---|---|---|
| scRBS1<br>(RBS1 in Table S4D) | 10.80 | GCACUAGCAUUCGCUUGC**UAG**UU<u>AUG</u> |
| scRBS2 | -0.92 | GCACUAGCAUUUAACUGC**UAG**UU<u>AUG</u> |
| scRBS3 | -4.55 | GCACUAGCUCUUGUUUGC**UAG**UU<u>AUG</u> |
| scRBS4 | -6.54 | GCACUAGCAUACGAUUGC**UAG**UU<u>AUG</u> |
| scRBS5 | -6.58 | GCACUAGCGUACCCAAGC**UAG**UU<u>AUG</u> |
| scRBS6 | -8.58 | GCACUAGCUCACGGUUGC**UAG**UU<u>AUG</u> |
| scRBS7<br>(RBS4 in Table S4D) | -10.18 | GCACUAGCUAUCGGAUGC**UAG**UU<u>AUG</u> |
| scRBS8 (original RBS) | -15.34 | GCACUAGCAGAGGAGAGC**UAG**UU<u>AUG</u> |
| scRBS9 | -16.08 | GCACUAGCGCACGGAGGC**UAG**UU<u>AUG</u> |

Supplementary Table S5. Representative examples of plasmid full sequences. Replication origin, *lacI* are reversely oriented to other elements. Noncoding backbone sequence is indicated as black color.

| Name | Architecture | Sequence |
| --- | --- | --- |
| Toehold switch with synTCE1 | pT7 – THS - Linker – synTCE1 - GFPmut3b_ASV - T7term - KanR - ColA origin - LacI | TAATACGACTCACTATAGGGATTGAATATGATAGAAGTTTAGTAGTAGACAATAGAACAGAG<br>GAGATATTGATGACTACTAAACTAAACCTGGCGGCAGCGCAAAAGACACTCGCAGAGGAGAG<br>CGAGTAATGCGTAAAGGAGAAGAACTTTTCACTGGAGTTGTCCCAATTCTTGTTGAATTAGA<br>TGGTGATGTTAATGGGCACAAATTTTCTGTCAGTGGAGAGGGTGAAGGTGATGCAACATACG<br>GAAAACTTACCCTTAAATTTATTTGCACTACTGGAAAACTACCTGTTCCGTGGCCAACACTT<br>GTCACTACTTTCGGTTATGGTGTTCAATGCTTTGCGAGATACCCAGATCACATGAAACAGCA<br>TGACTTTTTCAAGAGTGCCATGCCCGAAGGTTACGTACAGGAAAGAACTATATTTTTCAAAG<br>ATGACGGGAACTACAAGACACGTGCTGAAGTCAAGTTTGAAGGTGATACCCTTGTTAATAGA<br>ATCGAGTTAAAAGGTATTGATTTTAAAGAAGATGGAAACATTCTTGGACACAAATTGGAATA<br>CAACTATAACTCACACAATGTATACATCATGGCAGACAAACAAAAGAATGGAATCAAAGTTA<br>ACTTCAAAATTAGACACAACATTGAAGATGGAAGCGTTCAACTAGCAGACCATTATCAACAA<br>AATACTCCGATTGGCGATGGCCCTGTCCTTTTACCAGACAACCATTACCTGTCCACACAATC<br>TGCCCTTTCGAAAGATCCCAACGAAAAGAGGGATCACATGGTCCTTCTTGAGTTTGTAACCG<br>CTGCTGGGATTACACATGGCATGGATGAACTATACAAAAGGCCTGCAGCAAACGACGAAAAC<br>TACGCTGCATCAGTTTAATAAGATAAACCAGAGCGGCACGGCAAGCAGAGTATACGAGATTC<br>GGTAGCCACCGCTGAGCAATAACTAGCATAACCCCTTGGGGCCTCTAAACGGGTCTTGAGGG<br>GTTTTTTGCTGAAACCTCAGGCATTTGAGAAGCACACGGTCACACTGCTTCCGGTAGTCAAT<br>AAACCGGTAAACCAGCAATAGACATAAGCGGCTATTTAACGACCCTGCCCTGAACCGACGAC<br>AAGCTGACGACCGGAACTCCGCAAGTGGCACTTTTCGGGGAAATGTGCGCGGAACCCCTATT<br>TGTTTATTTTTCTAAATACATTCAAATATGTATCCGCTCATGAATTAATTCTTAGAAAAACT<br>CATCGAGCATCAAATGAAACTGCAATTTATTCATATCAGGATTATCAATACCATATTTTTGA<br>AAAAGCCGTTTCTGTAATGAAGGAGAAAACTCACCGAGGCAGTTCCATAGGATGGCAAGATC<br>CTGGTATCGGTCTGCGATTCCGACTCGTCCAACATCAATACAACCTATTAATTTCCCCTCGT<br>CAAAAATAAGGTTATCAAGTGAGAAATCACCATGAGTGACGACTGAATCCGGTGAGAATGGC<br>AAAAGTTTATGCATTTCTTTCCAGACTTGTTCAACAGGCCAGCCATTACGCTCGTCATCAAA<br>ATCACTCGCATCAACCAAACCGTTATTCATTCGTGATTGCGCCTGAGCGAGACGAAATACGC<br>GGTCGCTGTTAAAAGGACAATTACAAACAGGAATCGAATGCAACCGGCGCAGGAACACTGCC<br>AGCGCATCAACAATATTTTCACCTGAATCAGGATATTCTTCTAATACCTGGAATGCTGTTTT<br>CCCGGGGATCGCAGTGGTGAGTAACCATGCATCATCAGGAGTACGGATAAAATGCTTGATGG<br>TCGGAAGAGGCATAAATTCCGTCAGCCAGTTTAGTCTGACCATCTCATCTGTAACATCATTG<br>GCAACGCTACCTTTGCCATGTTTCAGAAACAACTCTGGCGCATCGGGCTTCCCATACAATCG<br>ATAGATTGTCGCACCTGATTGCCCGACATTATCGCGAGCCCATTTATACCCATATAAATCAG<br>CATCCATGTTGGAATTTAATCGCGGCCTAGAGCAAGACGTTTCCCGTTGAATATGGCTCATA<br>CTCTTCCTTTTTCAATATTATTGAAGCATTTATCAGGGTTATTGTCTCATGAGCGGATACAT<br>ATTTGAATGTATTTAGAAAAATAAACAAATAGGCATGCTAGCGCAGAAACGTCCTAGAAGAT<br>GCCAGGAGGATACTTAGCAGAGAGACAATAAGGCCGGAGCGAAGCCGTTTTTCCATAGGCTC<br>CGCCCCCCTGACGAACATCACGAAATCTGACGCTCAAATCAGTGGTGGCGAAACCCGACAGG<br>ACTATAAAGATACCAGGCGTTTCCCCCTGATGGCTCCCTCTTGCGCTCTCCTGTTCCCGTCC<br>TGCGGCGTCCGTGTTGTGGTGGAGGCTTTACCCAAATCACCACGTCCCGTTCCGTGTAGACA<br>GTTCGCTCCAAGCTGGGCTGTGTGCAAGAACCCCCCGTTCAGCCCGACTGCTGCGCCTTATC<br>CGGTAACTATCATCTTGAGTCCAACCCGGAAAGACACGACAAAACGCCACTGGCAGCAGCCA<br>TTGGTAACTGAGAATTAGTGGATTTAGATATCGAGAGTCTTGAAGTGGTGGCCTAACAGAGG<br>CTACACTGAAAGGACAGTATTTGGTATCTGCGCTCCACTAAAGCCAGTTACCAGGTTAAGCA<br>GTTCCCCAACTGACTTAACCTTCGATCAAACCGCCTCCCCAGGCGGTTTTTTCGTTTACAGA<br>GCAGGAGATTACGACGATCGTAAAAGGATCTCAAGAAGATCCTTTACGGATTCCCGACACCA<br>TCACTCTAGATTTCAGTGCAATTTATCTCTTCAAATGTAGCACCTGAAGTCAGCCCCATACG<br>ATATAAGTTGTAATTCTCATGTTAGTCATGCCCCGCGCCCACCGGAAGGAGCTGACTGGGTT<br>GAAGGCTCTCAAGGGCATCGGTCGAGATCCCGGTGCCTAATGAGTGAGCTAACTTACATTAA<br>TTGCGTTGCGCTCACTGCCCGCTTTCCAGTCGGGAAACCTGTCGTGCCAGCTGCATTAATGA<br>ATCGGCCAACGCGCGGGGAGAGGCGGTTTGCGTATTGGGCGCCAGGGTGGTTTTTCTTTTCA<br>CCAGTGAGACGGGCAACAGCTGATTGCCCTTCACCGCCTGGCCCTGAGAGAGTTGCAGCAAG<br>CGGTCCACGCTGGTTTGCCCCAGCAGGCGAAAATCCTGTTTGATGGTGGTTAACGGCGGGAT<br>ATAACATGAGCTGTCTTCGGTATCGTCGTATCCCACTACCGAGATGTCCGCACCAACGCGCA<br>GCCCGGACTCGGTAATGGCGCGCATTGCGCCCAGCGCCATCTGATCGTTGGCAACCAGCATC<br>GCAGTGGGAACGATGCCCTCATTCAGCATTTGCATGGTTTGTTGAAAACCGGACATGGCACT<br>CCAGTCGCCTTCCCGTTCCGCTATCGGCTGAATTTGATTGCGAGTGAGATATTTATGCCAGC<br>CAGCCAGACGCAGACGCGCCGAGACAGAACTTAATGGGCCCGCTAACAGCGCGATTTGCTGG<br>TGACCCAATGCGACCAGATGCTCCACGCCCAGTCGCGTACCGTCTTCATGGGAGAAAATAAT<br>ACTGTTGATGGGTGTCTGGTCAGAGACATCAAGAAATAACGCCGGAACATTAGTGCAGGCAG<br>CTTCCACAGCAATGGCATCCTGGTCATCCAGCGGATAGTTAATGATCAGCCCACTGACGCGT<br>TGCGCGAGAAGATTGTGCACCGCCGCTTTACAGGCTTCGACGCCGCTTCGTTCTACCATCGA<br>CACCACCACGCTGGCACCCAGTTGATCGGCGCGAGATTTAATCGCCGCGACAATTTGCGACG<br>GCGCGTGCAGGGCCAGACTGGAGGTGGCAACGCCAATCAGCAACGACTGTTTGCCCGCCAGT<br>TGTTGTGCCACGCGGTTGGGAATGTAATTCAGCTCCGCCATCGCCGCTTCCACTTTTTCCCG<br>CGTTTTCGCAGAAACGTGGCTGGCCTGGTTCACCACGCGGGAAACGGTCTGATAAGAGACAC |

| | | |
|---|---|---|
| | | CGGCATACTCTGCGACATCGTATAACGTTACTGGTTTCACATTCACCACCCTGAATTGACTC<br>TCTTCCGGGCGCTATCATGCCATACCGCGAAAGGTTTTGCGCCATTCGATGGTGTCCGGGAT<br>CTCGACGCTCTCCCTTATGAAGTCTAACGCTGCTCTGGGCTAACTGTCGCGC |
| Trigger | pT7 - Trigger - T7term - AmpR – pBR322 origin - LacI | TAATACGACTCACTATAGGGATACACATAGAATCATGTGTATAACACTACTAAACTTCTATC<br>ATATTCAATCACTAGCATAACCCCTTGGGGCCTCTAAACGGGTCTTGAGGGGTTTTTTGCAC<br>TTTCGCAAAACTGAGAGCTCACGTGTACAGTACCGGCATAACCAAGCCTATGCCTACAGCAT<br>CCAGGGTGACGGTGCCGAGGATGACGATGAGCGCATTGTTAGATTTCATACACGGTGCCTGA<br>CTGCGTTAGCAATTTAACTGTGATAAACTACCGCATTAAAGCTTATCGATGATAAGCTGTCA<br>AACATGAGAATTCTTGAAGACGAAAGGGCCTCGTGATACGCCTATTTTTATAGGTTAATGTC<br>ATGATAATAATGGTTTCTTAGACGTCAGGTGGCACTTTTCGGGGAAATGTGCGCGGAACCCC<br>TATTTGTTTATTTTTCTAAATACATTCAAATATGTATCCGCTCATGAGACAATAACCCTGAT<br>AAATGCTTCAATAATATTGAAAAAGGAAGAGTATGAGTATTCAACATTTCCGTGTCGCCCTT<br>ATTCCCTTTTTTGCGGCATTTTGCCTTCCTGTTTTTGCTCACCCAGAAACGCTGGTGAAAGT<br>AAAAGATGCTGAAGATCAGTTGGGTGCACGAGTGGGTTACATCGAACTGGATCTCAACAGCG<br>GTAAGATCCTTGAGAGTTTTCGCCCCGAAGAACGTTTTCCAATGATGAGCACTTTTAAAGTT<br>CTGCTATGTGGCGCGGTATTATCCCGTGTTGACGCCGGGCAAGAGCAACTCGGTCGCCGCAT<br>ACACTATTCTCAGAATGACTTGGTTGAGTACTCACCAGTCACAGAAAAGCATCTTACGGATG<br>GCATGACAGTAAGAGAATTATGCAGTGCTGCCATAACCATGAGTGATAACACTGCGGCCAAC<br>TTACTTCTGACAACGATCGGAGGACCGAAGGAGCTAACCGCTTTTTTGCACAACATGGGGGA<br>TCATGTAACTCGCCTTGATCGTTGGGAACCGGAGCTGAATGAAGCCATACCAAACGACGAGC<br>GTGACACCACGATGCCTGCAGCAATGGCAACAACGTTGCGCAAACTATTAACTGGCGAACTA<br>CTTACTCTAGCTTCCCGGCAACAATTAATAGACTGGATGGAGGCGGATAAAGTTGCAGGACC<br>ACTTCTGCGCTCGGCCCTTCCGGCTGGCTGGTTTATTGCTGATAAATCTGGAGCCGGTGAGC<br>GTGGGTCTCGCGGTATCATTGCAGCACTGGGGCCAGATGGTAAGCCCTCCCGTATCGTAGTT<br>ATCTACACGACGGGGAGTCAGGCAACTATGGATGAACGAAATAGACAGATCGCTGAGATAGG<br>TGCCTCACTGATTAAGCATTGGTAACTGTCAGACCAAGTTTACTCATATATACTTTAGATTG<br>ATTTAAAACTTCATTTTTAATTTAAAAGGATCTAGGTGAAGATCCTTTTTGATAATCTCATG<br>ACCAAAATCCCTTAACGTGAGTTTTCGTTCCACTGAGCGTCAGACCCCGTAGAAAAGATCAA<br>AGGATCTTCTTGAGATCCTTTTTTTCTGCGCGTAATCTGCTGCTTGCAAACAAAAAAACCAC<br>CGCTACCAGCGGTGGTTTGTTTGCCGGATCAAGAGCTACCAACTCTTTTTCCGAAGGTAACT<br>GGCTTCAGCAGAGCGCAGATACCAAATACTGTCCTTCTAGTGTAGCCGTAGTTAGGCCACCA<br>CTTCAAGAACTCTGTAGCACCGCCTACATACCTCGCTCTGCTAATCCTGTTACCAGTGGCTG<br>CTGCCAGTGGCGATAAGTCGTGTCTTACCGGGTTGGACTCAAGACGATAGTTACCGGATAAG<br>GCGCAGCGGTCGGGCTGAACGGGGGGTTCGTGCACACAGCCCAGCTTGGAGCGAACGACCTA<br>CACCGAACTGAGATACCTACAGCGTGAGCTATGAGAAAGCGCCACGCTTCCCGAAGGGAGAA<br>AGGCGGACAGGTATCCGGTAAGCGGCAGGGTCGGAACAGGAGAGCGCACGAGGGAGCTTCCA<br>GGGGGAAACGCCTGGTATCTTTATAGTCCTGTCGGGTTTCGCCACCTCTGACTTGAGCGTCG<br>ATTTTTGTGATGCTCGTCAGGGGGGCGGAGCCTATGGAAAAACGCCAGCAACGCGGCCTTTT<br>TACGGTTCCTGGCCTTTTGCTGGCCTTTTGCTCACATGTTCTTTCCTGCGTTATCCCCTGAT<br>TCTGTGGATAACCGTATTACCGCCTTTGAGTGAGCTGATACCGCTCGCCGCAGCCGAACGAC<br>CGAGCGCAGCGAGTCAGTGAGCGAGGAAGCGGAAGAGCGCCTGATGCGGTATTTTCTCCTTA<br>CGCATCTGTGCGGTATTTCACACCGCATATATGGTGCACTCTCAGTACAATCTGCTCTGATG<br>CCGCATAGTTAAGCCAGTATACACTCCGCTATCGCTACGTGACTGGGTCATGGCTGCGCCCC<br>GACACCCGCCAACACCCGCTGACGCGCCCTGACGGGCTTGTCTGCTCCCGGCATCCGCTTAC<br>AGACAAGCTGTGACCGTCTCCGGGAGCTGCATGTGTCAGAGGTTTTCACCGTCATCACCGAA<br>ACGCGCGAGGCAGCTGCGGTAAAGCTCATCAGCGTGGTCGTGAAGCGATTCACAGATGTCTG<br>CCTGTTCATCCGCGTCCAGCTCGTTGAGTTTCTCCAGAAGCGTTAATGTCTGGCTTCTGATA<br>AAGCGGGCCATGTTAAGGGCGGTTTTTTCCTGTTTGGTCACTGATGCCTCCGTGTAAGGGGG<br>ATTTCTGTTCATGGGGGTAATGATACCGATGAAACGAGAGAGGATGCTCACGATACGGGTTA<br>CTGATGATGAACATGCCCGGTTACTGGAACGTTGTGAGGGTAAACAACTGGCGGTATGGATG<br>CGGCGGGACCAGAGAAAAATCACTCAGGGTCAATGCCAGCGCTTCGTTAATACAGATGTAGG<br>TGTTCCACAGGGTAGCCAGCAGCATCCTGCGATGCAGATCCGGAACATAATGGTGCAGGGCG<br>CTGACTTCCGCGTTTCCAGACTTTACGAAACACGGAAACCGAAGACCATTCATGTTGTTGCT<br>CAGGTCGCAGACGTTTTGCAGCAGCAGTCGCTTCACGTTCGCTCGCGTATCGGTGATTCATT<br>CTGCTAACCAGTAAGGCAACCCCGCCAGCCTAGCCGGGTCCTCAACGACAGGAGCACGATCA<br>TGCGCACCCGTGGCCAGGACCCAACGCTGCCCGAGATGCGCCGCGTGCGGCTGCTGGAGATG<br>GCGGACGCGATGGATATGTTCTGCCAAGGGTTGGTTTGCGCATTCACAGTTCTCCGCAAGAA<br>TTGATTGGCTCCAATTCTTGGAGTGGTGAATCCGTTAGCGAGGTGCCGCCGGCTTCCATTCA<br>GGTCGAGGTGGCCCGGCTCCATGCACCGCGACGCAACGCGGGGAGGCAGACAAGGTATAGGG<br>CGGCGCCTACAATCCATGCCAACCCGTTCCATGTGCTCGCCGAGGCGGCATAAATCGCCGTG<br>ACGATCAGCGGTCCAGTGATCGAAGTTAGGCTGGTAAGAGCCGCGAGCGATCCTTGAAGCTG<br>TCCCTGATGGTCGTCATCTACCTGCCTGGACAGCATGGCCTGCAACGCGGGCATCCCGATGC<br>CGCCGGAAGCGAGAAGAATCATAATGGGGAAGGCCATCCAGCCTCGCGTCGCGAACGCCAGC<br>AAGACGTAGCCCAGCGCGTCGGCCGCCATGCCGGCGATAATGGCCTGCTTCTCGCCGAAACG<br>TTTGGTGGCGGGACCAGTGACGAAGGCTTGAGCGAGGGCGTGCAAGATTCCGAATACCGCAA<br>GCGACAGGCCGATCATCGTCGCGCTCCAGCGAAAGCGGTCCTCGCCGAAAATGACCCAGAGC<br>GCTGCCGGCACCTGTCCTACGAGTTGCATGATAAAGAAGACAGTCATAAGTGCGGCGACGAT<br>AGTCATGCCCCGCGCCCACCGGAAGGAGCTGACTGGGTTGAAGGCTCTCAAGGGCATCGGTC<br>GAGATCCCGGTGCCTAATGAGTGAGCTAACTTACATTAATTGCGTTGCGCTCACTGCCCGCT<br>TTCCAGTCGGGAAACCTGTCGTGCCAGCTGCATTAATGAATCGGCCAACGCGCGGGGAGAGG<br>CGGTTTGCGTATTGGGCGCCAGGGTGGTTTTTCTTTTCACCAGTGAGACGGGCAACAGCTGA<br>TTGCCCTTCACCGCCTGGCCCTGAGAGAGTTGCAGCAAGCGGTCCACGCTGGTTTGCCCCAG<br>CAGGCGAAAATCCTGTTTGATGGTGGTTAACGGCGGGATATAACATGAGCTGTCTTCGGTAT<br>CGTCGTATCCCACTACCGAGATATCCGCACCAACGCGCAGCCCGGACTCGGTAATGGCGCGC |

| | | |
|---|---|---|
| | | ATTGCGCCCAGCGCCATCTGATCGTTGGCAACCAGCATCGCAGTGGGAACGATGCCCTCATT<br>CAGCATTTGCATGGTTTGTTGAAAACCGGACATGGCACTCCAGTCGCCTTCCCGTTCCGCTA<br>TCGGCTGAATTTGATTGCGAGTGAGATATTTATGCCAGCCAGCCAGACGCAGACGCGCCGAG<br>ACAGAACTTAATGGGCCCGCTAACAGCGCGATTTGCTGGTGACCCAATGCGACCAGATGCTC<br>CACGCCCAGTCGCGTACCGTCTTCATGGGAGAAAATAATACTGTTGATGGGTGTCTGGTCAG<br>AGACATCAAGAAATAACGCCGGAACATTAGTGCAGGCAGCTTCCACAGCAATGGCATCCTGG<br>TCATCCAGCGGATAGTTAATGATCAGCCCACTGACGCGTTGCGCGAGAAGATTGTGCACCGC<br>CGCTTTACAGGCTTCGACGCCGCTTCGTTCTACCATCGACACCACCACGCTGGCACCCAGTT<br>GATCGGCGCGAGATTTAATCGCCGCGACAATTTGCGACGGCGCGTGCAGGGCCAGACTGGAG<br>GTGGCAACGCCAATCAGCAACGACTGTTTGCCCGCCAGTTGTTGTGCCACGCGGTTGGGAAT<br>GTAATTCAGCTCCGCCATCGCCGCTTCCACTTTTTCCCGCGTTTTCGCAGAAACGTGGCTGG<br>CCTGGTTCACCACGCGGGAAACGGTCTGATAAGAGACACCGGCATACTCTGCGACATCGTAT<br>AACGTTACTGGTTTCACATTCACCACCCTGAATTGACTCTCTTCCGGGCGCTATCATGCCAT<br>ACCGCGAAAGGTTTTGCGCCATTCGATGGTGTCCGGGATCTCGACGCTCTCCCTTATGCGAC<br>TCCTGCATTAGGAAGCAGCCCAGTAGTAGGTTGAGGCCGTTGAGCACCGCCGCCGCAAGGAA<br>TGGTGCATGCAAGGAGATGGCGCCCAACAGTCCCCCGGCCACGGGGCCTGCCACCATACCCA<br>CGCCGAAACAAGCGCTCATGAGCCCGAAGTGGCGAGCCCGATCTTCCCCATCGGTGATGTCG<br>GCGATATAGGCGCCAGCAACCGCACCTGTGGCGCCGGTGATGCCGGCCACGATGCGTCCGGC<br>GTAGAGGATCGAGATCTCGAGTGATAGCCGTTTGTCTGGTGTCTACGCCGCGC |
| 2-output generator | pT7 - THS - Linker - synTCE1- Ecf11_987_AAV – synTCE2 - LuxR_ASV - T7term - KanR - ColA origin - LacI | TAATACGACTCACTATAGGGACTACTATTGATTACACGCTTTACTTCGAAATTCATAATGAA<br>CAGAGGAGATATGAAATGCGAAGTAAAGCGAACCTGGCGGCAGCGCAAAAGACACTCGCAGA<br>GGAGAGCGAGTAATGGGCAGCAGCCATCATCATCATCATCACAGCAGCGGCCTGGAAGTTCT<br>GTTCCAGGGGCCCCATATGATGAGCGATAGTCCGCAGAAACTGGGTCGTAATGAATGGAATG<br>CCTATATGGATAAAGTGAAAGCCAAAGATCGTGAAGCCTTTGCATTTGTGTTTCGTTTTTAT<br>GCACCGAAACTGAAACAGTTCGCCTATAAACATGTGGGTAATGAACAGGTTGCCATGGAAAT<br>GGTTCAAGAAACCATGGCCACCGTTTGGCAGAAAGCACATCTGTATGATGGTAAAAAAAGCG<br>CACTGAGCACCTGGATTTATACCATTATTCGCAACCTGTGCTTTGATCTGCTGCGTAAACAG<br>AAAGGTAAAGAACTGCATATCCACTCCGATGATATTTGGCCGAGCGAATATTATCCGCCTGA<br>TATGGTTGATCACTATAGTCCGGAACAGGATATGCTGAAAGAACAGGTGGTGAAATTTCTGG<br>ATATCCTGCCGAAAAATCAGCGTGATGTTCTGCAGGCAGTTTATCTGGAAGAACTGCCGCAT<br>CAGCAGGTTGCAGAACTGTTTGATATTCCGCTGGGCACCGTTAAAAGCCGTCTGCGTCTGGC<br>AGTTGAAAAACTGCGTCATAGCATGCATACCGAACAGCTGAGGCCTGCAGCAAACGACGAAA<br>ACTACGCTGCTGCTGTTGCACTAGCAGAGGAGAGCTAGTTATGAAAAACATAAATGCCGACG<br>ACACATACAGAATAATTAATAAAATTAAAGCTTGTAGAAGCAATAATGATATTAATCAATGC<br>TTATCTGATATGACTAAAATGGTACATTGTGAATATTATTTACTCGCGATCATTTATCCTCA<br>TTCTATGGTTAAATCTGATATTTCAATCCTAGATAATTACCCTAAAAAATGGAGGCAATATT<br>ATGATGACGCTAATTTAATAAAATATGATCCTATAGTAGATTATTCTAACTCCAATCATTCA<br>CCAATTAATTGGAATATATTTGAAAACAATGCTGTAAATAAAAAATCTCCAAATGTAATTAA<br>AGAAGCGAAAACATCAGGTCTTATCACTGGGTTTAGTTTCCCTATTCATACGGCTAACAATG<br>GCTTCGGAATGCTTAGTTTTGCACATTCAGAAAAAGACAACTATATAGATAGTTTATTTTTA<br>CATGCGTGTATGAACATACCATTAATTGTTCCTTCTCTAGTTGATAATTATCGAAAAATAAA<br>TATAGCAAATAATAAATCAAACAACGATTTAACCAAAAGAGAAAAAGAATGTTTAGCGTGGG<br>CATGCGAAGGAAAAAGCTCTTGGGATATTTCAAAAATATTAGGTTGCAGTGAGCGTACTGTC<br>ACTTTCCATTTAACCAATGCGCAAATGAAACTCAATACAACAAACCGCTGCCAAAGTATTTC<br>TAAAGCAATTTTAACAGGAGCAATTGATTGCCCATACTTTAAAAATAGGCCTGCAGCAAACG<br>ACGAAAACTACGCTGCATCAGTTTAAGGATCCAAGCTTGCGGCCGCACTCGAGCACCACCAC<br>CACCACCACTGAGATCCGGCTGCTAACAAAGCCCGAAAGGAAGCTGAGTTGGCTGCTGCCAC<br>CGCTGAGCAATAACTAGCATAACCCCTTGGGGCCTCTAAACGGGTCTTGAGGGGTTTTTTGC<br>TGAAACCTCAGGCATTTGAGAAGCACACGGTCACACTGCTTCCGGTAGTCAATAAACCGGTA<br>AACCAGCAATAGACATAAGCGGCTATTTAACGACCCTGCCCTGAACCGACGACAAGCTGACG<br>ACCGGAACTCCGCAAGTGGCACTTTTCGGGGAAATGTGCGCGGAACCCCTATTTGTTTATTT<br>TTCTAAATACATTCAAATATGTATCCGCTCATGAATTAATTCTTAGAAAAACTCATCGAGCA<br>TCAAATGAAACTGCAATTTATTCATATCAGGATTATCAATACCATATTTTTGAAAAAGCCGT<br>TTCTGTAATGAAGGAGAAAACTCACCGAGGCAGTTCCATAGGATGGCAAGATCCTGGTATCG<br>GTCTGCGATTCCGACTCGTCCAACATCAATACAACCTATTAATTTCCCCTCGTCAAAAATAA<br>GGTTATCAAGTGAGAAATCACCATGAGTGACGACTGAATCCGGTGAGAATGGCAAAAGTTTA<br>TGCATTTCTTTCCAGACTTGTTCAACAGGCCAGCCATTACGCTCGTCATCAAAATCACTCGC<br>ATCAACCAAACCGTTATTCATTCGTGATTGCGCCTGAGCGAGACGAAATACGCGGTCGCTGT<br>TAAAAGGACAATTACAAACAGGAATCGAATGCAACCGGCGCAGGAACACTGCCAGCGCATCA<br>ACAATATTTTCACCTGAATCAGGATATTCTTCTAATACCTGGAATGCTGTTTTCCCGGGGAT<br>CGCAGTGGTGAGTAACCATGCATCATCAGGAGTACGGATAAAATGCTTGATGGTCGGAAGAG<br>GCATAAATTCCGTCAGCCAGTTTAGTCTGACCATCTCATCTGTAACATCATTGGCAACGCTA<br>CCTTTGCCATGTTTCAGAAACAACTCTGGCGCATCGGGCTTCCCATACAATCGATAGATTGT<br>CGCACCTGATTGCCCGACATTATCGCGAGCCCATTTATACCCATATAAATCAGCATCCATGT<br>TGGAATTTAATCGCGGCCTAGAGCAAGACGTTTCCCGTTGAATATGGCTCATACTCTTCCTT<br>TTTCAATATTATTGAAGCATTTATCAGGGTTATTGTCTCATGAGCGGATACATATTTGAATG<br>TATTTAGAAAAATAAACAAATAGGCATGCTAGCGCAGAAACGTCCTAGAAGATGCCAGGAGG<br>ATACTTAGCAGAGAGACAATAAGGCCGGAGCGAAGCCGTTTTTCCATAGGCTCCGCCCCCCT<br>GACGAACATCACGAAATCTGACGCTCAAATCAGTGGTGGCGAAACCCGACAGGACTATAAAG<br>ATACCAGGCGTTTCCCCCTGATGGCTCCCTCTTGCGCTCTCCTGTTCCCGTCCTGCGGCGTC<br>CGTGTTGTGGTGGAGGCTTTACCCAAATCACCACGTCCCGTTCCGTGTAGACAGTTCGCTCC<br>AAGCTGGGCTGTGTGCAAGAACCCCCCGTTCAGCCCGACTGCTGCGCCTTATCCGGTAACTA<br>TCATCTTGAGTCCAACCCGGAAAGACACGACAAAACGCCACTGGCAGCAGCCATTGGTAACT<br>GAGAATTAGTGGATTTAGATATCGAGAGTCTTGAAGTGGTGGCCTAACAGAGGCTACACTGA<br>AAGGACAGTATTTGGTATCTGCGCTCCACTAAAGCCAGTTACCAGGTTAAGCAGTTCCCCAA |

| | | |
|---|---|---|
| | | CTGACTTAACCTTCGATCAAACCGCCTCCCCAGGCGGTTTTTTCGTTTACAGAGCAGGAGAT<br>TACGACGATCGTAAAAGGATCTCAAGAAGATCCTTTACGGATTCCCGACACCATCACTCTAG<br>ATTTCAGTGCAATTTATCTCTTCAAATGTAGCACCTGAAGTCAGCCCCATACGATATAAGTT<br>GTAATTCTCATGTTAGTCATGCCCCGCGCCCACCGGAAGGAGCTGACTGGGTTGAAGGCTCT<br>CAAGGGCATCGGTCGAGATCCCGGTGCCTAATGAGTGAGCTAACTTACATTAATTGCGTTGC<br>GCTCACTGCCCGCTTTCCAGTCGGGAAACCTGTCGTGCCAGCTGCATTAATGAATCGGCCAA<br>CGCGCGGGGAGAGGCGGTTTGCGTATTGGGCGCCAGGGTGGTTTTTCTTTTCACCAGTGAGA<br>CGGGCAACAGCTGATTGCCCTTCACCGCCTGGCCCTGAGAGAGTTGCAGCAAGCGGTCCACG<br>CTGGTTTGCCCCAGCAGGCGAAAATCCTGTTTGATGGTGGTTAACGGCGGGATATAACATGA<br>GCTGTCTTCGGTATCGTCGTATCCCACTACCGAGATGTCCGCACCAACGCGCAGCCCGGACT<br>CGGTAATGGCGCGCATTGCGCCCAGCGCCATCTGATCGTTGGCAACCAGCATCGCAGTGGGA<br>ACGATGCCCTCATTCAGCATTTGCATGGTTTGTTGAAAACCGGACATGGCACTCCAGTCGCC<br>TTCCCGTTCCGCTATCGGCTGAATTTGATTGCGAGTGAGATATTTATGCCAGCCAGCCAGAC<br>GCAGACGCGCCGAGACAGAACTTAATGGGCCCGCTAACAGCGCGATTTGCTGGTGACCCAAT<br>GCGACCAGATGCTCCACGCCCAGTCGCGTACCGTCTTCATGGGAGAAAATAATACTGTTGAT<br>GGGTGTCTGGTCAGAGACATCAAGAAATAACGCCGGAACATTAGTGCAGGCAGCTTCCACAG<br>CAATGGCATCCTGGTCATCCAGCGGATAGTTAATGATCAGCCCACTGACGCGTTGCGCGAGA<br>AGATTGTGCACCGCCGCTTTACAGGCTTCGACGCCGCTTCGTTCTACCATCGACACCACCAC<br>GCTGGCACCCAGTTGATCGGCGCGAGATTTAATCGCCGCGACAATTTGCGACGGCGCGTGCA<br>GGGCCAGACTGGAGGTGGCAACGCCAATCAGCAACGACTGTTTGCCCGCCAGTTGTTGTGCC<br>ACGCGGTTGGGAATGTAATTCAGCTCCGCCATCGCCGCTTCCACTTTTTCCCGCGTTTTCGC<br>AGAAACGTGGCTGGCCTGGTTCACCACGCGGGAAACGGTCTGATAAGAGACACCGGCATACT<br>CTGCGACATCGTATAACGTTACTGGTTTCACATTCACCACCCTGAATTGACTCTCTTCCGGG<br>CGCTATCATGCCATACCGCGAAAGGTTTTGCGCCATTCGATGGTGTCCGGGATCTCGACGCT<br>CTCCCTTATGAAGTCTAACGCTGCTCTGGGCTAACTGTCGCGC |
| Receiver | pEcf11_3726 - mCherry - T7term - pLux - GFP_AAV - B0015 terminator - Lambda t0 terminator - AmpR - ColE1 origin - LacI | GCCTCCACACCGCTCGTCACATCCTGTGATCCACTCTTCATCCCGCTACGTAACACCTCTGC<br>ATCGCGAACCAAAACCAGGGATCCTGTCAATTTCCGCGATAGAGGAGGTAAAGATGCGTAAA<br>GTGAGCAAGGGCGAAGAAGATAACATGGCCATCATCAAGGAGTTCATGCGCTTCAAGGTTCA<br>CATGGAGGGCTCCGTGAACGGCCACGAGTTCGAGATCGAGGGCGAGGGCGAGGGCCGCCCCT<br>ACGAGGGCACCCAGACCGCCAAGCTGAAGGTGACCAAGGGTGGCCCCCTGCCCTTCGCCTGG<br>GACATCCTGTCCCCTCAGTTCATGTACGGCTCCAAGGCCTACGTGAAGCACCCCGCCGACAT<br>CCCCGACTACTTGAAGCTGTCCTTCCCCGAGGGCTTCAAGTGGGAGCGCGTGATGAACTTCG<br>AGGACGGCGGCGTGGTGACCGTGACCCAGGACTCCTCCCTGCAAGACGGCGAGTTCATCTAC<br>AAGGTGAAGCTGCGCGGCACCAACTTCCCCTCCGACGGCCCCGTAATGCAGAAGAAGACTAT<br>GGGCTGGGAGGCCTCCTCCGAGCGGATGTACCCCGAGGACGGCGCGCTGAAGGGCGAGATCA<br>AGCAGAGGCTGAAGCTGAAGGACGGCGGCCACTACGACGCTGAGGTCAAGACCACCTACAAG<br>GCCAAGAAGCCCGTGCAACTGCCCGGCGCGTACAACGTCAACATCAAGTTGGACATCACCTC<br>CCACAACGAGGACTACACCATCGTGGAACAGTACGAACGCGCCGAGGGCCGCCACTCCACCG<br>GCGGCATGGACGAGCTGTACAAGTAATAAAACAGTCGAGCCCAGCGTGGTTAAACACTCTAG<br>CATAACCCCTTGGGGCCTCTAAACGGGTCTTGAGGGGTTTTTTGCTGAAAGGAGGAACTATA<br>TCCGGATATCCCGCAAGAGGCCCGGCAGTACCGGCATAACCAAGCCTATGCCTACAGCATCC<br>AGGGTGACGGTGCCGAGGATGACGATGAGCGCATTGTTAGATTTCATACACGGTGCCTGACT<br>GCGTTAGCAATTTAACTGTGATAAACTACCGCATTAAAGCTTATCGATGATAAGCTGTCAAA<br>CATGAGAATTCTTGAAGACGAAAGGGCCTCGTGATACGCCTATTTTTATAGGTTAATGTCAT<br>GATAATAATGGTTTCTTAGACGTCAGGTGGCACTTTTCGGGGAAATGTGCGCGGAACCCCTA<br>AGACCTGTAGGATCGTACAGGTTTACGCAAGAAAATGGTTTGTTACTTTCGAATAAATACTA<br>GAGATTAAAGAGGAGAAATACTAGATGCGTAAAGGAGAAGAACTTTTCACTGGAGTTGTCCC<br>AATTCTTGTTGAATTAGATGGTGATGTTAATGGGCACAAATTTTCTGTCAGTGGAGAGGGTG<br>AAGGTGATGCAACATACGGAAAACTTACCCTTAAATTTATTTGCACTACTGGAAAACTACCT<br>GTTCCATGGCCAACACTTGTCACTACTTTCGGTTATGGTGTTCAATGCTTTGCGAGATACCC<br>AGATCATATGAAACAGCATGACTTTTTCAAGAGTGCCATGCCCGAAGGTTATGTACAGGAAA<br>GAACTATATTTTTCAAAGATGACGGGAACTACAAGACACGTGCTGAAGTCAAGTTTGAAGGT<br>GATACCCTTGTTAATAGAATCGAGTTAAAAGGTATTGATTTTAAAGAAGATGGAAACATTCT<br>TGGACACAAATTGGAATACAACTATAACTCACACAATGTATACATCATGGCAGACAAACAAA<br>AGAATGGAATCAAAGTTAACTTCAAAATTAGACACAACATTGAAGATGGAAGCGTTCAACTA<br>GCAGACCATTATCAACAAAATACTCCAATTGGCGATGGCCCTGTCCTTTTACCAGACAACCA<br>TTACCTGTCCACACAATCTGCCCTTTCGAAAGATCCCAACGAAAAGAGAGACCACATGGTCC<br>TTCTTGAGTTTGTAACAGCTGCTGGGATTACACATGGCATGGATGAACTATACAAAAGGCCT<br>GCAGCAAACGACGAAAACTACGCTGCTGCTGTTTAATAATACTAGAGCCAGGCATCAAATAA<br>AACGAAAGGCTCAGTCGAAAGACTGGGCCTTTCGTTTTATCTGTTGTTTGTCGGTGAACGCT<br>CTCTACTAGAGTCACACTGGCTCACCTTCGGGTGGGCCTTTCTGCGTTTATATACTAGTAGC<br>GGCCGCTGCAGGCATGCAAGCTTGCGGCCGCGTCGTGACTGGGAAAACCCTGGCGACTAGTC<br>TTGGACTCCTGTTGATAGATCCAGTAATGACCTCAGAACTCCATCTGGATTTGTTCAGAACG<br>CTCGGTTGCCGCCGGGCGTTTTTTATTGGTGAGAATCCAGGGGTCCCCAATAATTACGATTT<br>AAATTAGTAGCCCGCCTAATGAGCGGGCTTTTTTTTAATTCCCCTATTTGTTTATTTTTCTA<br>AATACATTCAAATATGTATCCGCTCATGAGACAATAACCCTGATAAATGCTTCAATAATATT<br>GAAAAAGGAAGAGTATGAGTATTCAACATTTCCGTGTCGCCCTTATTCCCTTTTTTGCGGCA<br>TTTTGCCTTCCTGTTTTTGCTCACCCAGAAACGCTGGTGAAAGTAAAAGATGCTGAAGATCA<br>GTTGGGTGCACGAGTGGGTTACATCGAACTGGATCTCAACAGCGGTAAGATCCTTGAGAGTT<br>TTCGCCCCGAAGAACGTTTTCCAATGATGAGCACTTTTAAAGTTCTGCTATGTGGCGCGGTA<br>TTATCCCGTGTTGACGCCGGGCAAGAGCAACTCGGTCGCCGCATACACTATTCTCAGAATGA<br>CTTGGTTGAGTACTCACCAGTCACAGAAAAGCATCTTACGGATGGCATGACAGTAAGAGAAT<br>TATGCAGTGCTGCCATAACCATGAGTGATAACACTGCGGCCAACTTACTTCTGACAACGATC<br>GGAGGACCGAAGGAGCTAACCGCTTTTTTGCACAACATGGGGGATCATGTAACTCGCCTTGA<br>TCGTTGGGAACCGGAGCTGAATGAAGCCATACCAAACGACGAGCGTGACACCACGATGCCTG |

| | | |
|---|---|---|
| | | CAGCAATGGCAACAACGTTGCGCAAACTATTAACTGGCGAACTACTTACTCTAGCTTCCCGG<br>CAACAATTAATAGACTGGATGGAGGCGGATAAAGTTGCAGGACCACTTCTGCGCTCGGCCCT<br>TCCGGCTGGCTGGTTTATTGCTGATAAATCTGGAGCCGGTGAGCGTGGGTCTCGCGGTATCA<br>TTGCAGCACTGGGGCCAGATGGTAAGCCCTCCCGTATCGTAGTTATCTACACGACGGGGAGT<br>CAGGCAACTATGGATGAACGAAATAGACAGATCGCTGAGATAGGTGCCTCACTGATTAAGCA<br>TTGGTAACTGTCAGACCAAGTTTACTCATATATACTTTAGATTGATTTAAAACTTCATTTTT<br>AATTTAAAAGGATCTAGGTGAAGATCCTTTTTGATAATCTCATGACCAAAATCCCTTAACGT<br>GAGTTTTCGTTCCACTGAGCGTCAGACCCCGTAGAAAAGATCAAAGGATCTTCTTGAGATCC<br>TTTTTTTCTGCGCGTAATCTGCTGCTTGCAAACAAAAAAACCACCGCTACCAGCGGTGGTTT<br>GTTTGCCGGATCAAGAGCTACCAACTCTTTTTCCGAAGGTAACTGGCTTCAGCAGAGCGCAG<br>ATACCAAATACTGTCCTTCTAGTGTAGCCGTAGTTAGGCCACCACTTCAAGAACTCTGTAGC<br>ACCGCCTACATACCTCGCTCTGCTAATCCTGTTACCAGTGGCTGCTGCCAGTGGCGATAAGT<br>CGTGTCTTACCGGGTTGGACTCAAGACGATAGTTACCGGATAAGGCGCAGCGGTCGGGCTGA<br>ACGGGGGGTTCGTGCACACAGCCCAGCTTGGAGCGAACGACCTACACCGAACTGAGATACCT<br>ACAGCGTGAGCTATGAGAAAGCGCCACGCTTCCCGAAGGGAGAAAGGCGGACAGGTATCCGG<br>TAAGCGGCAGGGTCGGAACAGGAGAGCGCACGAGGGAGCTTCCAGGGGGAAACGCCTGGTAT<br>CTTTATAGTCCTGTCGGGTTTCGCCACCTCTGACTTGAGCGTCGATTTTTGTGATGCTCGTC<br>AGGGGGGCGGAGCCTATGGAAAAACGCCAGCAACGCGGCCTTTTTACGGTTCCTGGCCTTTT<br>GCTGGCCTTTTGCTCACATGTTCTTTCCTGCGTTATCCCCTGATTCTGTGGATAACCGTATT<br>ACCGCCTTTGAGTGAGCTGATACCGCTCGCCGCAGCCGAACGACCGAGCGCAGCGAGTCAGT<br>GAGCGAGGAAGCGGAAGAGCGCCTGATGCGGTATTTTCTCCTTACGCATCTGTGCGGTATTT<br>CACACCGCATATATGGTGCACTCTCAGTACAATCTGCTCTGATGCCGCATAGTTAAGCCAGT<br>ATACACTCCGCTATCGCTACGTGACTGGGTCATGGCTGCGCCCCGACACCCGCCAACACCCG<br>CTGACGCGCCCTGACGGGCTTGTCTGCTCCCGGCATCCGCTTACAGACAAGCTGTGACCGTC<br>TCCGGGAGCTGCATGTGTCAGAGGTTTTCACCGTCATCACCGAAACGCGCGAGGCAGCTGCG<br>GTAAAGCTCATCAGCGTGGTCGTGAAGCGATTCACAGATGTCTGCCTGTTCATCCGCGTCCA<br>GCTCGTTGAGTTTCTCCAGAAGCGTTAATGTCTGGCTTCTGATAAAGCGGGCCATGTTAAGG<br>GCGGTTTTTTCCTGTTTGGTCACTGATGCCTCCGTGTAAGGGGGATTTCTGTTCATGGGGGT<br>AATGATACCGATGAAACGAGAGAGGATGCTCACGATACGGGTTACTGATGATGAACATGCCC<br>GGTTACTGGAACGTTGTGAGGGTAAACAACTGGCGGTATGGATGCGGCGGGACCAGAGAAAA<br>ATCACTCAGGGTCAATGCCAGCGCTTCGTTAATACAGATGTAGGTGTTCCACAGGGTAGCCA<br>GCAGCATCCTGCGATGCAGATCCGGAACATAATGGTGCAGGGCGCTGACTTCCGCGTTTCCA<br>GACTTTACGAAACACGGAAACCGAAGACCATTCATGTTGTTGCTCAGGTCGCAGACGTTTTG<br>CAGCAGCAGTCGCTTCACGTTCGCTCGCGTATCGGTGATTCATTCTGCTAACCAGTAAGGCA<br>ACCCCGCCAGCCTAGCCGGGTCCTCAACGACAGGAGCACGATCATGCGCACCCGTGGCCAGG<br>ACCCAACGCTGCCCGAGATGCGCCGCGTGCGGCTGCTGGAGATGGCGGACGCGATGGATATG<br>TTCTGCCAAGGGTTGGTTTGCGCATTCACAGTTCTCCGCAAGAATTGATTGGCTCCAATTCT<br>TGGAGTGGTGAATCCGTTAGCGAGGTGCCGCCGGCTTCCATTCAGGTCGAGGTGGCCCGGCT<br>CCATGCACCGCGACGCAACGCGGGGAGGCAGACAAGGTATAGGGCGGCGCCTACAATCCATG<br>CCAACCCGTTCCATGTGCTCGCCGAGGCGGCATAAATCGCCGTGACGATCAGCGGTCCAGTG<br>ATCGAAGTTAGGCTGGTAAGAGCCGCGAGCGATCCTTGAAGCTGTCCCTGATGGTCGTCATC<br>TACCTGCCTGGACAGCATGGCCTGCAACGCGGGCATCCCGATGCCGCCGGAAGCGAGAAGAA<br>TCATAATGGGGAAGGCCATCCAGCCTCGCGTCGCGAACGCCAGCAAGACGTAGCCCAGCGCG<br>TCGGCCGCCATGCCGGCGATAATGGCCTGCTTCTCGCCGAAACGTTTGGTGGCGGGACCAGT<br>GACGAAGGCTTGAGCGAGGGCGTGCAAGATTCCGAATACCGCAAGCGACAGGCCGATCATCG<br>TCGCGCTCCAGCGAAAGCGGTCCTCGCCGAAAATGACCCAGAGCGCTGCCGGCACCTGTCCT<br>ACGAGTTGCATGATAAAGAAGACAGTCATAAGTGCGGCGACGATAGTCATGCCCCGCGCCCA<br>CCGGAAGGAGCTGACTGGGTTGAAGGCTCTCAAGGGCATCGGTCGAGATCCCGGTGCCTAAT<br>GAGTGAGCTAACTTACATTAATTGCGTTGCGCTCACTGCCCGCTTTCCAGTCGGGAAACCTG<br>TCGTGCCAGCTGCATTAATGAATCGGCCAACGCGCGGGGAGAGGCGGTTTGCGTATTGGGCG<br>CCAGGGTGGTTTTTCTTTTCACCAGTGAGACGGGCAACAGCTGATTGCCCTTCACCGCCTGG<br>CCCTGAGAGAGTTGCAGCAAGCGGTCCACGCTGGTTTGCCCCAGCAGGCGAAAATCCTGTTT<br>GATGGTGGTTAACGGCGGGATATAACATGAGCTGTCTTCGGTATCGTCGTATCCCACTACCG<br>AGATATCCGCACCAACGCGCAGCCCGGACTCGGTAATGGCGCGCATTGCGCCCAGCGCCATC<br>TGATCGTTGGCAACCAGCATCGCAGTGGGAACGATGCCCTCATTCAGCATTTGCATGGTTTG<br>TTGAAAACCGGACATGGCACTCCAGTCGCCTTCCCGTTCCGCTATCGGCTGAATTTGATTGC<br>GAGTGAGATATTTATGCCAGCCAGCCAGACGCAGACGCGCCGAGACAGAACTTAATGGGCCC<br>GCTAACAGCGCGATTTGCTGGTGACCCAATGCGACCAGATGCTCCACGCCCAGTCGCGTACC<br>GTCTTCATGGGAGAAAATAATACTGTTGATGGGTGTCTGGTCAGAGACATCAAGAAATAACG<br>CCGGAACATTAGTGCAGGCAGCTTCCACAGCAATGGCATCCTGGTCATCCAGCGGATAGTTA<br>ATGATCAGCCCACTGACGCGTTGCGCGAGAAGATTGTGCACCGCCGCTTTACAGGCTTCGAC<br>GCCGCTTCGTTCTACCATCGACACCACCACGCTGGCACCCAGTTGATCGGCGCGAGATTTAA<br>TCGCCGCGACAATTTGCGACGGCGCGTGCAGGGCCAGACTGGAGGTGGCAACGCCAATCAGC<br>AACGACTGTTTGCCCGCCAGTTGTTGTGCCACGCGGTTGGGAATGTAATTCAGCTCCGCCAT<br>CGCCGCTTCCACTTTTTCCCGCGTTTTCGCAGAAACGTGGCTGGCCTGGTTCACCACGCGGG<br>AAACGGTCTGATAAGAGACACCGGCATACTCTGCGACATCGTATAACGTTACTGGTTTCACA<br>TTCACCACCCTGAATTGACTCTCTTCCGGGCGCTATCATGCCATACCGCGAAAGGTTTTGCG<br>CCATTCGATGGTGTCCGGGATCTCGACGCTCTCCCTTATGCGACTCCTGCATTAGGAAGCAG<br>CCCAGTAGTAGGTTGAGGCCGTTGAGCACCGCCGCCGCAAGGAATGGTGCATGCAAGGAGAT<br>GGCGCCCAACAGTCCCCCGGCCACGGGGCCTGCCACCATACCCACGCCGAAACAAGCGCTCA<br>TGAGCCCGAAGTGGCGAGCCCGATCTTCCCCATCGGTGATGTCGGCGATATAGGCGCCAGCA<br>ACCGCACCTGTGGCGCCGGTGATGCCGGCCACGATGCGTCCGGCGTAGAGGATCGAGATCTC<br>GAACGTGTACGGGCTATCTGGCTTTCGTTGCGC |

# References

1. Untergasser, A., Cutcutache, I., Koressaar, T., Ye, J., Faircloth, B.C., Remm, M. and Rozen, S.G. (2012) Primer3--new capabilities and interfaces. *Nucleic Acids Res.*, **40**, e115.
2. Malinen, E., Kassinen, A., Rinttilä, T. and Palva, A. (2003) Comparison of real-time PCR with SYBR Green I or 5′-nuclease assaysand dot-blot hybridization with rDNA-targeted oligonucleotide probes in quantificationof selected faecal bacteria. *Microbiology*, **149**, 269-277.
3. Seely, S.M. and Gagnon, M.G. (2022) Mechanisms of ribosome recycling in bacteria and mitochondria: a structural perspective. *RNA Biol*, **19**, 662-677.
4. Gunisova, S., Hronova, V., Mohammad, M.P., Hinnebusch, A.G. and Valasek, L.S. (2018) Please do not recycle! Translation reinitiation in microbes and higher eukaryotes. *FEMS Microbiol Rev*, **42**, 165-192.
5. Chemla, Y., Peeri, M., Heltberg, M.L., Eichler, J., Jensen, M.H., Tuller, T. and Alfonta, L. (2020) A possible universal role for mRNA secondary structure in bacterial translation revealed using a synthetic operon. *Nat. Commun.*, **11**, 4827.
6. Ferro, R., Rennig, M., Hernandez-Rollan, C., Daley, D.O. and Norholm, M.H.H. (2018) A synbio approach for selection of highly expressed gene variants in Gram-positive bacteria. *Microb. Cell. Fact.*, **17**, 37.
7. Humbard, M.A., Surkov, S., De Donatis, G.M., Jenkins, L.M. and Maurizi, M.R. (2013) The N-degradome of Escherichia coli: limited proteolysis in vivo generates a large pool of proteins bearing N-degrons. *J Biol Chem*, **288**, 28913-28924.
